# Evaluation of electrochemiluminescence immunoassays for immunosuppressive drugs on the Roche cobas e411 analyzer

**DOI:** 10.12688/f1000research.12775.2

**Published:** 2017-12-12

**Authors:** Angela W.S. Fung, Michael J. Knauer, Ivan M. Blasutig, David A. Colantonio, Vathany Kulasingam

**Affiliations:** 1 Department of Laboratory Medicine and Pathobiology, University of Toronto, Toronto, ON, M5S 1A8, Canada; 2 Department of Pathology and Laboratory Medicine, St. Paul’s Hospital, Vancouver, BC, V6Z 1Y6, Canada; 3 Lifelabs Medical Laboratories, Toronto, ON, M9W 6J6, Canada; 4 Division of Biochemistry, Children’s Hospital of Eastern Ontario, Ottawa, ON, K1H 8L1, Canada; 5 Department of Pediatric Laboratory Medicine, The Hospital for Sick Children, Toronto, ON, M5G 1X8, Canada; 6 Department of Clinical Biochemistry, University Health Network, Toronto, ON, M5G 2C4, Canada

**Keywords:** Cyclosporine, tacrolimus, sirolimus, immunoassay, ECLIA

## Abstract

**Background**:  Therapeutic drug monitoring of immunosuppressant drugs are used to monitor drug efficacy and toxicity and to prevent organ transplant rejection. This study evaluates the analytical performance of semi-automated electrochemiluminescence immunoassays (ECLIA) for cyclosporine (CSA), tacrolimus (TAC) and sirolimus (SRL) on the Roche cobas e 411 analyzer at a major transplant hospital to assess method suitability and limitations.

**Methods**: Residual whole blood samples from patients undergoing immunosuppressant therapy were used for evaluation. Imprecision, linearity, functional sensitivity, method comparisons and lot-to-lot comparisons were assessed.

**Results**: Total imprecision ranged from 3.3 to 7.1% for CSA, 3.9 to 9.4% for TAC, and 4.6 to 8.2% for SRL. Linearity was verified from 30.0 to 960.9 μg/L for CSA, from 1.1 to 27.1 μg/L for TAC, and from 0.5 to 32.3 µg/L for SRL. The functional sensitivity met the manufacturer’s claims and was determined to be <6.5 μg/L for CSA, 1.1 μg/L for TAC, and <0.1 µg/L for SRL (CV≤20%). Deming regression analysis of method comparisons with the ARCHITECT immunoassay yielded slopes of 0.917 (95%CI: 0.885-0.949) and r of 0.985 for CSA, 0.938 (95%CI: 0.895-0.981) and r of 0.974 for TAC, and 0.842 (0.810-1.110) and r of 0.982 for SRL. Deming regression analysis of comparisons with the LC–MS/MS method yielded slopes of 1.331 (95%CI: 1.167-1.496) and r of 0.969 for CSA, 0.924 (95%CI: 0.843-1.005) and r of 0.984 for TAC, and 0.971 (95%CI: 0.913-1.030) and r of 0.993 for SRL.

**Conclusions**: The cobas e 411 ECLIA for CSA, TAC, and SRL have acceptable precision, linearity, and functional sensitivity. The method comparisons correlated well with the ARCHITECT immunoassay and LC–MS/MS and is fit for therapeutic drug monitoring

## Introduction

Immunosuppressive drugs (ISD), such as the calcineurin inhibitors (cyclosporine (CSA) and tacrolimus (TAC)) and mammalian target of rapamycin (mTOR) inhibitors (sirolimus (SRL) and everolimus), are critical to the maintenance of solid organ transplantation ^1^. CSA is a cyclic undecapeptide and TAC (also known as FK-506) is a macrolide lactone. CSA binds to cyclophilin A/B and inhibits calcineurin. TAC binds to FK506-binding protein 12 (FKBP-12) to form the calcineurin inhibitory complex. Inhibition of calcineurin, a serine/threonine phosphatase, leads to altered calcium-dependent signal transduction, and decreases T-cell activation and downregulates anti-inflammatory response-related genes ^2, 3^. SRL (also known as rapamycin) is a 31-membered macrolide antibiotic that binds to FKBP-12 and allosterically targets the mTOR pathway, inhibiting cell cycle progression, T-cell proliferation and differentiation ^2^. SRL has structural similarities to TAC and competes with TAC for FKBP-12 binding ^1, 2^. All three ISDs are characterized by having variable absorption, poor bioavailability, strong affinity to blood proteins, leukocytes, and/or erythrocytes, and metabolism via cytochrome CYP3A4/5 and efflux transport by P-glycoprotein ^2^.

Therapeutic drug monitoring is a mainstay in immunosuppressant therapy. ISDs have narrow therapeutic ranges ^2^, high interindividual variability in pharmacokinetics and pharmacogenetics ^4, 5^, susceptibility to food- and drug-drug interactions ^6^, and adverse consequences if plasma drug levels are not maintained ^5, 7^. Similar toxic effects have been described for CSA and TAC, due to their overlapping mechanism of action, and includes nephrotoxicity, hypertension, and neurotoxicity ^2^. TAC is a more potent calcineurin inhibitor than CSA, due to increased affinity for FKBP-12 and the advantage of decreased nephrotoxicity, risk of hyperlipidemia and hypertension ^1, 2, 8^. TAC, however, is more likely to cause post-transplantation diabetes ^1, 9, 10^. SRL does not cause renal toxicities; however, long-term SRL use can induce leukopenia, thrombocytopenia, and dyslipidemia ^11, 12^. The target therapeutic range for each ISD may vary depending on type of organ transplanted, time from transplantation, co-administered drugs, and method of analysis.

Recently, semi-automated electrochemiluminescence immunoassays (ECLIA) for the quantification of CSA, TAC, and SRL in whole blood were developed and made available by Roche Diagnostics (GmbH, Mannheim, Germany) ^13, 14^. In this study, we evaluated the analytical performance of ECLIA method for CSA, TAC, and SRL on the Roche cobas e411 analyzer and compared to the commonly used chemiluminescent microparticle immunoassay (CMIA) method on the Abbott ARCHITECT *i*2000 analyzer (Abbott Laboratories, Abbott Park, IL, USA). This is the first report on the evaluation of ECLIA SRL, and compares the performance of all three ISDs together.

## Methods

### Specimen source and handling

Ethics approval for this study was waived by the Research Ethics Board at the University Health Network in Toronto, Ontario, Canada (16-6312) for use of routine collected specimens for the evaluation of method performance. Residual EDTA whole blood specimens from 300 patients undergoing immunosuppressant therapy (either cyclosporine, tacrolimus, or sirolimus) at the University Health Network, and CAP proficiency testing samples were used in this evaluation. Samples were collected and analyzed by the Abbott CMIA within the same day, then stored as per manufacturer recommendations and analyzed later by the Roche ECLIA and LC-MS/MS methods. Samples were thawed and equilibrated to room temperature for 30 minutes and mixed well prior to analysis. In accordance with stability studies on whole blood ISD specimens, samples were analyzed within three months of collection and did not undergo more than two freeze-thaw cycles ^15– 18^.

### Electrochemiluminescence immunoassay (ECLIA) method on Roche cobas e 411

The cobas ECLIAs (Roche Diagnostics GmbH, Mannheim, Germany) for CSA, TAC, and SRL are based on the competition of analyte in sample with a ruthenium-labeled analogue. A voltage is applied and electrochemiluminescence signal is detected. Testing was performed according to the manufacturer’s instructions. Briefly, the samples (calibrators, QC, whole blood samples) were manually pretreated by combining 300 µL of whole blood with 300 µL of Universal ISD Sample Pretreatment Reagent (containing zinc sulfate and methanol) and vortexed for 10 seconds to lyse the red blood cells, precipitate proteins and extract the analyte. The samples were centrifuged for 4 minutes at 15,000 x g, and the supernatant was decanted for analysis. Analysis was performed within 30 minutes of preparation to prevent evaporation of the extracted samples. The ECLIA assays were calibrated as per manufacturer’s instruction by a 2-point calibration using calibrators traceable to pure standard materials reconstituted in whole blood matrix by gravimetrical methods.

### Chemiluminescent microparticle immunoassay (CMIA) method on Abbott ARCHITECT i2000

The ARCHITECT CMIAs (Abbott Laboratories, Abbott Park, IL, USA) for CSA, TAC, and SRL are based on competition of analyte in sample with acridinium-labeled analogue. The samples were manually pretreated according to the manufacturer’s instructions and site-specific standard operating procedures. For CSA, 100 µL of Cyclosporine Solubilizing Reagent (4% saponin) and 400 µL of Cyclosporine Precipitation Reagent (zinc sulfate in methanol and ethylene glycol) was added to 200 µL of sample ^19, 20^. For TAC, 200 µL of sample was mixed with 200 µL of Tacrolimus Precipitation Reagent (zinc sulfate in methanol) ^21, 22^. For SRL, 150 µL of sample was mixed with 300 µL of Sirolimus Precipitation Reagent (zinc sulfate in >50% v/v DMSO and ethylene glycol), vortexed and heated at 42°C for 10 minutes ^23, 24^. All ISD samples were then vortexed for 10 seconds and centrifuged for 4 minutes at 15,000 x g. The supernatants were decanted into labelled tubes and assayed within 30 minutes of sample preparation. The ARCHITECT CMIAs are calibrated according to the site-specific standard operation procedures and manufacturer’s instructions, with a 6-point 4-parameter logistic curve fit (4PLC, y-weighted) that is traceable to pure standard materials in a whole blood matrix by gravimetrical methods. Internal QC was evaluated with Bio-Rad Lyphochek Whole Blood ISD Controls levels 1, 3, and 4.

### Electrospray ionization liquid chromatography tandem mass spectrometry (ESI-LC-MS/MS) method

The ESI-LC-MS/MS MRM method for CSA, TAC, and SRL were analyzed on a 4000 QTrap mass spectrometer (SCIEX) at the Hospital for Sick Children (Toronto, ON, Canada). Samples were pretreated by mixing 40 µL of sample with 100 µL of sample pretreatment reagent consisting of 0.04M zinc sulfate, and internal standards 100.0 µg/L cyclosporine D and 10.0 µg/L ascomycin in methanol. Samples were vortexed and centrifuged for 5 minutes at 15, 000 x g to obtain the supernatant for analysis. The analyte is separated by liquid chromatography (Nexera X2 Shimadzu) with a reverse phase C _18_ column (Phenomenex, 4 x 3.0 mm at 45°C) and gradient elution from 100% B to 50% B (Buffer A: 2 mM ammonia acetate and 0.1% formic acid in water and Buffer B: 2 mM ammonia acetate and 0.1% formic acid in methanol) at a flowrate of 650 µL/min and electrospray ionization into the mass spectrometer. The following precursor/production pairs in positive ion mode were used 1220.8/1203.8 *m/z* for CSA, 821.5/768.5 *m/z* for TAC, and 931.6/864.5 *m/z* for SRL. CSA and TAC were calibrated with a 6-point calibration curve using Emit 2000 CSA or TAC specific calibrators (Syva Company, Siemens Healthcare). SRL was calibrated with a 6-point calibration curve using 6Plus1 Multilevel immunosuppressant calibrators (Chromsystems). There is generally a lack of certified reference materials for TDM-relevant drugs, including the ISDs. There is currently only one ISD certified reference material for tacrolimus in whole blood (ERM-DA110a), and current efforts are directed towards standardization ^25^. Internal QC were evaluated with Bio-Rad Lyphochek Whole Blood ISD Controls levels 1, 3, and 4.

### Imprecision

Three levels of manufacturer multi-analyte QC materials (Roche Diagnostics PreciControl ISD levels 1, 2, and 3) and third-party multi-analyte QC materials (Bio-Rad Lyphochek Whole Blood ISD Controls levels 1, 3, and 4) were analyzed. QC samples were prepared and measured in duplicate, one run per day over 10 days. The acceptance criterion for total imprecision was based on the recommendation of the International Association of Therapeutic Drug Monitoring and Clinical Toxicology (IATDMCT) expert consensus group of ≤ 10% ^25^.

### Functional sensitivity

Residual patient sample with levels 2–3 times the claimed limit of quantification (LoQ) was used to generate a series of dilutions with blank whole blood. The neat sample and dilutions were measured in triplicates within one day. The precision profile curve was used to calculate the LoQ concentration corresponding to a CV of 20% with the upper 95% confidence limit.

### Linearity

Since there is a lack of elevated CSA and TAC patient specimen, CSA and TAC linearity were assessed using CAP EQA linearity materials (6 concentrations measured in duplicate). SRL linearity was assessed using a patient sample above the upper measuring range diluted with blank whole blood to 6 concentrations and measured in duplicate. The acceptance criterion was defined as slope of 1.00 ± 0.05 and deviation <10%.

### Method comparison

Method comparison experiments were assessed where anonymized residual patient samples spanning the analytical measuring range for each analyte were measured once per method. CSA samples concentrations ranged from 41.0 to 1808.0 μg/L, TAC ranged from 2.1 to 30.0 μg/L, and SRL ranged from 1.8 to 34.6 μg/L as determined by ARCHITECT CMIA. Roche ECLIA measurements were compared to ARCHITECT CMIA (n=100). To further elucidate the accuracy between immunoassays, a subset of samples was also analyzed by LC-MS/MS (n=20). Lot-to-lot assessment was also performed between two lots of reagents for each ISD (n=20). The slope, intercept, correlation coefficient *r* were analyzed by Deming regression analysis. The acceptance criteria for method comparison were defined as a slope of 1.00 ± 0.15 and *r* of ≥ 0.95, meanwhile for lot-to-lot comparison were defined as a slope of 1.00 ± 0.05 and *r* of ≥ 0.95.

### Statistical analysis

Microsoft Excel (version 1708, Microsoft Office) and/or EP Evaluator (version 7.0.0.307, Data Innovations) were used for statistical analysis.

## Results and discussion

To assess imprecision, three levels of manufacturer (Roche PreciControl) and third-party (Bio-Rad Lyphochek) multi-analyte QC materials were analyzed using one lot of reagents in duplicate, one run per day over 10 days ( Table 1). For the PreciControl, the total imprecision was <7.1% for CSA, <9.4% for TAC, and <5.6% for SRL. Imprecision for CSA and TAC were comparable to other studies ^13, 14^. Our study additionally evaluated third-party QC performance on ECLIA ISD assays, a total imprecision of <4.7% for CSA, <6.3% for TAC, and <8.2% for SRL were determined. The imprecision goal of ≤10%, based on the recommendation of the IATDMCT expert consensus group, was achieved for all QC samples ^25^. Note that this imprecision study was performed using a single reagent lot and may not represent variations due to other variables such as changes in operator, calibrator and reagent lots, and ambient operating conditions. Overall, the ECLIA methods demonstrate acceptable precision.

**Table 1.** Total imprecision for cyclosporine, tacrolimus, and sirolimus determined by the ECLIA method (duplicate per run, 1 run per day for 10 days).

	Samples	Mean Conc. (μg/L) *	Total CV (%)
Cyclosporine (CSA)	Roche ISD L1	63.1	7.1
	Roche ISD L2	271.1	5.1
	Roche ISD L3	976.9	3.8
	Bio-Rad WB ISD L1	67.3	4.7
	Bio-Rad WB ISD L3	346.5	4.5
	Bio-Rad WB ISD L4	740.1	3.3
Tacrolimus (TAC)	Roche ISD L1	2.5	9.4
	Roche ISD L2	9.2	6.9
	Roche ISD L3	16.9	4.1
	Bio-Rad WB ISD L1	4.1	6.3
	Bio-Rad WB ISD L3	7.8	5.3
	Bio-Rad WB ISD L4	15.2	3.9
Sirolimus (SRL)	Roche ISD L1	3.4	4.6
	Roche ISD L2	8.7	5.6
	Roche ISD L3	15.4	4.7
	Bio-Rad WB ISD L1	5.9	4.7
	Bio-Rad WB ISD L3	9.5	5.7
	Bio-Rad WB ISD L4	14.2	8.2

*Conventional unit: 1 µg/L = 1 ng/mL

The ECLIA methods offer a wider linear analytical measuring range for CSA and TAC than CMIA methods. ECLIA CSA, TAC, and SRL were linear up to 960.9 μg/L, 27.1 μg/L, and 32.3 μg/L, respectively. The higher upper limit allows TDM and pharmacokinetic analysis of ISD at different time points and peak concentrations offering additional flexibility ^26, 27^.

The claimed functional sensitivity of the ECLIA ISD methods are improved for TAC and SRL compared to CMIA ISD methods. The functional sensitivities were assessed and the precision profile was used to calculate the LoQ corresponding to a CV of 20% with upper 95% confidence limit. The functional sensitivities were determined to be <6.5 μg/L for CSA, 1.1 μg/L for TAC, and <0.1 μg/L for SRL, which meets the 2007 European consensus guideline and IATDMCT expert consensus group recommended LoQ of 20.0 μg/L for CSA and a LoQ of 1.0 μg/L for both TAC and SRL ^25, 28^.

**Figure 1.**
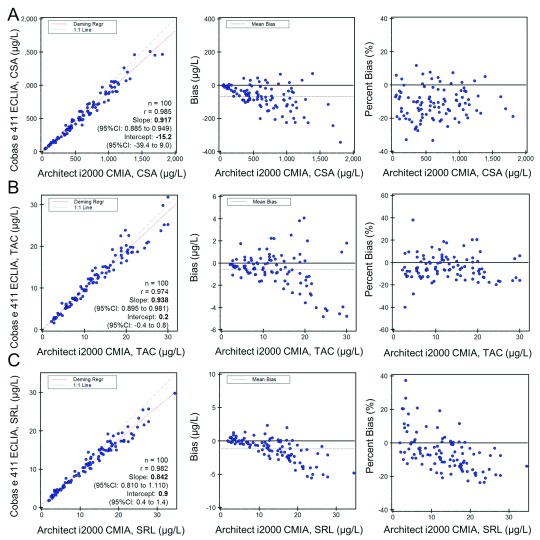
Method comparisons and Bland-Altman plots for ( **A**) cyclosporine (CSA), ( **B**) tacrolimus (TAC), and ( **C**) sirolimus (SRL) between cobas e411 ECLIA and ARCHITECT i2000 CMIA.

For method comparison, anonymized residual patient samples spanning the analytical measuring range for each analyte were measured. CSA samples concentrations ranged from 41.0 to 1808.0 μg/L, TAC ranged from 2.1 to 30.0 μg/L, and SRL ranged from 1.8 to 34.6 μg/L as determined by CMIA. ECLIA ISDs measurements were compared to CMIA ISDs (n=100). The acceptance criteria were defined as a slope of 1.00 ± 0.15 and *r* of ≥ 0.95. Figure 1 shows the Deming regression and Bland-Altman analysis for CSA, TAC, and SRL. ECLIA and CMIA CSA ( Figure 1A) showed good agreement with a slope of 0.917 (95% CI: 0.885-0.949), intercept of -15.2 (95% CI: -39.4-9.0), and *r* of 0.985. For TAC, the ECLIA TAC also showed good agreement with CMIA TAC ( Figure 1B) with a slope of 0.938 (95% CI: 0.895-0.981), intercept of 0.2 (95% CI -0.4-0.8), and *r* of 0.974. Similar trends were observed by others (slopes of 0.87 for CSA, and 0.96-0.98 for TAC) ^14, 29^. Reported for the first time, method comparison of ECLIA and CMIA SRL ( Figure 1C) showed a slope of 0.842 (95% CI: 0.810-1.110), intercept of 0.9 (95% CI: 0.4-1.4), and *r* of 0.982. Overall, all three ECLIA ISDs met our acceptance criteria, with SRL slightly exceeding the limit for the slope.

**Figure 2.**
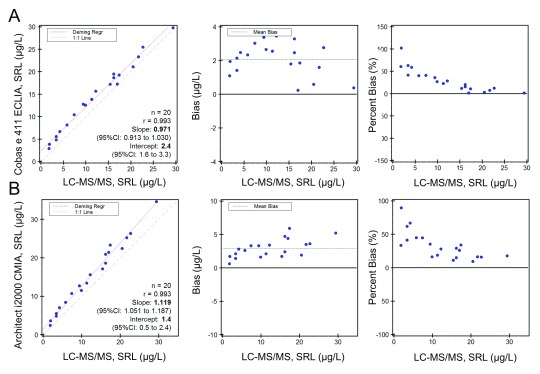
Method comparisons and Bland-Altman plots for sirolimus (SRL) between cobas e411 ECLIA and LC-MS/MS.

To further examine ECLIA performance with CMIA, a subset of samples was also analyzed by LC-MS/MS (n=20). For both CSA immunoassays, a positive bias was observed when compared with LC-MS/MS, similarly observed by others ^13, 29, 30^ ( Figure S1). For TAC, both the ECLIA and CMIA TAC had good agreement with LC-MS/MS, also similarly observed by others for different cohorts of solid organ transplant ^14, 21, 29^ ( Figure S2). Reported for the first time, the ECLIA SRL compared to LC-MS/MS showed a slope of 0.971 (95% CI: 0.913-1.030), intercept of 2.4 (95% CI: 1.6-3.3), and *r* of 0.993 ( Figure 2). Meanwhile, CMIA SRL compared to LC-MS/MS showed a slope 1.119 (95% CI: 1.051-1.187), intercept of 1.4 (95% CI: 0.5-2.4), and *r* of 0.993. Based on our small sample size, both ECLIA and CMIA SRL generally showed good correlation with LC-MS/MS, with ECLIA SRL with better agreement to LC-MS/MS.

For lot-to-lot comparisons, Deming regression analysis of 20 residual patient samples tested by 2 lots of reagent for CSA shows a slope of 0.998 (95% CI: 0.967-1.029), intercept of 8.5 (95% CI: -10.2-27.2), and *r* of 0.998. Lot-to-lot comparison for TAC shows a slope of 0.972 (95% CI: 0.936-1.008), intercept of -0.4 (95% CI: -0.8-0.0), and *r* of 0.997. And lot-to-lot comparison for SRL shows a slope of 0.913 (95% CI: 0.841-0.985), intercept of 0.1 (95% CI: -0.9-1.2), and *r* of 0.988. All three ISDs had good correlation between 2 different lots of reagents.

Evaluation on practical considerations included ease-of-use, throughput, and workflow of the method. The sample pretreatment for the ECLIA method is faster, simpler, and more convenient than CMIA method due to the use of a single universal sample pretreatment reagent and protocol for all three ISDs. Additionally, there is no heating step for the SRL ECLIA method, which leads to a simpler workflow. The ECLIA universal sample pretreatment reagent and protocol would enable better workflow, simpler sample handling and inventory control. The ECLIA method has an assay time of 18 minutes compared to CMIA of 30 minutes. Both ECLIA and CMIA have a lot calibration stability of approximately one month, thus requiring similar calibration frequency. Together, the needs of the individual clinical laboratory will dictate whether some of these practical considerations play a role in the method selection.

File containing the raw data and a table of contents for the data file Click here for additional data file. Copyright: © 2017 Fung AWS et al. 2017 Data associated with the article are available under the terms of the Creative Commons Zero "No rights reserved" data waiver (CC0 1.0 Public domain dedication).

## Conclusion

In conclusion, the overall analytical evaluation of the ECLIA method for CSA, TAC, and SRL met acceptable performance. ECLIA CSA showed better precision than our current CMIA CSA. ECLIA CSA and TAC showed better linearity range, and ECLIA TAC and SRL showed better functional sensitivity than CMIA methods. Method comparisons showed good correlations and agreement between ECLIA ISDs and CMIA ISDs.

## Data availability

The data referenced by this article are under copyright with the following copyright statement: Copyright: © 2017 Fung AWS et al.

Data associated with the article are available under the terms of the Creative Commons Zero "No rights reserved" data waiver (CC0 1.0 Public domain dedication). 

Dataset 1: File containing the raw data and a table of contents for the data file. doi, 10.5256/f1000research.12775.d180033 ^31^

## Supplementary material

Figure S1: Method comparisons and Bland-Altman plots for cyclosporine (CSA) between (A) cobas e 411 ECLIA and LC-MS/MS, and (B) ARCHITECT i2000 CMIA and LC-MS/MS.

Figure S2: Method comparisons and Bland-Altman plots for tacrolimus (TAC) between (A) cobas e 411 ECLIA and LC-MS/MS, and (B) ARCHITECT i2000 CMIA and LC-MS/MS.
