# Supplementary figures and images for: Evaluation of electrochemiluminescence immunoassays for immunosuppressive drugs on the Roche cobas e411 analyzer
Source: F1000Res. 2017 Dec 12;6:1832. Originally published 2017 Oct 13. [Version 2] doi: 10.12688/f1000research.12775.2 (PMC5710386; doi:10.12688/f1000research.12775.2)

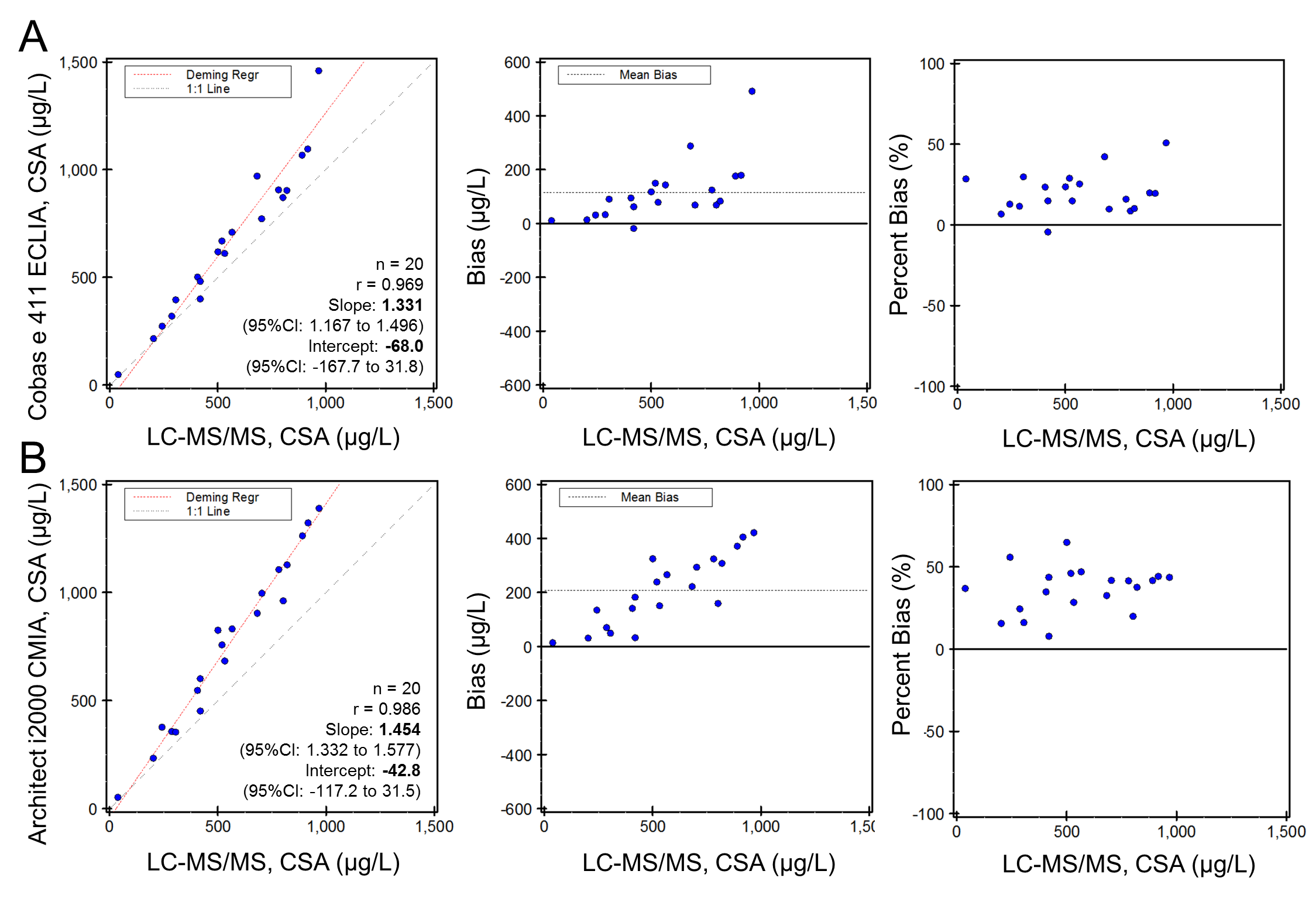

Supplement: Supplementary file 2 [file f1000research-6-14595-s0001.tgz › 849f3aad-6c07-4126-8f82-bba82cdc71fb.tif]

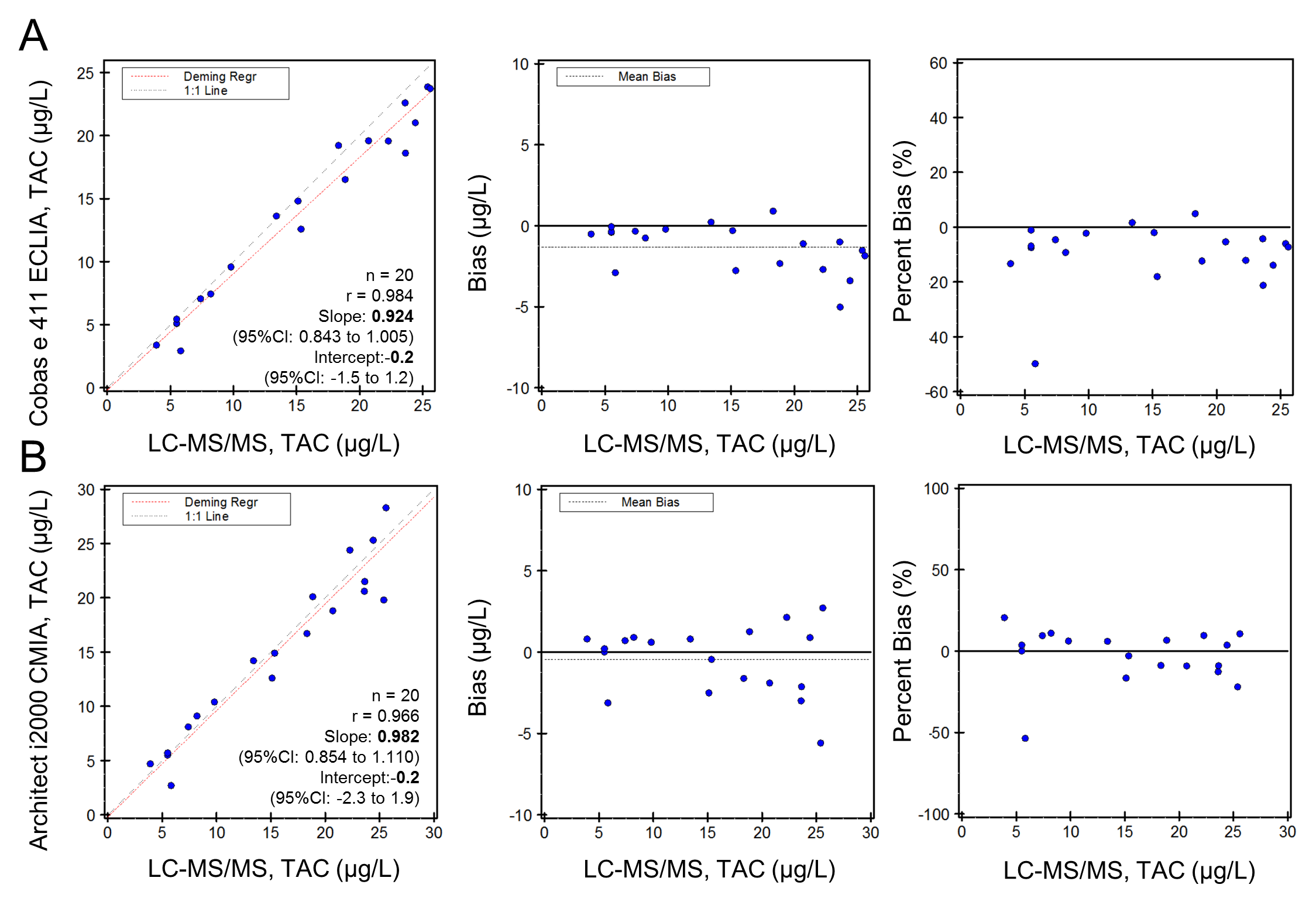

Supplement: Supplementary file 3 [file f1000research-6-14595-s0002.tgz › 9d8661c8-fe86-4e3d-8009-15e36ce33450.tif]
