# Supplementary material for: Evaluation of electrochemiluminescence immunoassays for immunosuppressive drugs on the Roche cobas e411 analyzer
Source: F1000Res. 2017 Dec 12;6:1832. Originally published 2017 Oct 13. [Version 2] doi: 10.12688/f1000research.12775.2 (PMC5710386; doi:10.12688/f1000research.12775.2)
Supplement: File containing the raw data and a table of contents for the data file [file f1000research-6-14595-s0000.tgz › 5482c4c4-1da7-4278-a215-5152bd6e3c64_Dataset_1.zip!/Dataset 1/Raw data.pdf]

Simple Precision Summary

Instrument	Analyte	Sample	N	Mean	SD	CV
e411	✔ Cyclosporine	BioRad 1	20 of 20	67.29	3.13	4.7%
	✔ Cyclosporine	BioRad 3	20 of 20	346.51	15.67	4.5%
	✔ Cyclosporine	BioRad 4	20 of 20	740.10	24.13	3.3%
	✔ Cyclosporine	ISD 1	20 of 20	63.09	4.46	7.1%
	✔ Cyclosporine	ISD 2	20 of 20	271.10	13.93	5.1%
	✔ Cyclosporine	ISD 3	20 of 20	976.90	37.12	3.8%
	✔ Tacrolimus	BioRad 1	20 of 20	4.10	0.26	6.3%
	✔ Tacrolimus	BioRad 3	20 of 20	7.79	0.41	5.3%
	✔ Tacrolimus	BioRad 4	20 of 20	15.24	0.59	3.9%
	✔ Tacrolimus	ISD 1	20 of 20	2.48	0.23	9.4%
	✔ Tacrolimus	ISD 2	20 of 20	9.16	0.63	6.9%
	✔ Tacrolimus	ISD 3	20 of 20	16.88	0.69	4.1%

◈ Required parameters are missing.

✘ Experiment 'fails' (or not enough results).

✔ Experiment 'passes' (or adequate number of results).

Copyright 1991-2005 David G. Rhoads Associates, Inc.

Cyclosporine

Instrument: e411

Sample Name: BioRad 1

Simple Precision

Precision Statistics

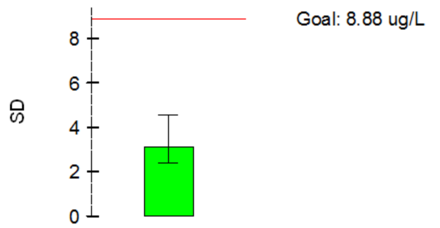

Standard Deviation (SD)	3.13
Passes?	Yes
95% Confidence for SD	2.38 to 4.57
Coefficient of Variation (CV)	4.7%
Mean	67.29 ug/L
Number of Specimens (N)	20 of 20
Number of Outliers	--

Precision Plot

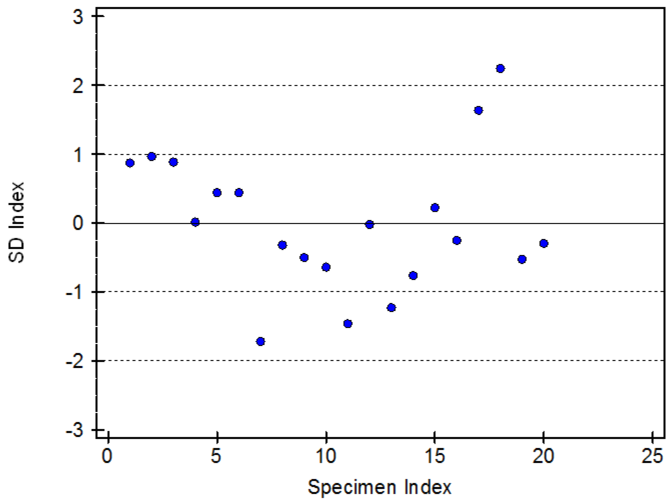

User's Specifications

Allowable Total Error:	20.0%
Random Error Budget:	66%
Allowable Random Error:	13.2%
Screen for Outliers?	No (N<25)

Supporting Data

Analyst:	MK
Expt. Date:	27 May 2015
Units:	ug/L
95% CI for mean:	65.82 to 68.75
2 SD Range:	61.03 to 73.55
Comment:	

Precision Data

Index	Result	Index	Result	Index	Result	Index	Result
1	70.0	6	68.7	11	62.7	16	66.5
2	70.3	7	61.9	12	67.2	17	72.4
3	70.1	8	66.3	13	63.4	18	74.3
4	67.3	9	65.7	14	64.9	19	65.6
5	68.7	10	65.3	15	68.0	20	66.4

O: outliers X: excluded from calculations

Accepted by:

Signature ______________________ Date ______________

Copyright 1991-2005 David G. Rhoads Associates, Inc.

EP Evaluator

Biochemistry -- University Health Network

Cyclosporine

Instrument: e411

Sample Name: BioRad 3

Simple Precision

Precision Statistics

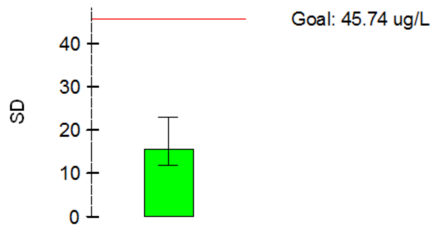

Standard Deviation (SD)	15.67
Passes?	Yes
95% Confidence for SD	11.92 to 22.89
Coefficient of Variation (CV)	4.5%
Mean	346.51 ug/L
Number of Specimens (N)	20 of 20
Number of Outliers	--

Precision Plot

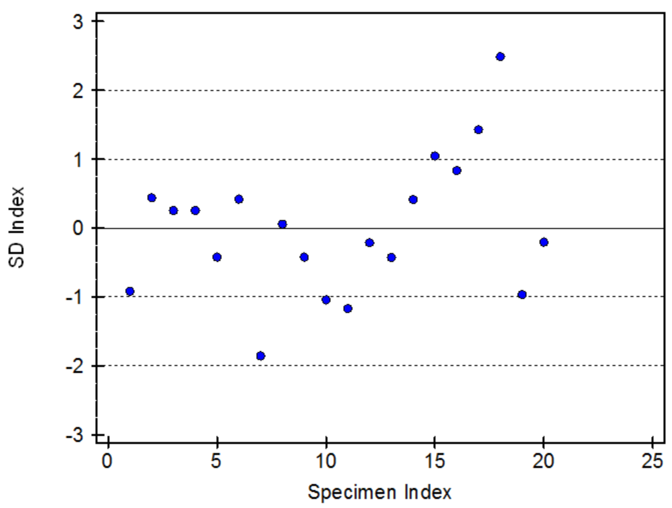

User's Specifications

Allowable Total Error:	20.0%
Random Error Budget:	66%
Allowable Random Error:	13.2%
Screen for Outliers?	No (N<25)

Supporting Data

Analyst:	MK
Expt. Date:	27 May 2015
Units:	ug/L
95% CI for mean:	339.18 to 353.84
2 SD Range:	315.17 to 377.85
Comment:	

Precision Data

Index	Result	Index	Result	Index	Result	Index	Result
1	332.1	6	353.1	11	328.2	16	359.6
2	353.4	7	317.4	12	343.2	17	368.9
3	350.5	8	347.4	13	339.8	18	385.5
4	350.5	9	339.9	14	353	19	331.4
5	339.9	10	330.2	15	362.9	20	343.3

O: outliers X: excluded from calculations

Accepted by: ______________________ ______________

Signature Date

Copyright 1991-2005 David G. Rhoads Associates, Inc.

EP Evaluator

Biochemistry -- University Health Network

Cyclosporine

Instrument: e411

Sample Name: BioRad 4

Simple Precision

Precision Statistics

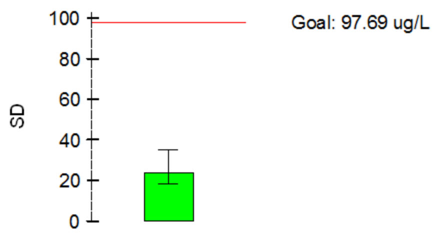

Standard Deviation (SD)	24.13
Passes?	Yes
95% Confidence for SD	18.35 to 35.24
Coefficient of Variation (CV)	3.3%
Mean	740.10 ug/L
Number of Specimens (N)	20 of 20
Number of Outliers	--

Precision Plot

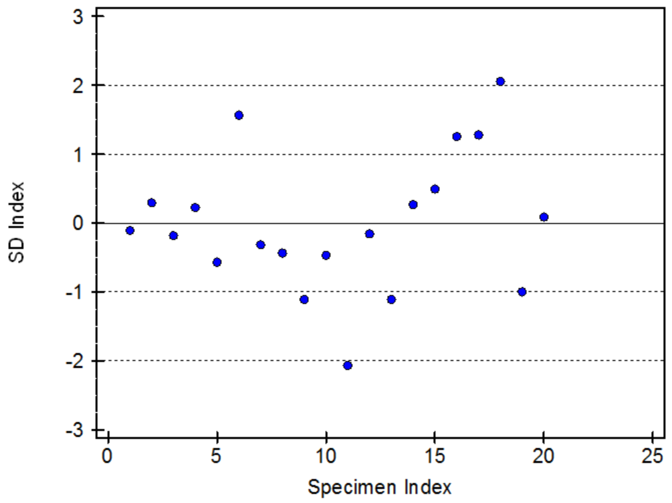

User's Specifications

Allowable Total Error:	20.0%
Random Error Budget:	66%
Allowable Random Error:	13.2%
Screen for Outliers?	No (N<25)

Supporting Data

Analyst:	MK
Expt. Date:	27 May 2015
Units:	ug/L
95% CI for mean:	728.81 to 751.39
2 SD Range:	691.85 to 788.35
Comment:	

Precision Data

Index	Result	Index	Result	Index	Result	Index	Result
1	737.5	6	777.9	11	690.2	16	770.4
2	747.2	7	732.5	12	736.3	17	771
3	735.7	8	729.6	13	713.3	18	789.7
4	745.5	9	713.3	14	746.5	19	716
5	726.4	10	728.8	15	752	20	742.2

O: outliers X: excluded from calculations

Accepted by: ______________________ ______________

Signature Date

Copyright 1991-2005 David G. Rhoads Associates, Inc.

Simple Precision

Precision Statistics

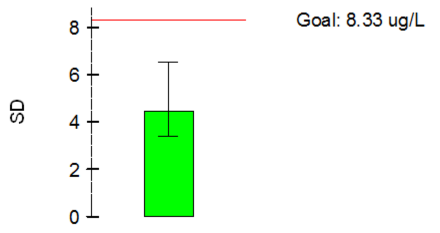

Standard Deviation (SD)	4.46
Passes?	Yes
95% Confidence for SD	3.39 to 6.51
Coefficient of Variation (CV)	7.1%
Mean	63.09 ug/L
Number of Specimens (N)	20 of 20
Number of Outliers	--

Precision Plot

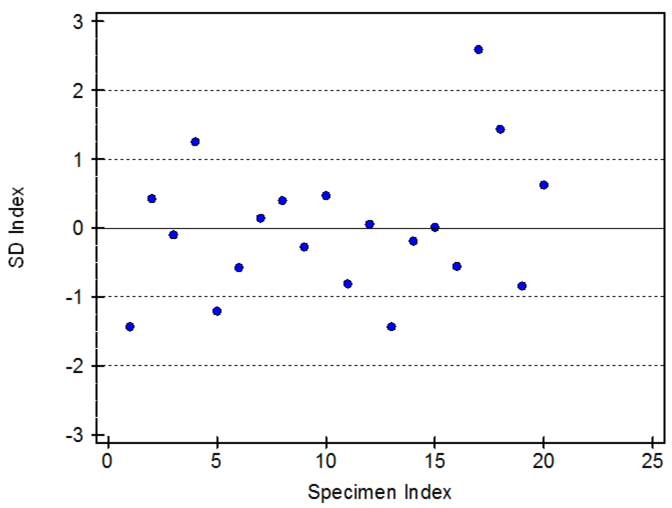

User's Specifications

Allowable Total Error:	20.0%
Random Error Budget:	66%
Allowable Random Error:	13.2%
Screen for Outliers?	No (N<25)

Supporting Data

Analyst:	MK
Expt. Date:	27 May 2015
Units:	ug/L
95% CI for mean:	61.01 to 65.18
2 SD Range:	54.17 to 72.01
Comment:	

Precision Data

Index	Result	Index	Result	Index	Result	Index	Result
1	56.7	6	60.5	11	59.5	16	60.6
2	65.0	7	63.7	12	63.3	17	74.7
3	62.7	8	64.9	13	56.7	18	69.5
4	68.7	9	61.9	14	62.3	19	59.3
5	57.7	10	65.2	15	63.1	20	65.9

O: outliers X: excluded from calculations

Accepted by:

Signature ____________________ Date ____________

Copyright 1991-2005 David G. Rhoads Associates, Inc.

EP Evaluator

Biochemistry -- University Health Network

Cyclosporine

Instrument: e411

Sample Name: ISD 2

Simple Precision

Precision Statistics

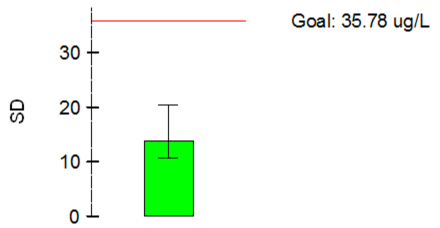

Standard Deviation (SD)	13.93
Passes?	Yes
95% Confidence for SD	10.59 to 20.34
Coefficient of Variation (CV)	5.1%
Mean	271.10 ug/L
Number of Specimens (N)	20 of 20
Number of Outliers	--

Precision Plot

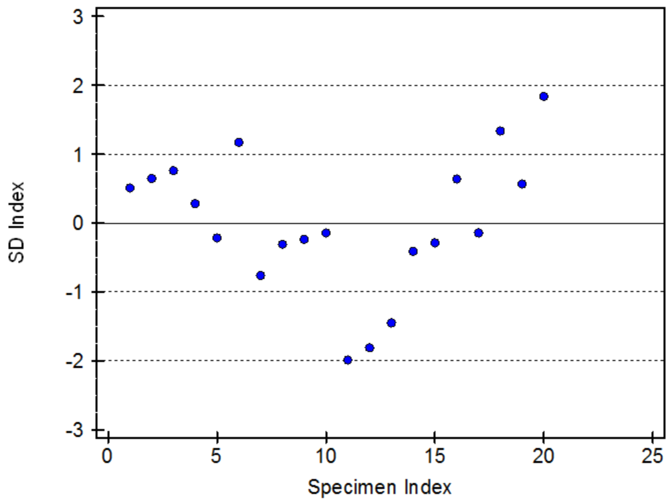

User's Specifications

Allowable Total Error:	20.0%
Random Error Budget:	66%
Allowable Random Error:	13.2%
Screen for Outliers?	No (N<25)

Supporting Data

Analyst:	MK
Expt. Date:	27 May 2015
Units:	ug/L
95% CI for mean:	264.58 to 277.61
2 SD Range:	243.24 to 298.95
Comment:	

Precision Data

Index	Result	Index	Result	Index	Result	Index	Result
1	278.2	6	287.4	11	243.4	16	280
2	280.1	7	260.5	12	245.9	17	269.1
3	281.7	8	266.8	13	250.9	18	289.7
4	275	9	267.8	14	265.4	19	279
5	268.1	10	269.1	15	267.1	20	296.7

O: outliers X: excluded from calculations

Accepted by: ______________________ ______________

Signature Date

Copyright 1991-2005 David G. Rhoads Associates, Inc.

Simple Precision

Precision Statistics

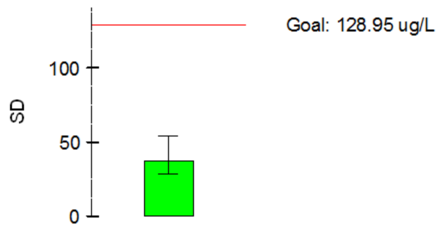

Standard Deviation (SD)	37.12
Passes?	Yes
95% Confidence for SD	28.23 to 54.21
Coefficient of Variation (CV)	3.8%
Mean	976.90 ug/L
Number of Specimens (N)	20 of 20
Number of Outliers	--

Precision Plot

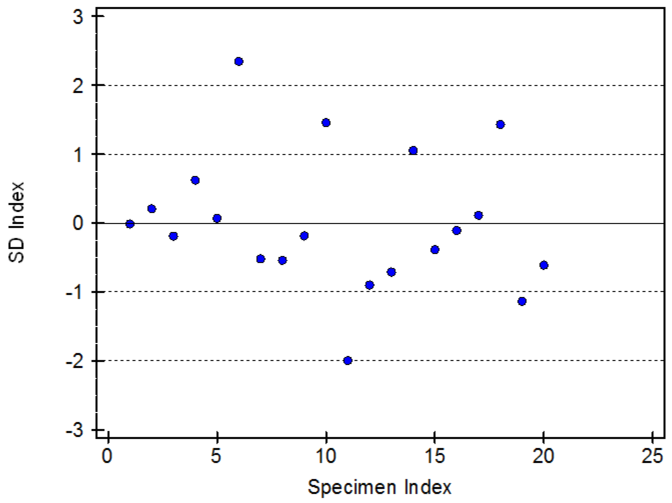

User's Specifications

Allowable Total Error:	20.0%
Random Error Budget:	66%
Allowable Random Error:	13.2%
Screen for Outliers?	No (N<25)

Supporting Data

Analyst:	MK
Expt. Date:	27 May 2015
Units:	ug/L
95% CI for mean:	959.53 to 994.27
2 SD Range:	902.67 to 1051.13
Comment:	

Precision Data

Index	Result	Index	Result	Index	Result	Index	Result
1	976.4	6	1064	11	902.8	16	972.9
2	984.6	7	957.6	12	943.5	17	981.1
3	969.8	8	956.8	13	950.5	18	1030
4	1000	9	970	14	1016	19	934.7
5	979.5	10	1031	15	962.6	20	954.2

O: outliers X: excluded from calculations

Accepted by:

Signature

Date

Copyright 1991-2005 David G. Rhoads Associates, Inc.

Tacrolimus

Instrument: e411

Sample Name: BioRad 1

Simple Precision

Precision Statistics

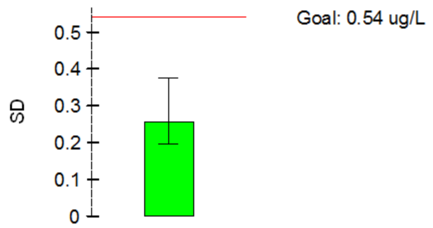

Standard Deviation (SD)	0.26
Passes?	Yes
95% Confidence for SD	0.20 to 0.38
Coefficient of Variation (CV)	6.3%
Mean	4.10 ug/L
Number of Specimens (N)	20 of 20
Number of Outliers	--

Precision Plot

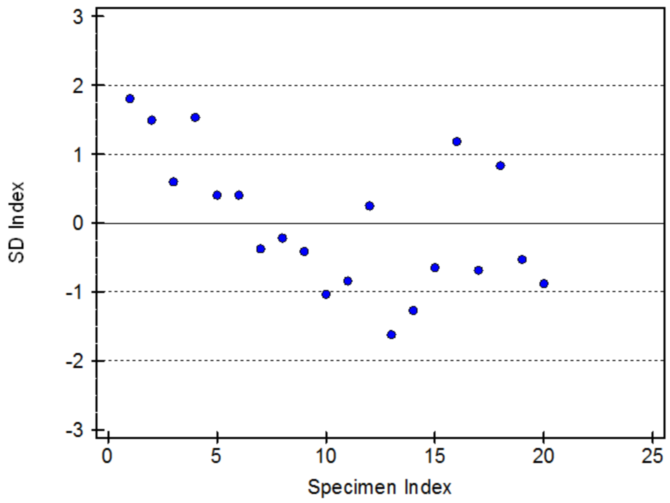

User's Specifications

Allowable Total Error:	20.0%
Random Error Budget:	66%
Allowable Random Error:	13.2%
Screen for Outliers?	No (N<25)

Supporting Data

Analyst:	MK
Expt. Date:	27 May 2015
Units:	ug/L
95% CI for mean:	3.98 to 4.22
2 SD Range:	3.58 to 4.61
Comment:	

Precision Data

Index	Result	Index	Result	Index	Result	Index	Result
1	4.6	6	4.2	11	3.9	16	4.4
2	4.5	7	4	12	4.2	17	3.9
3	4.3	8	4.0	13	3.7	18	4.3
4	4.5	9	4.0	14	3.8	19	4.0
5	4.2	10	3.8	15	3.9	20	3.9

O: outliers X: excluded from calculations

Accepted by: ______________________________ ______________

Signature Date

Copyright 1991-2005 David G. Rhoads Associates, Inc.

Simple Precision

Precision Statistics

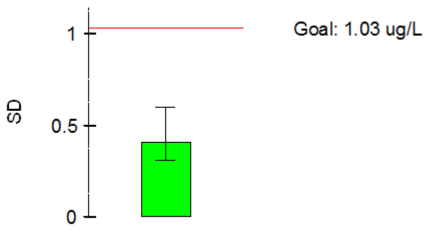

Standard Deviation (SD)	0.41
Passes?	Yes
95% Confidence for SD	0.31 to 0.60
Coefficient of Variation (CV)	5.3%
Mean	7.79 ug/L
Number of Specimens (N)	20 of 20
Number of Outliers	--

Precision Plot

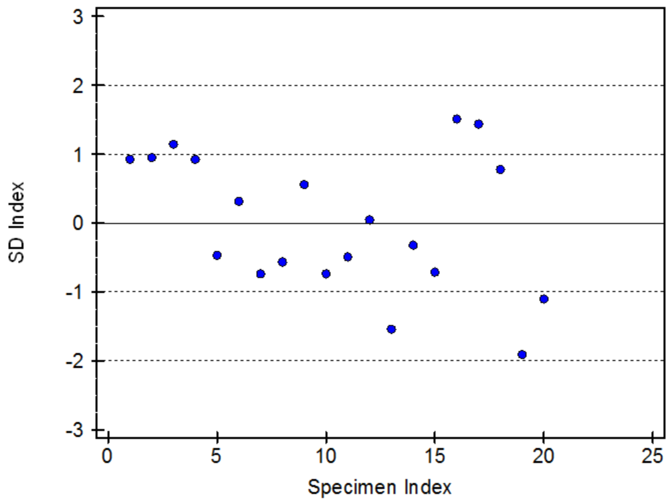

User's Specifications

Allowable Total Error:	20.0%
Random Error Budget:	66%
Allowable Random Error:	13.2%
Screen for Outliers?	No (N<25)

Supporting Data

Analyst:	MK
Expt. Date:	27 May 2015
Units:	ug/L
95% CI for mean:	7.60 to 7.98
2 SD Range:	6.97 to 8.61
Comment:	

Precision Data

Index	Result	Index	Result	Index	Result	Index	Result
1	8.2	6	7.9	11	7.6	16	8.4
2	8.2	7	7.5	12	7.8	17	8.4
3	8.3	8	7.6	13	7.2	18	8.1
4	8.2	9	8.0	14	7.7	19	7.0
5	7.6	10	7.5	15	7.5	20	7.3

O: outliers X: excluded from calculations

Accepted by: ______________________ ______________

Signature Date

Copyright 1991-2005 David G. Rhoads Associates, Inc.

Tacrolimus

Instrument: e411

Sample Name: BioRad 4

Simple Precision

Precision Statistics

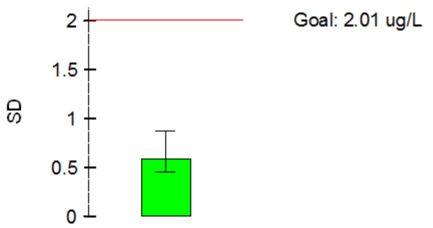

Standard Deviation (SD)	0.59
Passes?	Yes
95% Confidence for SD	0.45 to 0.87
Coefficient of Variation (CV)	3.9%
Mean	15.24 ug/L
Number of Specimens (N)	20 of 20
Number of Outliers	--

Precision Plot

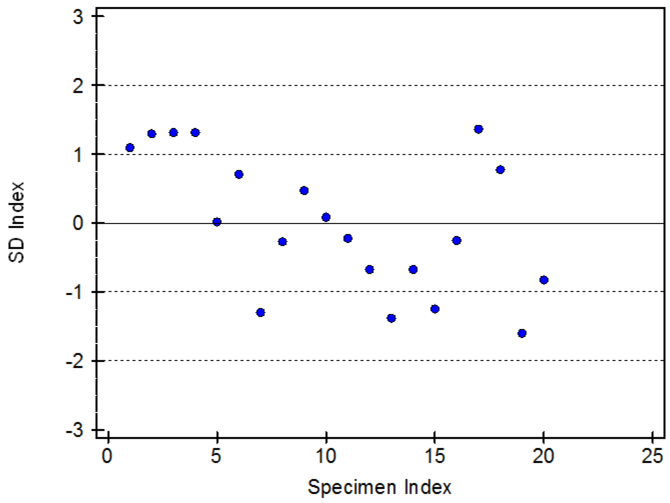

User's Specifications

Allowable Total Error:	20.0%
Random Error Budget:	66%
Allowable Random Error:	13.2%
Screen for Outliers?	No (N<25)

Supporting Data

Analyst:	MK
Expt. Date:	27 May 2015
Units:	ug/L
95% CI for mean:	14.96 to 15.52
2 SD Range:	14.05 to 16.43
Comment:	

Precision Data

Index	Result	Index	Result	Index	Result	Index	Result
1	15.9	6	15.7	11	15.1	16	15.1
2	16.0	7	14.5	12	14.8	17	16.1
3	16.0	8	15.1	13	14.4	18	15.7
4	16.0	9	15.5	14	14.8	19	14.3
5	15.3	10	15.3	15	14.5	20	14.8

O: outliers X: excluded from calculations

Accepted by:

Signature

Date

Copyright 1991-2005 David G. Rhoads Associates, Inc.

EP Evaluator

Biochemistry -- University Health Network

Tacrolimus

Instrument: e411

Sample Name: ISD 1

Simple Precision

Precision Statistics

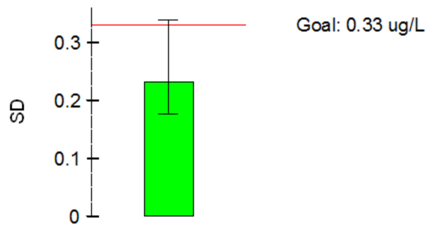

Standard Deviation (SD)	0.23
Passes?	Yes
95% Confidence for SD	0.18 to 0.34
Coefficient of Variation (CV)	9.4%
Mean	2.48 ug/L
Number of Specimens (N)	20 of 20
Number of Outliers	--

Precision Plot

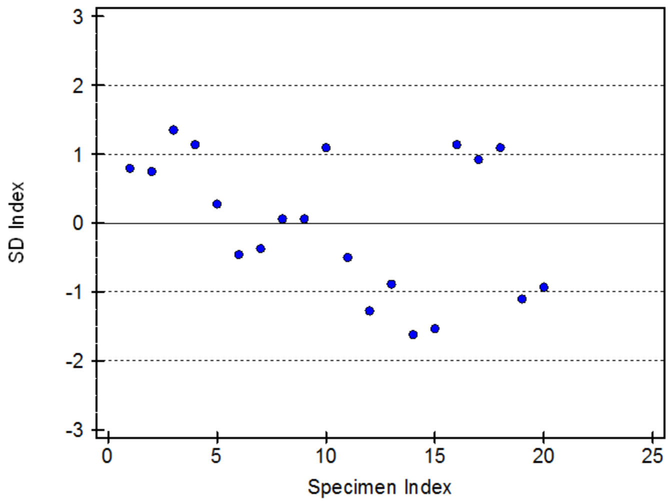

User's Specifications

Allowable Total Error:	20.0%
Random Error Budget:	66%
Allowable Random Error:	13.2%
Screen for Outliers?	No (N<25)

Supporting Data

Analyst:	MK
Expt. Date:	27 May 2015
Units:	ug/L
95% CI for mean:	2.37 to 2.58
2 SD Range:	2.01 to 2.94
Comment:	

Precision Data

Index	Result	Index	Result	Index	Result	Index	Result
1	2.7	6	2.4	11	2.4	16	2.7
2	2.6	7	2.4	12	2.2	17	2.7
3	2.8	8	2.5	13	2.3	18	2.7
4	2.7	9	2.5	14	2.1	19	2.2
5	2.5	10	2.7	15	2.1	20	2.3

O: outliers X: excluded from calculations

Accepted by: ______________________________ ______________

Signature Date

Copyright 1991-2005 David G. Rhoads Associates, Inc.

Simple Precision

Precision Statistics

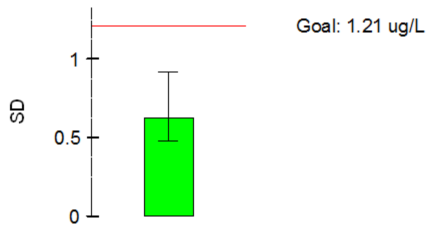

Standard Deviation (SD)	0.63
Passes?	Yes
95% Confidence for SD	0.48 to 0.92
Coefficient of Variation (CV)	6.9%
Mean	9.16 ug/L
Number of Specimens (N)	20 of 20
Number of Outliers	--

Precision Plot

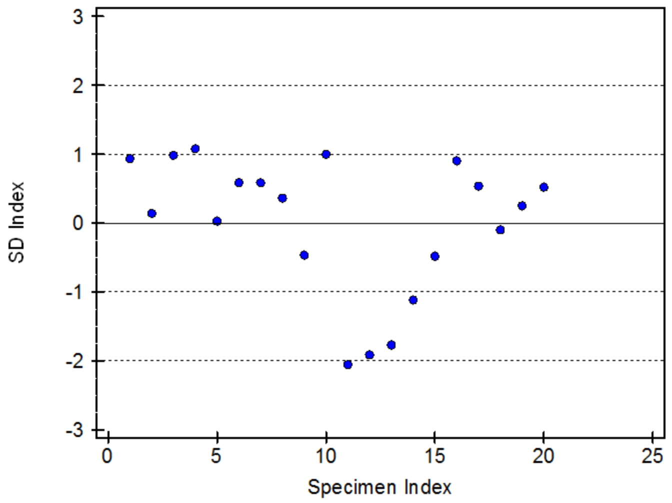

User's Specifications

Allowable Total Error:	20.0%
Random Error Budget:	66%
Allowable Random Error:	13.2%
Screen for Outliers?	No (N<25)

Supporting Data

Analyst:	MK
Expt. Date:	27 May 2015
Units:	ug/L
95% CI for mean:	8.87 to 9.46
2 SD Range:	7.91 to 10.42
Comment:	

Precision Data

Index	Result	Index	Result	Index	Result	Index	Result
1	9.8	6	9.5	11	7.9	16	9.7
2	9.3	7	9.5	12	8.0	17	9.5
3	9.8	8	9.4	13	8.1	18	9.1
4	9.8	9	8.9	14	8.5	19	9.3
5	9.2	10	9.8	15	8.9	20	9.5

O: outliers X: excluded from calculations

Accepted by: ______________________________ ______________

Signature Date

Copyright 1991-2005 David G. Rhoads Associates, Inc.

Tacrolimus

Instrument: e411

Sample Name: ISD 3

Simple Precision

Precision Statistics

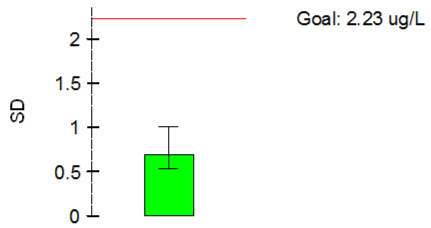

Standard Deviation (SD)	0.69
Passes?	Yes
95% Confidence for SD	0.53 to 1.01
Coefficient of Variation (CV)	4.1%
Mean	16.88 ug/L
Number of Specimens (N)	20 of 20
Number of Outliers	--

Precision Plot

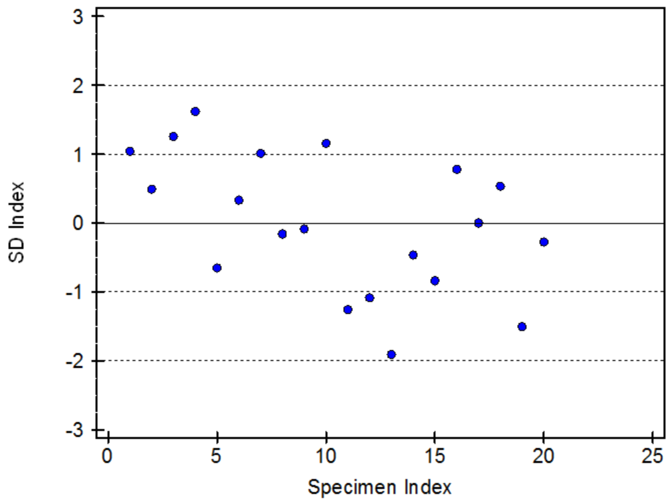

User's Specifications

Allowable Total Error:	20.0%
Random Error Budget:	66%
Allowable Random Error:	13.2%
Screen for Outliers?	No (N<25)

Supporting Data

Analyst:	MK
Expt. Date:	27 May 2015
Units:	ug/L
95% CI for mean:	16.56 to 17.20
2 SD Range:	15.50 to 18.26
Comment:	

Precision Data

Index	Result	Index	Result	Index	Result	Index	Result
1	17.6	6	17.1	11	16.0	16	17.4
2	17.2	7	17.6	12	16.1	17	16.9
3	17.8	8	16.8	13	15.6	18	17.3
4	18	9	16.8	14	16.6	19	15.8
5	16.4	10	17.7	15	16.3	20	16.7

O: outliers X: excluded from calculations

Accepted by:

Signature Date

Copyright 1991-2005 David G. Rhoads Associates, Inc.

Simple Precision

Report Interpretation Guide

CLIA and CAP require periodic verification of Precision. The Simple Precision experiment is a quick and dirty way to fulfill the letter of this requirement. The procedure is to make repeated measurements of a single sample, and compute simple statistics like mean, SD, and CV. EP Evaluator offers two additional options:

- Enter a precision goal (Allowable Random Error), and the program will report that the test "passes" if the calculated SD does not exceed Allowable Random Error.
- Automatically identify and remove outliers. (Requires a minimum of 25 results.)

Definitions

Precision: Ability to obtain the same result upon repeated measurement of a specimen. Not necessarily the *correct* result, just the *same* result. (Ability to get the correct result is *Accuracy.)*

Mean: Average value, computed by adding the results and dividing the total by the number of results.

Standard Deviation (SD): The primary measure of dispersion or variation of the individual results about the mean. In other words, a measure of Precision. For many analytes, SD varies with sample concentration. Example: For Glucose, an SD of 10 for a 400 mg/dL sample represents very good precision. For a 40 mg/dL sample, it is very poor precision.

Coefficient of Variation (CV): SD expressed as a percent of the mean. For analytes where error varies with concentration, CV is more likely to remain constant across the analytical range. Example: 2.5% CV for Glucose is very good precision at any concentration.

Within-Run and Total SD: Manufacturers often publish these statistics in package inserts. They are computed from a more complex precision experiment that requires replicate measurements over several days. Total SD from this complex precision experiment is NOT comparable to EP Evaluator's Simple Precision SD.

Number of Specimens (N): A good precision study should include 20-50 replicates. Mathematically, it is possible to calculate SD and CV from only 3 replicates, but it is not a very reliable estimate. For example, suppose the true SD is 1.00. An estimate based on 3 replicates might easily be as low as 0.52 or as high as 6.29 (95% confidence). At N=20, the 95% confidence interval is much narrower: 0.76 to 1.46. At N=50 it narrows further: 0.84 to 1.24.

95% Confidence Interval for SD: In a sense, this measures the "precision of the precision". It show how much the SD might vary if the precision experiment was repeated with different experimental results. The width of the confidence interval depends on two factors: N and the intrinsic SD of the system. The reliability of the precision estimate improves as N increases.

Outlier: A result so far from the others as to arouse suspicion that it was generated by a different mechanism. Some common causes: typing a number with the decimal point in the wrong place, analyzing the wrong sample, or entering incorrect specimen identification.

An outlier can have a drastic effect on the calculated mean and SD. For example:

- Start with 10 results, five 1's and five 2's. The mean is 1.5 and the SD is 0.53
- Change the first 1 to 10. The SD increases from 0.53 to 2.72. The mean changes from 1.5 to 2.4

The best way to deal with an outlier is to (manually) determine its cause and correct it. Another option is to use a statistical procedure, like the one described below, to remove outliers automatically.

Outlier Identification

EP Evaluator identifies outliers using Tukey's method, a nonparametric procedure that is (relatively) unaffected by the outlier it is trying to detect:

- Arrange the results in increasing order, and determine the 25th and 75th percentiles (P25 and P75).
- Determine the Interquartile Range (IQR), IQR = P75 - P25.
- Points greater than P75 + 3 IQR or lower than P25 - 3 IQR are considered outliers. This is roughly comparable to mean +/- 5 SD.

Outliers are excluded from precision calculations, and flagged on the Results Listing. If more than 5% of the results are outliers, the report is stamped PRELIMINARY.

Allowable Total Error (TEa), and the Error Budget

TEa states the laboratory's policy for how much error is medically (or administratively) acceptable. Regulatory requirements represent an upper limit. Example: the CLIA limit for Sodium is 4 mmol/L.

Total Error has two major components: **Systematic Error** (synonym Bias) and **Random Error** (synonym Imprecision). The **Error Budget** allocates a fraction of the Allowable Total Error for Systematic Error, and another fraction for Random Error. Establishing an appropriate Error Budget allows the

Copyright 1991-2005 David G. Rhoads Associates, Inc.

Simple Precision

Report Interpretation Guide

lab to control accuracy and precision separately, with reasonable confidence that Total Error will also remain in control. Recommended ranges of values are 25-50% for the Systematic Error Budget and 16-25% for the Random Error Budget.

Pass or Fail?

The objective is to determine whether precision is acceptable, so the standard of comparison is Allowable Random Error. The test passes if the computed SD does not exceed Allowable Random Error.

Example: Suppose Allowable Total Error for Sodium is 4 mmol/L, with a Random Error Budget of 25%. Allowable Random Error is 25% x 4 or 1 mmol/L. The precision test passes when the computed SD is at or below 1 mmol/L.

Preliminary Report

The word PRELIMINARY printed diagonally across the report indicates that the data is incomplete, and the report is not acceptable as a final report. Some or all of the statistics may be missing. Causes:

- Less than 3 unexcluded results.
- More than 5% of the results are outliers.

Chart Interpretation

Levey-Jennings Chart: A scatter plot of Standard Deviation Index (SDI) on the Y-axis vs. specimen index on the X-axis. Specimen index reflects the order in which the results were typed into the program. SDI = (Result - mean) / SD.

Precision Goal: This chart appears only when Allowable Random Error is provided. It diagrams the SD and its 95% confidence interval in relation to allowable random error (the goal). The wide bar represents the SD. The fence marks above and below the SD bar show the 95% confidence limits.

- The report calls the test a "pass" if the SD (top of the wide bar) does not exceed the goal. The bar is green if the test passes, or red if it fails.
- If you consider all points within the 95% confidence interval to be statistically identical, you might consider the test at least marginally acceptable if the lower confidence limit does not exceed the goal.
- The ideal situation is when the upper 95% confidence limit does not exceed the goal.

Copyright 1991-2005 David G. Rhoads Associates, Inc.

EP Evaluator

Biochemistry -- University Health Network

Simple Precision Summary

Instrument	Analyte	Sample	N	Mean	SD	CV
Roche	✔ Sirolimus	Bio-Rad L1	20 of 20	5.939	0.279	4.7%
	✔ Sirolimus	Bio-Rad L3	20 of 20	9.489	0.541	5.7%
	✔ Sirolimus	Bio-Rad L4	20 of 20	14.244	1.170	8.2%
	✔ Sirolimus	ISD L1	20 of 20	3.423	0.156	4.6%
	✔ Sirolimus	ISD L2	20 of 20	8.700	0.486	5.6%
	✔ Sirolimus	ISD L3	20 of 20	15.356	0.724	4.7%

⟐ Required parameters are missing.

✘ Experiment 'fails' (or not enough results).

✔ Experiment 'passes' (or adequate number of results).

Copyright 1991-2005 David G. Rhoads Associates, Inc.

EP Evaluator

Biochemistry -- University Health Network

Sirolimus

Instrument: Roche

Sample Name: Bio-Rad L1

Simple Precision

Precision Statistics

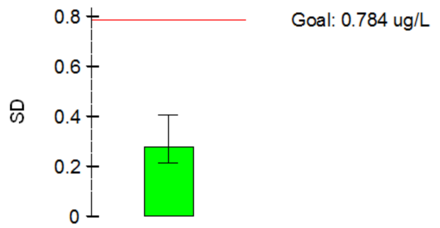

Standard Deviation (SD)	0.279
Passes?	Yes
95% Confidence for SD	0.212 to 0.407
Coefficient of Variation (CV)	4.7%
Mean	5.939 ug/L
Number of Specimens (N)	20 of 20
Number of Outliers	--

Precision Plot

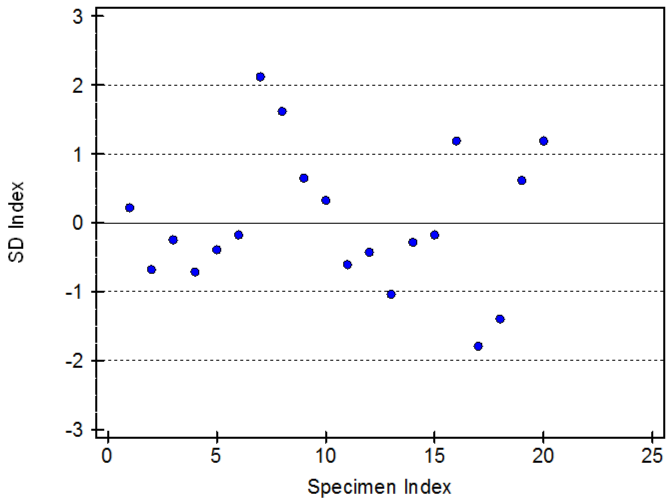

User's Specifications

Allowable Total Error:	20.0%
Random Error Budget:	66%
Allowable Random Error:	13.2%
Screen for Outliers?	No

Supporting Data

Analyst:	AWF
Expt. Date:	04 Jul 2016
Units:	ug/L
95% CI for mean:	5.809 to 6.069
2 SD Range:	5.382 to 6.496
Comment:	Claim = 5.2 ug/L

Precision Data

Index	Result	Index	Result	Index	Result	Index	Result
1	6.00	6	5.89	11	5.77	16	6.27
2	5.75	7	6.53	12	5.82	17	5.44
3	5.87	8	6.39	13	5.65	18	5.55
4	5.74	9	6.12	14	5.86	19	6.11
5	5.83	10	6.03	15	5.89	20	6.27

O: outliers X: excluded from calculations

Accepted by: ______________________________ ______________

Signature Date

Copyright 1991-2005 David G. Rhoads Associates, Inc.

Simple Precision

Precision Statistics

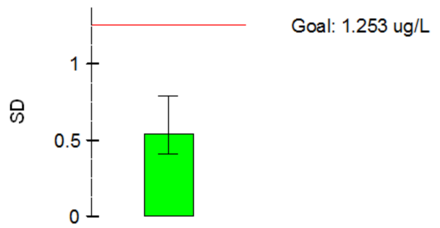

Standard Deviation (SD)	0.541
Passes?	Yes
95% Confidence for SD	0.411 to 0.790
Coefficient of Variation (CV)	5.7%
Mean	9.489 ug/L
Number of Specimens (N)	20 of 20
Number of Outliers	--

Precision Plot

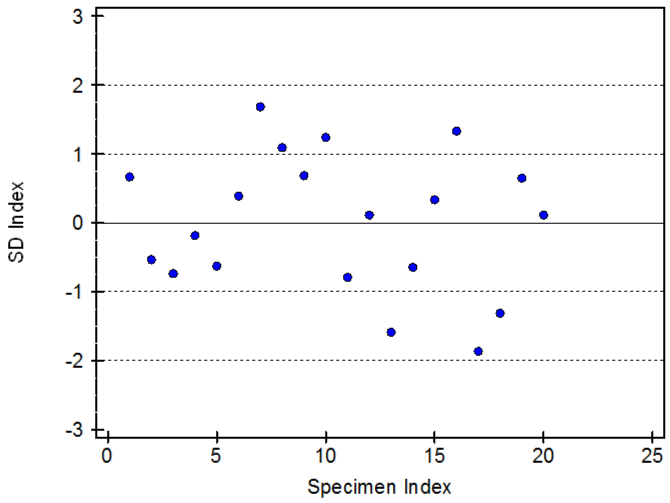

User's Specifications

Allowable Total Error:	20.0%
Random Error Budget:	66%
Allowable Random Error:	13.2%
Screen for Outliers?	No

Supporting Data

Analyst:	AWF
Expt. Date:	04 Jul 2016
Units:	ug/L
95% CI for mean:	9.236 to 9.743
2 SD Range:	8.408 to 10.571
Comment:	Claim = 9.77 ug/L

Precision Data

Index	Result	Index	Result	Index	Result	Index	Result
1	9.85	6	9.70	11	9.06	16	10.21
2	9.20	7	10.40	12	9.55	17	8.48
3	9.09	8	10.08	13	8.63	18	8.78
4	9.39	9	9.86	14	9.14	19	9.84
5	9.15	10	10.16	15	9.67	20	9.55

O: outliers X: excluded from calculations

Accepted by: ______________________ ______________

Signature Date

Copyright 1991-2005 David G. Rhoads Associates, Inc.

EP Evaluator

Biochemistry -- University Health Network

Sirolimus

Instrument: Roche

Sample Name: Bio-Rad L4

Simple Precision

Precision Statistics

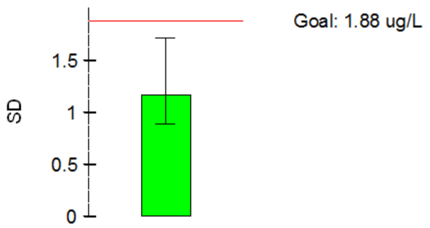

Standard Deviation (SD)	1.170
Passes?	Yes
95% Confidence for SD	0.890 to 1.709
Coefficient of Variation (CV)	8.2%
Mean	14.244 ug/L
Number of Specimens (N)	20 of 20
Number of Outliers	--

Precision Plot

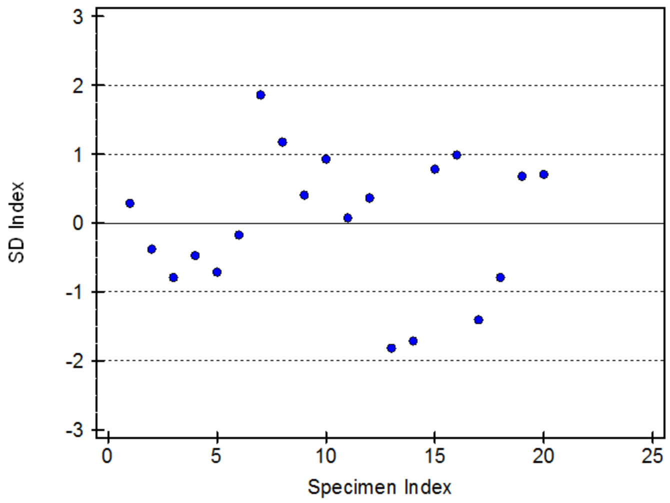

User's Specifications

Allowable Total Error:	20.0%
Random Error Budget:	66%
Allowable Random Error:	13.2%
Screen for Outliers?	No

Supporting Data

Analyst:	AWF
Expt. Date:	04 Jul 2016
Units:	ug/L
95% CI for mean:	13.696 to 14.792
2 SD Range:	11.904 to 16.584
Comment:	Claim = 15.6 ug/L

Precision Data

Index	Result	Index	Result	Index	Result	Index	Result
1	14.58	6	14.04	11	14.33	16	15.40
2	13.80	7	16.42	12	14.67	17	12.60
3	13.32	8	15.62	13	12.12	18	13.32
4	13.69	9	14.72	14	12.24	19	15.04
5	13.41	10	15.33	15	15.16	20	15.07

O: outliers X: excluded from calculations

Accepted by: ______________________ ______________

Signature Date

Copyright 1991-2005 David G. Rhoads Associates, Inc.

EP Evaluator

Biochemistry -- University Health Network

Sirolimus

Instrument: Roche

Sample Name: ISD L1

Simple Precision

Precision Statistics

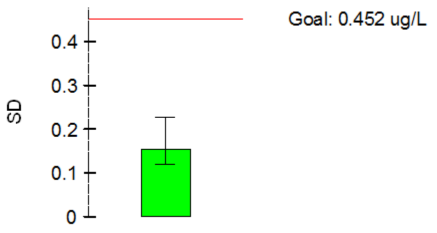

Standard Deviation (SD)	0.156
Passes?	Yes
95% Confidence for SD	0.118 to 0.228
Coefficient of Variation (CV)	4.6%
Mean	3.423 ug/L
Number of Specimens (N)	20 of 20
Number of Outliers	--

Precision Plot

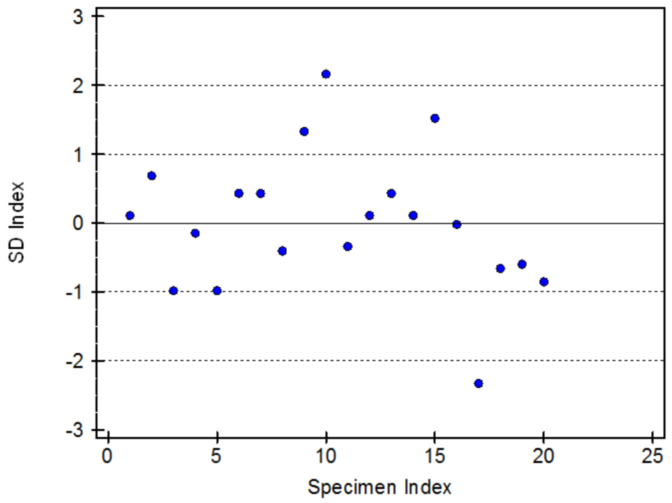

User's Specifications

Allowable Total Error:	20.0%
Random Error Budget:	66%
Allowable Random Error:	13.2%
Screen for Outliers?	No

Supporting Data

Analyst:	AWF
Expt. Date:	04 Jul 2016
Units:	ug/L
95% CI for mean:	3.350 to 3.496
2 SD Range:	3.111 to 3.735
Comment:	Claim = 3.46 ug/L

Precision Data

Index	Result	Index	Result	Index	Result	Index	Result
1	3.44	6	3.49	11	3.37	16	3.42
2	3.53	7	3.49	12	3.44	17	3.06
3	3.27	8	3.36	13	3.49	18	3.32
4	3.40	9	3.63	14	3.44	19	3.33
5	3.27	10	3.76	15	3.66	20	3.29

O: outliers X: excluded from calculations

Accepted by:

Signature

Date

Copyright 1991-2005 David G. Rhoads Associates, Inc.

Simple Precision

Precision Statistics

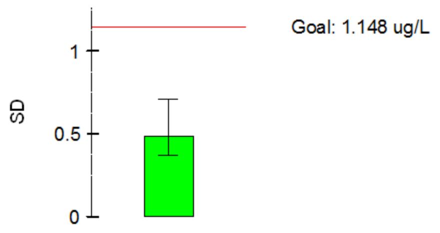

Standard Deviation (SD)	0.486
Passes?	Yes
95% Confidence for SD	0.369 to 0.709
Coefficient of Variation (CV)	5.6%
Mean	8.700 ug/L
Number of Specimens (N)	20 of 20
Number of Outliers	--

Precision Plot

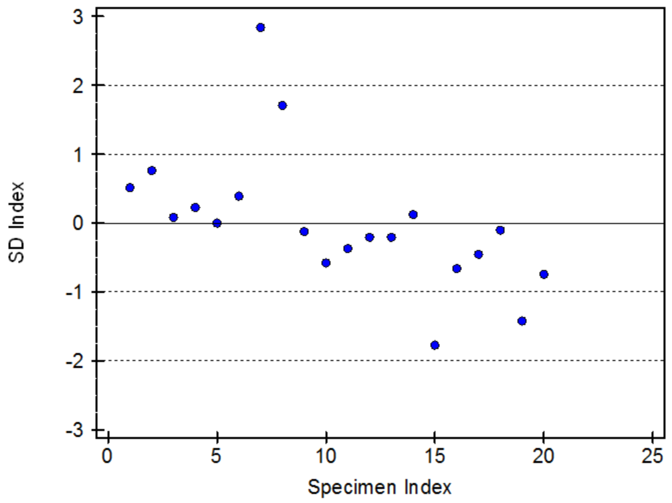

User's Specifications

Allowable Total Error:	20.0%
Random Error Budget:	66%
Allowable Random Error:	13.2%
Screen for Outliers?	No

Supporting Data

Analyst:	AWF
Expt. Date:	04 Jul 2016
Units:	ug/L
95% CI for mean:	8.473 to 8.928
2 SD Range:	7.729 to 9.672
Comment:	Claim = 9.39 ug/L

Precision Data

Index	Result	Index	Result	Index	Result	Index	Result
1	8.95	6	8.89	11	8.52	16	8.38
2	9.07	7	10.08	12	8.60	17	8.48
3	8.74	8	9.53	13	8.60	18	8.65
4	8.81	9	8.64	14	8.76	19	8.01
5	8.70	10	8.42	15	7.84	20	8.34

O: outliers X: excluded from calculations

Accepted by:

Signature _______________ Date _______________

Copyright 1991-2005 David G. Rhoads Associates, Inc.

Simple Precision

Precision Statistics

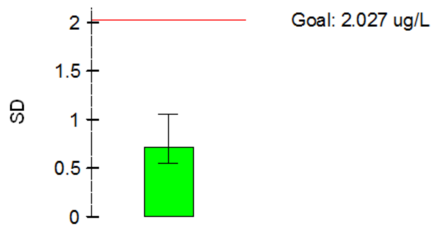

Standard Deviation (SD)	0.724
Passes?	Yes
95% Confidence for SD	0.550 to 1.057
Coefficient of Variation (CV)	4.7%
Mean	15.356 ug/L
Number of Specimens (N)	20 of 20
Number of Outliers	--

Precision Plot

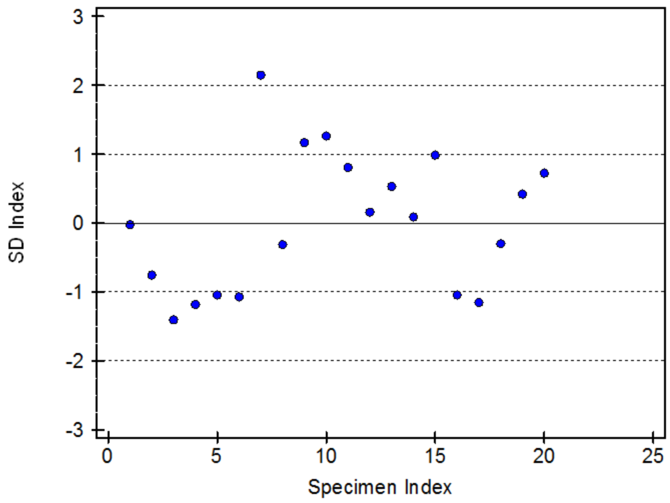

User's Specifications

Allowable Total Error:	20.0%
Random Error Budget:	66%
Allowable Random Error:	13.2%
Screen for Outliers?	No

Supporting Data

Analyst:	AWF
Expt. Date:	04 Jul 2016
Units:	ug/L
95% CI for mean:	15.017 to 15.695
2 SD Range:	13.909 to 16.803
Comment:	Claim = 15.6 ug/L

Precision Data

Index	Result	Index	Result	Index	Result	Index	Result
1	15.34	6	14.58	11	15.94	16	14.60
2	14.81	7	16.91	12	15.47	17	14.52
3	14.34	8	15.13	13	15.74	18	15.14
4	14.50	9	16.20	14	15.42	19	15.66
5	14.60	10	16.27	15	16.07	20	15.88

O: outliers X: excluded from calculations

Accepted by: ______________________ ______________

Signature Date

Copyright 1991-2005 David G. Rhoads Associates, Inc.

Simple Precision

Report Interpretation Guide

CLIA and CAP require periodic verification of Precision. The Simple Precision experiment is a quick and dirty way to fulfill the letter of this requirement. The procedure is to make repeated measurements of a single sample, and compute simple statistics like mean, SD, and CV. EP Evaluator offers two additional options:

- Enter a precision goal (Allowable Random Error), and the program will report that the test "passes" if the calculated SD does not exceed Allowable Random Error.
- Automatically identify and remove outliers. (Requires a minimum of 25 results.)

Definitions

Precision: Ability to obtain the same result upon repeated measurement of a specimen. Not necessarily the *correct* result, just the *same* result. (Ability to get the correct result is *Accuracy.)*

Mean: Average value, computed by adding the results and dividing the total by the number of results.

Standard Deviation (SD): The primary measure of dispersion or variation of the individual results about the mean. In other words, a measure of Precision. For many analytes, SD varies with sample concentration. Example: For Glucose, an SD of 10 for a 400 mg/dL sample represents very good precision. For a 40 mg/dL sample, it is very poor precision.

Coefficient of Variation (CV): SD expressed as a percent of the mean. For analytes where error varies with concentration, CV is more likely to remain constant across the analytical range. Example: 2.5% CV for Glucose is very good precision at any concentration.

Within-Run and Total SD: Manufacturers often publish these statistics in package inserts. They are computed from a more complex precision experiment that requires replicate measurements over several days. Total SD from this complex precision experiment is NOT comparable to EP Evaluator's Simple Precision SD.

Number of Specimens (N): A good precision study should include 20-50 replicates. Mathematically, it is possible to calculate SD and CV from only 3 replicates, but it is not a very reliable estimate. For example, suppose the true SD is 1.00. An estimate based on 3 replicates might easily be as low as 0.52 or as high as 6.29 (95% confidence). At N=20, the 95% confidence interval is much narrower: 0.76 to 1.46. At N=50 it narrows further: 0.84 to 1.24.

95% Confidence Interval for SD: In a sense, this measures the "precision of the precision". It show how much the SD might vary if the precision experiment was repeated with different experimental results. The width of the confidence interval depends on two factors: N and the intrinsic SD of the system. The reliability of the precision estimate improves as N increases.

Outlier: A result so far from the others as to arouse suspicion that it was generated by a different mechanism. Some common causes: typing a number with the decimal point in the wrong place, analyzing the wrong sample, or entering incorrect specimen identification.

An outlier can have a drastic effect on the calculated mean and SD. For example:

- Start with 10 results, five 1's and five 2's. The mean is 1.5 and the SD is 0.53
- Change the first 1 to 10. The SD increases from 0.53 to 2.72. The mean changes from 1.5 to 2.4

The best way to deal with an outlier is to (manually) determine its cause and correct it. Another option is to use a statistical procedure, like the one described below, to remove outliers automatically.

Outlier Identification

EP Evaluator identifies outliers using Tukey's method, a nonparametric procedure that is (relatively) unaffected by the outlier it is trying to detect:

- Arrange the results in increasing order, and determine the 25th and 75th percentiles (P25 and P75).
- Determine the Interquartile Range (IQR), IQR = P75 - P25.
- Points greater than P75 + 3 IQR or lower than P25 - 3 IQR are considered outliers. This is roughly comparable to mean +/- 5 SD.

Outliers are excluded from precision calculations, and flagged on the Results Listing. If more than 5% of the results are outliers, the report is stamped PRELIMINARY.

Allowable Total Error (TEa), and the Error Budget

TEa states the laboratory's policy for how much error is medically (or administratively) acceptable. Regulatory requirements represent an upper limit. Example: the CLIA limit for Sodium is 4 mmol/L.

Total Error has two major components: **Systematic Error** (synonym Bias) and **Random Error** (synonym Imprecision). The **Error Budget** allocates a fraction of the Allowable Total Error for Systematic Error, and another fraction for Random Error. Establishing an appropriate Error Budget allows the

Copyright 1991-2005 David G. Rhoads Associates, Inc.

Simple Precision
Report Interpretation Guide

lab to control accuracy and precision separately, with reasonable confidence that Total Error will also remain in control. Recommended ranges of values are 25-50% for the Systematic Error Budget and 16-25% for the Random Error Budget.

Pass or Fail?

The objective is to determine whether precision is acceptable, so the standard of comparison is Allowable Random Error. The test passes if the computed SD does not exceed Allowable Random Error.

Example: Suppose Allowable Total Error for Sodium is 4 mmol/L, with a Random Error Budget of 25%. Allowable Random Error is 25% x 4 or 1 mmol/L. The precision test passes when the computed SD is at or below 1 mmol/L.

Preliminary Report

The word PRELIMINARY printed diagonally across the report indicates that the data is incomplete, and the report is not acceptable as a final report. Some or all of the statistics may be missing. Causes:

- Less than 3 unexcluded results.
- More than 5% of the results are outliers.

Chart Interpretation

Levey-Jennings Chart: A scatter plot of Standard Deviation Index (SDI) on the Y-axis vs. specimen index on the X-axis. Specimen index reflects the order in which the results were typed into the program. SDI = (Result - mean) / SD.

Precision Goal: This chart appears only when Allowable Random Error is provided. It diagrams the SD and its 95% confidence interval in relation to allowable random error (the goal). The wide bar represents the SD. The fence marks above and below the SD bar show the 95% confidence limits.

- The report calls the test a "pass" if the SD (top of the wide bar) does not exceed the goal. The bar is green if the test passes, or red if it fails.
- If you consider all points within the 95% confidence interval to be statistically identical, you might consider the test at least marginally acceptable if the lower confidence limit does not exceed the goal.
- The ideal situation is when the upper 95% confidence limit does not exceed the goal.

Copyright 1991-2005 David G. Rhoads Associates, Inc.

Linearity Summary

Instrument		Analyte	Linearity	Accuracy	Reportable Range	Precision
e411	✔	Cyclosporine	Pass	--	--	--
	✔	Tacrolimus	Pass	--	--	--

◈ Required parameters are missing.

✘ Experiment 'fails' (or not enough results).

✔ Experiment 'passes' (or adequate number of results).

Copyright 1991-2005 David G. Rhoads Associates, Inc.

Linearity

	Assigned	N	Est	Mean	Residual	Linearity
LN31-8	48.045	2	47.87	47.57	-0.30	Pass
LN31-9	96.09	0	--	--	--	--
LN31-10	192.18	2	187.78	193.20	5.42	Pass
LN31-11	336.315	2	327.69	336.40	8.71	Pass
LN31-12	480.45	2	467.59	468.45	0.86	Pass
LN31-13	720.675	2	700.77	681.65	-19.12	Pass
LN31-14	960.9	2	933.95	960.90	26.95	Pass

See User's Specifications for Pass/Fail criteria

Linearity Summary

	Overall
Slope	0.971
Intercept	1.24
Obs. Err.	2.8%
N	6

LINEAR within Allowable Systematic Error of 5.0%

Experimental Results

LN31-8	42.94	52.2
LN31-9	X79.62	
LN31-10	192	194.4
LN31-11	337.8	335
LN31-12	465.3	471.6
LN31-13	683.6	679.7
LN31-14	985.4	936.4

X: Excluded from calculations

User's Specifications

Allowable Total Error:	15.0%
Systematic Error Budget:	33%
Allowable Systematic Error:	5.0%

Supporting Data

Analyst:	MK
Date:	27 May 2015
Value Mode:	Preassigned
Units:	ug/L
Lot Number:	lot
Comment:	

Analytical Claim

The Linearity of Cyclosporine was analyzed on e411 over a measured range of 47.57 to 960.90 ug/L. Allowable systematic error (SEa) was 5.0%. The results are LINEAR.

Accepted by: ______________________________ ______________

Signature Date

Copyright 1991-2005 David G. Rhoads Associates, Inc.

Linearity

Scatter Plot

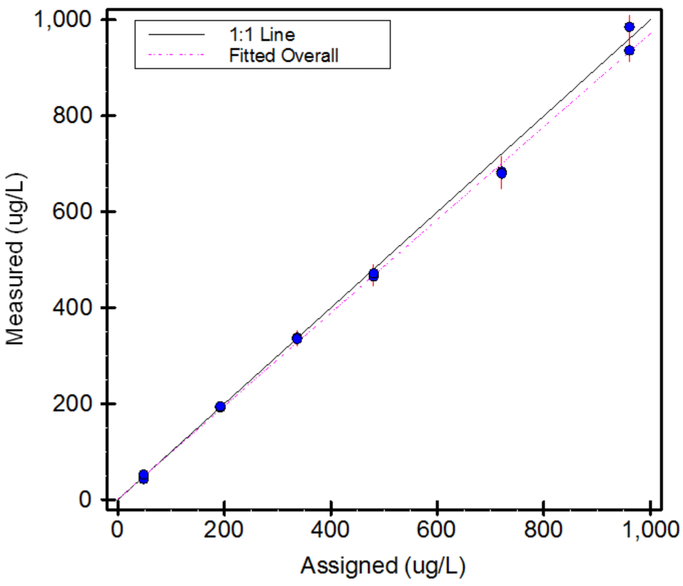

Residual Plot

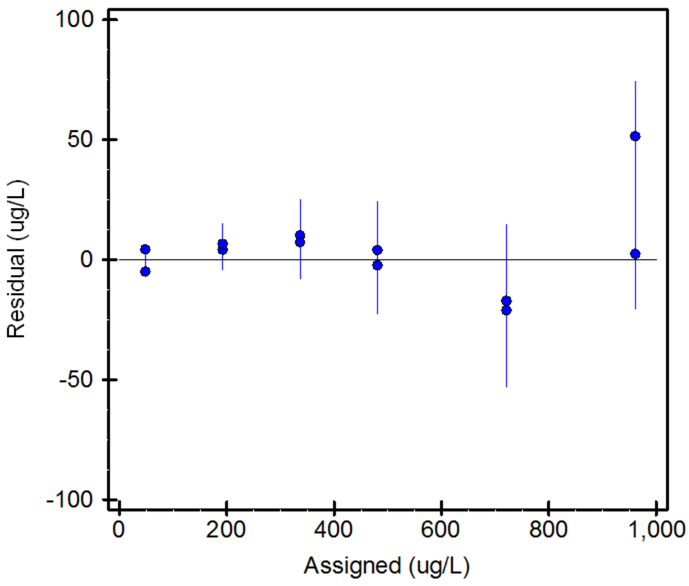

Copyright 1991-2005 David G. Rhoads Associates, Inc.

Linearity

	Assigned	N	Est	Mean	Residual	Linearity
LN31-8	1.3558	1	1.31	1.26	-0.05	Pass
LN31-9	2.7115	0	--	--	--	--
LN31-10	5.423	2	5.55	5.75	0.20	Pass
LN31-11	9.4902	2	9.79	9.93	0.13	Pass
LN31-12	13.5575	2	14.03	14.60	0.57	Pass
LN31-13	20.3362	2	21.10	21.08	-0.03	Pass
LN31-14	27.115	2	28.17	27.12	-1.05	Pass

See User's Specifications for Pass/Fail criteria

Linearity Summary

	Overall
Slope	1.043
Intercept	-0.10
Obs. Err.	3.9%
N	6

LINEAR within Allowable Systematic Error of 5.0%

Experimental Results

LN31-8	1.26	
LN31-9	X2.44	
LN31-10	5.62	5.88
LN31-11	10.03	9.82
LN31-12	14.22	14.98
LN31-13	21.1	21.05
LN31-14	26.71	27.52

X: Excluded from calculations

User's Specifications

Allowable Total Error:	15.0%
Systematic Error Budget:	33%
Allowable Systematic Error:	5.0%

Supporting Data

Analyst:	MK
Date:	27 May 2015
Value Mode:	Preassigned
Units:	ug/L
Lot Number:	lot
Comment:	

Analytical Claim

The Linearity of Tacrolimus was analyzed on e411 over a measured range of 1.26 to 27.12 ug/L. Allowable systematic error (SEa) was 5.0%. The results are LINEAR.

Accepted by: ______________________________ ______________

Signature Date

Copyright 1991-2005 David G. Rhoads Associates, Inc.

Linearity

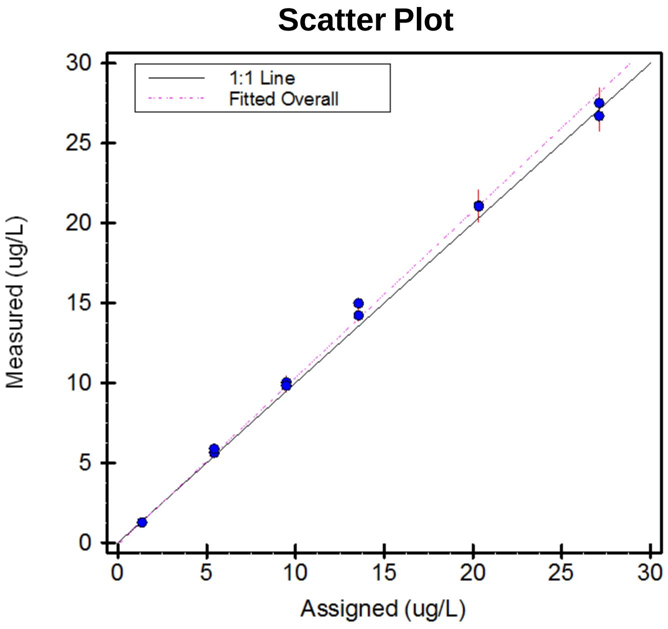

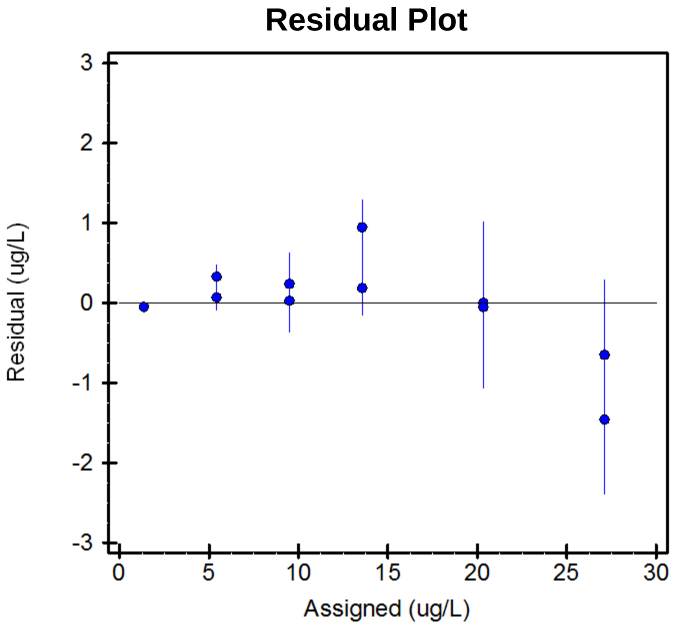

Copyright 1991-2005 David G. Rhoads Associates, Inc.

Linearity

Report Interpretation Guide

For EP Evaluator purposes, Linearity experiments are experiments that use specimens with defined concentrations. This includes Calibration Verification, Accuracy, and Reportable Range, as well as Linearity. The Linearity module can also verify Precision. This means you can verify three of the four CLIA '88 requirements with a single experiment.

User-selectable options determine which of these parameters the report verifies. Also, the user may request Pass/Fail flags against a specific allowable error criterion, or he/she may simply report selected statistical measures.

Experiment Procedure: Replicate measurements are made on 3-11 specimens, with (known) concentrations spread across the reportable range. Ideally, the lowest and highest specimens should challenge the limits of the range.

Accuracy (or Recovery)

Definition: The ability to recover the correct amount of analyte present in the specimen.

Verification process: Accuracy can be verified only when the "correct" amount of analyte (the **Assigned Value**) is known. While it is possible to determine recovery using a single replicate, one gets a more reliable estimate when 2 to 4 replicates are assayed.

Key statistic: Recovery = 100 x Measured Mean / Assigned Value

Reportable Range

Definition: As used in CLIA, this term refers to the Analytical Range or Assay Range -- the maximum range of values that can be assayed accurately without dilution. The CAP term "Analytical Measurement Range" (AMR) is a synonym for Reportable Range.

Verification Procedure: Reportable Range is verified if two conditions are met: 1) the assigned values of the lowest and highest specimens are within proximity limits of the Reportable Range limits, and 2) these two specimens are acceptably accurate.

Proximity Limits define how close the lowest and highest specimens must be to the Reportable Range limits.

Calibration Verification

verifies whether a method is properly calibrated. Calibration Verification is identical to verifying both Accuracy and Reportable Range. The only difference is that the report is titled to match the regulatory requirement.

CLIA requires a minimum of three specimens, each assayed in duplicate. Two specimens challenge the lower and upper limits of the reportable range. The third specimen is somewhere in between.

Linearity

Several definitions are in common use. Among them:

- **Traditional Linearity** (CAP Visual Inspection): Draw a scatter plot with assigned values on the X-axis and measured mean on the Y-axis. If it looks like a straight line, the method is linear.
- **Statistical Linearity** (CLSI EP6P and EP6-A): These procedures determine acceptability based on statistical significance (i.e., p-values) rather than medical significance. EP Evaluator does not compute Statistical Linearity.
- **Clinical Linearity:** The method is linear if it is possible to draw a straight line that passes within a user-defined allowable error of each specimen point.

Related concepts:

- **Best Fit Line:** If the user opts to verify Linearity, this line it is obtained using the Clinical Linearity algorithm. Otherwise it is a regular linear regression line.
- **Outliers:** When verifying Linearity, the program first tries to determine an acceptable line using all specimens. If it fails, it then tries to find some subset of at least three specimens that are linear within allowable error. Specimens not in this acceptable subset are classified as outliers.
- **Slope and Intercept:** Coefficients of the Best Fit Line. The ideal slope is 1.00; the ideal intercept is zero.
- **Observed Error:** For Clinical Linearity, the minimum allowable error that could be defined for a data set and still have it be linear.
- **Standard Error of Estimate:** For regular regression, measures dispersion of the data points around the Best Fit Line.
- **Residual:** The difference between the best fit line and either an individual result or a mean measured value, depending on context.

Precision

Definition: Ability to obtain the same result upon repeated measurement of a specimen.

Verification Process: Measure the specimen many times. Compute the SD and CV, and verify that they are acceptably small. While 2-4 replicates are adequate for assessing accuracy, a minimum of 10 (and preferably 20 or more) is required to verify Precision.

The **Precision Index** is the ratio of SD to Allowable Random Error (defined below). The ideal -- and probably unattainable -- Precision Index is zero. A value of 1.00 indicates

Copyright 1991-2005 David G. Rhoads Associates, Inc.

Linearity

Report Interpretation Guide

borderline acceptability. Any further increase in SD would exceed allowable error.

The **95% Confidence Interval** (CI) for the Precision Index indicates how much sampling variation might be expected. The CI narrows as the number of replicates increases.

Allowable Total Error (TEa), and the Error Budget

TEa states the laboratory's policy for how much error is medically (or administratively) acceptable. Regulatory requirements represent an upper limit. Example: the CLIA limit for Sodium is 4 mmol/L.

Total Error has two major components: **Systematic Error** (synonym Bias) and **Random Error** (synonym Imprecision). The **Error Budget** allocates a fraction of the Allowable Total Error for Systematic Error, and another fraction for Random Error. Establishing an appropriate Error Budget allows the lab to control accuracy and precision separately, with reasonable confidence that Total Error will also remain in control. Recommended ranges of values are 25-50% for the Systematic Error Budget and 16-25% for the Random Error Budget.

Pass or Fail?

The program reports Pass/Fail for Accuracy and Linearity based on Allowable Systematic Error (SEa). Pass/Fail for Precision is based on Allowable Random Error (REa).

- A specimen passes Accuracy if its mean measured value is within SEa of the Assigned Value.
- The experiment passes Linearity if it is possible to draw a straight line (on the scatter plot of mean measured value vs. assigned value) that passes within +/- SEa of each specimen point.
- A specimen passes Precision if SD does not exceed REa.
- The experiment passes Reportable Range if 1) the assigned values of the lowest and highest specimens are within proximity limits of the Reportable Range Limits, and 2) these two specimens also pass accuracy.

Preliminary Report

The word PRELIMINARY printed diagonally across the report indicates that the data is incomplete, and the report is not acceptable as a final report. Some or all of the statistics may be missing.

The Linearity report is preliminary if there are less than three specimens.

Copyright 1991-2005 David G. Rhoads Associates, Inc.

EP Evaluator

Sirolimus

Biochemistry -- University Health Network

Instrument: Roche e411

Linearity

Scatter Plot

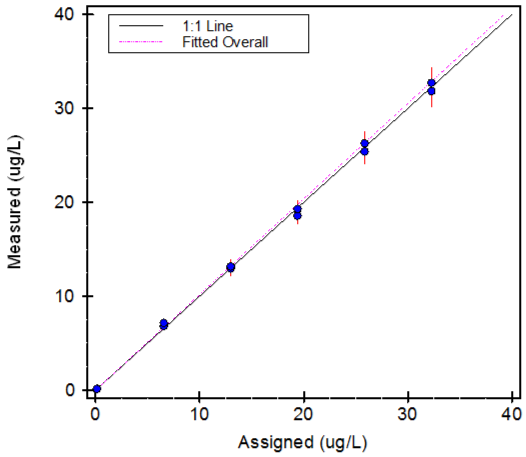

Residual Plot

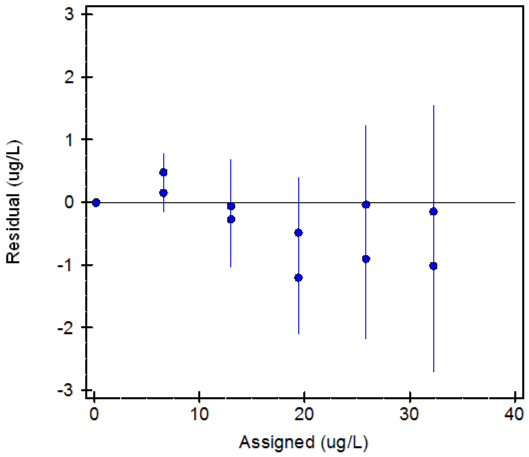

Linearity Summary

	N	Slope	Intercept	Error
Overall	6	1.018	0.004	4.5%

LINEAR within Allowable Systematic Error of 6.6%

Statistical Analysis and Experimental Results

	Assigned	Est	Mean	Resid	Linear?	Measured	Concentrations
Level 0	0.135	0.141	0.135	-0.006	Pass	0.14	0.13
Level 1	6.561	6.682	6.995	0.313	Pass	6.83	7.16
Level 2	12.987	13.224	13.055	-0.169	Pass	12.95	13.16
Level 3	19.413	19.765	18.920	-0.845	Pass	18.56	19.28
Level 4	25.840	26.308	25.835	-0.473	Pass	25.40	26.27
Level 5	32.266	32.849	32.265	-0.584	Pass	31.83	32.70

See User's Specifications for Pass/Fail criteria

X: Excluded from calculations

User's Specifications

Allowable Total Error:	20.0%
Systematic Error Budget:	33%
Allowable Systematic Error:	6.6%

Supporting Data

Analyst:	AWF
Date:	29 Jun 2016
Value Mode:	Preassigned
Units:	ug/L
Lot Number:	00187636
Comment:	

Analytical Claim

The Linearity of Sirolimus was analyzed on Roche e411 over a measured range of 0.135 to 32.265 ug/L. Allowable systematic error (SEa) was 6.6%. The results are LINEAR.

Accepted by: ______________________________ ______________

Signature Date

Copyright 1991-2005 David G. Rhoads Associates, Inc.

Linearity

Report Interpretation Guide

For EP Evaluator purposes, Linearity experiments are experiments that use specimens with defined concentrations. This includes Calibration Verification, Accuracy, and Reportable Range, as well as Linearity. The Linearity module can also verify Precision. This means you can verify three of the four CLIA '88 requirements with a single experiment.

User-selectable options determine which of these parameters the report verifies. Also, the user may request Pass/Fail flags against a specific allowable error criterion, or he/she may simply report selected statistical measures.

Experiment Procedure: Replicate measurements are made on 3-11 specimens, with (known) concentrations spread across the reportable range. Ideally, the lowest and highest specimens should challenge the limits of the range.

Accuracy (or Recovery)

Definition: The ability to recover the correct amount of analyte present in the specimen.

Verification process: Accuracy can be verified only when the "correct" amount of analyte (the **Assigned Value**) is known. While it is possible to determine recovery using a single replicate, one gets a more reliable estimate when 2 to 4 replicates are assayed.

Key statistic: Recovery = 100 x Measured Mean / Assigned Value

Reportable Range

Definition: As used in CLIA, this term refers to the Analytical Range or Assay Range -- the maximum range of values that can be assayed accurately without dilution. The CAP term "Analytical Measurement Range" (AMR) is a synonym for Reportable Range.

Verification Procedure: Reportable Range is verified if two conditions are met: 1) the assigned values of the lowest and highest specimens are within proximity limits of the Reportable Range limits, and 2) these two specimens are acceptably accurate.

Proximity Limits define how close the lowest and highest specimens must be to the Reportable Range limits.

Calibration Verification

verifies whether a method is properly calibrated. Calibration Verification is identical to verifying both Accuracy and Reportable Range. The only difference is that the report is titled to match the regulatory requirement.

CLIA requires a minimum of three specimens, each assayed in duplicate. Two specimens challenge the lower and upper limits of the reportable range. The third specimen is somewhere in between.

Linearity

Several definitions are in common use. Among them:

- **Traditional Linearity** (CAP Visual Inspection): Draw a scatter plot with assigned values on the X-axis and measured mean on the Y-axis. If it looks like a straight line, the method is linear.
- **Statistical Linearity** (CLSI EP6P and EP6-A): These procedures determine acceptability based on statistical significance (i.e., p-values) rather than medical significance. EP Evaluator does not compute Statistical Linearity.
- **Clinical Linearity:** The method is linear if it is possible to draw a straight line that passes within a user-defined allowable error of each specimen point.

Related concepts:

- **Best Fit Line:** If the user opts to verify Linearity, this line it is obtained using the Clinical Linearity algorithm. Otherwise it is a regular linear regression line.
- **Outliers:** When verifying Linearity, the program first tries to determine an acceptable line using all specimens. If it fails, it then tries to find some subset of at least three specimens that are linear within allowable error. Specimens not in this acceptable subset are classified as outliers.
- **Slope and Intercept:** Coefficients of the Best Fit Line. The ideal slope is 1.00; the ideal intercept is zero.
- **Observed Error:** For Clinical Linearity, the minimum allowable error that could be defined for a data set and still have it be linear.
- **Standard Error of Estimate:** For regular regression, measures dispersion of the data points around the Best Fit Line.
- **Residual:** The difference between the best fit line and either an individual result or a mean measured value, depending on context.

Precision

Definition: Ability to obtain the same result upon repeated measurement of a specimen.

Verification Process: Measure the specimen many times. Compute the SD and CV, and verify that they are acceptably small. While 2-4 replicates are adequate for assessing accuracy, a minimum of 10 (and preferably 20 or more) is required to verify Precision.

The **Precision Index** is the ratio of SD to Allowable Random Error (defined below). The ideal -- and probably unattainable -- Precision Index is zero. A value of 1.00 indicates

Copyright 1991-2005 David G. Rhoads Associates, Inc.

Linearity

Report Interpretation Guide

borderline acceptability. Any further increase in SD would exceed allowable error.

The **95% Confidence Interval** (CI) for the Precision Index indicates how much sampling variation might be expected. The CI narrows as the number of replicates increases.

Allowable Total Error (TEa), and the Error Budget

TEa states the laboratory's policy for how much error is medically (or administratively) acceptable. Regulatory requirements represent an upper limit. Example: the CLIA limit for Sodium is 4 mmol/L.

Total Error has two major components: **Systematic Error** (synonym Bias) and **Random Error** (synonym Imprecision). The **Error Budget** allocates a fraction of the Allowable Total Error for Systematic Error, and another fraction for Random Error. Establishing an appropriate Error Budget allows the lab to control accuracy and precision separately, with reasonable confidence that Total Error will also remain in control. Recommended ranges of values are 25-50% for the Systematic Error Budget and 16-25% for the Random Error Budget.

Pass or Fail?

The program reports Pass/Fail for Accuracy and Linearity based on Allowable Systematic Error (SEa). Pass/Fail for Precision is based on Allowable Random Error (REa).

- A specimen passes Accuracy if its mean measured value is within SEa of the Assigned Value.
- The experiment passes Linearity if it is possible to draw a straight line (on the scatter plot of mean measured value vs. assigned value) that passes within +/- SEa of each specimen point.
- A specimen passes Precision if SD does not exceed REa.
- The experiment passes Reportable Range if 1) the assigned values of the lowest and highest specimens are within proximity limits of the Reportable Range Limits, and 2) these two specimens also pass accuracy.

Preliminary Report

The word PRELIMINARY printed diagonally across the report indicates that the data is incomplete, and the report is not acceptable as a final report. Some or all of the statistics may be missing.

The Linearity report is preliminary if there are less than three specimens.

Copyright 1991-2005 David G. Rhoads Associates, Inc.

Sensitivity-Limit of Quantitation Summary

Instrument	*Analyte*	*Number of Periods*	*Number of Samples*	*Cutoff CV*	*LOQ*
Roche	✗ Cyclosporine	3	6	20%	< 6.47
	✗ Sirolimus	3	6	20%	< 0.05
	✗ Tacrolimus	3	6	20%	1.08

⚠ Required parameters are missing.

✗ Experiment 'fails' (or not enough results).

✔ Experiment 'passes' (or adequate number of results).

Copyright 1991-2005 David G. Rhoads Associates, Inc.

Sensitivity-Limit of Quantitation

Precision Profile

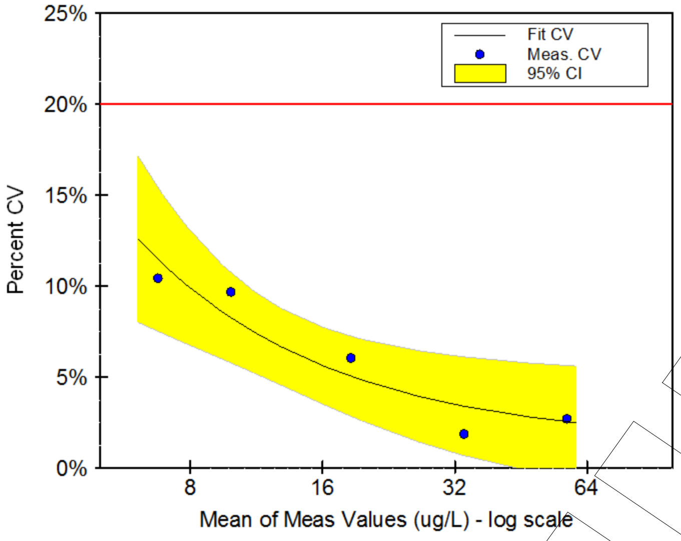

Analytical Claim

Cyclosporine was analyzed by Roche to determine the LOQ (lowest concentration for which CV is less than a target of 20%).

Specimens with mean measured concentration ranging from 6.757 to 57.527 ug/L were assayed. LOQ could not be estimated because all specimens had CVs less than the target. LOQ is less than 6.757 ug/L.

The report is marked PRELIMINARY because there are less than 10 data periods.

Sample		Target Conc.	N	Mean	SD	Meas. CV (%)	Fitted CV (%)	95% CI for Fitted Low	95% CI for Fitted High
Blank	x	6.47	2	6.470	1.075	16.6	11.9	7.7	16.1
1 in 32		8.26	3	6.757	0.706	10.4	11.5	7.5	15.4
1 in 16		9.66	3	9.893	0.959	9.7	8.3	5.8	10.8
1 in 4		19.23	3	18.553	1.124	6.1	5.1	2.9	7.3
1 in 2		32	3	33.553	0.633	1.9	3.4	0.7	6.1
Neat		57.53	3	57.527	1.565	2.7	2.6	-0.5	5.6

x: Excluded because either Mean or SD was zero.

Supporting Data

Analyst: AWF
Analysis Date: 26 May 2016
Units: ug/L
Target CV: 20%
Comment:

Fit Statistics

Model: --
A: --
B: --
R-square: --
Std Err Est: --

Accepted by: ______________________ Signature ______________ Date

PRELIMINARY

Copyright 1991-2005 David G. Rhoads Associates, Inc.

Sensitivity-Limit of Quantitation

Experimental Results

Sample	Result	Sample	Result	Sample	Result
1) 26 May 2016		***2) 26 May 2016***		***3) 26 May 2016***	
Neat	55.97	Neat	57.51	Neat	59.1
1 in 2	34.15	1 in 2	33.62	1 in 2	32.89
1 in 4	19.85	1 in 4	17.95	1 in 4	17.86
1 in 16	9.37	1 in 16	9.31	1 in 16	11.0
1 in 32	6.39	1 in 32	7.57	1 in 32	6.31
Blank	7.23	Blank	5.71		

"X" = Excluded Results

PRELIMINARY

Copyright 1991-2005 David G. Rhoads Associates, Inc.

EP Evaluator

Biochemistry -- University Health Network

Sirolimus

Instrument: Roche

Sensitivity-Limit of Quantitation

Precision Profile

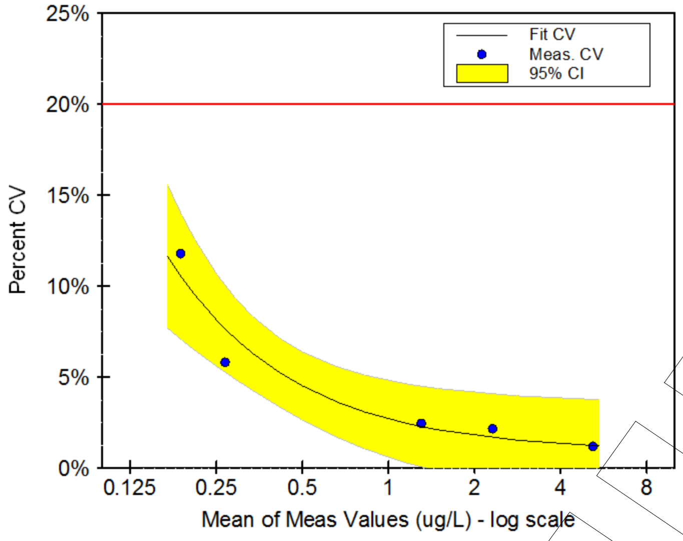

Analytical Claim

Sirolimus was analyzed by Roche to determine the LOQ (lowest concentration for which CV is less than a target of 20%).

Specimens with mean measured concentration ranging from 0.188 to 5.190 ug/L were assayed. LOQ could not be estimated because all specimens had CVs less than the target. LOQ is less than 0.188 ug/L.

The report is marked PRELIMINARY because there are less than 10 data periods.

Sample		Target Conc.	N	Mean	SD	Meas. CV (%)	Fitted CV (%)	95% CI for Fitted Low	95% CI for Fitted High
Blank	x	0.05	2	0.053	0.001	2.7	35.2	19.9	50.5
1 in 32		0.22	3	0.188	0.022	11.8	10.6	7.1	14.1
1 in 16		0.37	3	0.268	0.016	5.8	7.7	5.3	10.1
1 in 4		1.34	3	1.303	0.032	2.5	2.3	0.1	4.5
1 in 2		2.62	3	2.313	0.050	2.2	1.7	-0.7	4.1
Neat		5.19	3	5.190	0.062	1.2	1.3	-1.3	3.8

x: Excluded because either Mean or SD was zero.

Supporting Data

Analyst: AWF
Analysis Date: 26 May 2016
Units: ug/L
Target CV: 20%
Comment:

Fit Statistics

Model: --
A: --
B: --
R-square: --
Std Err Est: --

Accepted by: ______________________ Signature ______________ Date

PRELIMINARY

Sensitivity-Limit of Quantitation

Experimental Results

Sample	Result	Sample	Result	Sample	Result
1) 26 May 2016		***2) 26 May 2016***		***3) 26 May 2016***	
Neat	5.14	Neat	5.26	Neat	5.17
1 in 2	2.26	1 in 2	2.32	1 in 2	2.36
1 in 4	1.34	1 in 4	1.28	1 in 4	1.29
1 in 16	0.28	1 in 16	0.25	1 in 16	0.27
1 in 32	0.17	1 in 32	0.21	1 in 32	0.18
Blank	0.05	Blank	0.05		

"X" = Excluded Results

PRELIMINARY

Copyright 1991-2005 David G. Rhoads Associates, Inc.

Sensitivity-Limit of Quantitation

Precision Profile

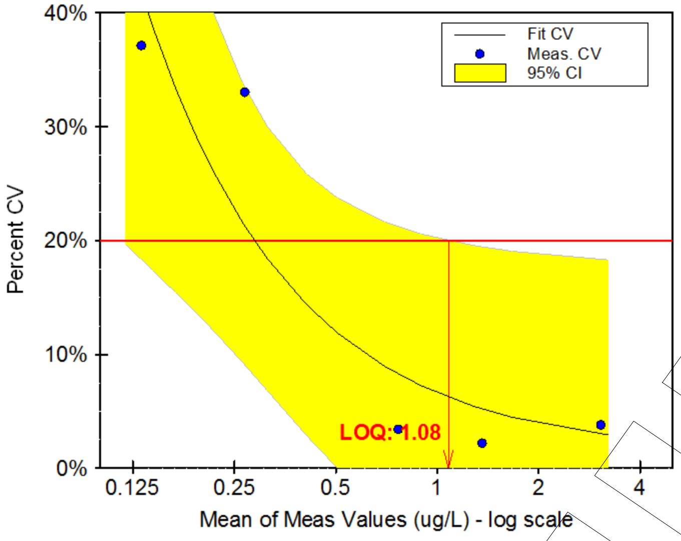

Analytical Claim

Tacrolimus was analyzed by Roche to determine the LOQ (lowest concentration for which CV is less than a target of 20%).

Specimens with mean measured concentration ranging from 0.132 to 3.063 ug/L were assayed. A curve was fit to estimate the relationship between Mean and CV. Based on the fitted model, the LOQ is 1.08 ug/L. This is the point where the upper 95% confidence interval for the curve has a CV of 20%.

The report is marked PRELIMINARY because there are less than 10 data periods.

Sample		Target Conc.	N	Mean	SD	Meas. CV (%)	Fitted CV (%)	95% CI for Fitted Low	95% CI for Fitted High
1 in 32		0.24	3	0.132	0.049	37.1	41.8	18.3	65.3
blank	x	0.14	2	0.143	0.050	35.0	38.7	17.3	60.0
1 in 16		0.33	3	0.268	0.089	33.0	21.3	9.1	33.5
1 in 4		0.87	3	0.766	0.026	3.4	8.3	-4.7	21.2
**Estimated LOQ**			**–**	**1.08**	**–**	**–**	**6.2**	**-7.5**	**20.0**
1 in 2		1.60	3	1.360	0.030	2.2	5.2	-9.0	19.4
Neat		3.06	3	3.063	0.117	3.8	3.0	-12.3	18.3

x: Excluded because either Mean or SD was zero.

Supporting Data

Analyst:	AWF
Analysis Date:	26 May 2016
Units:	ug/L
Target CV:	20%
Comment:	

Fit Statistics

Model:	CV = A + B * (1/Mean)
A:	1.264 +/- 5.119
B:	5.369 +/- 1.337
R-square:	0.8432
Std Err Est:	8.03

Accepted by:

Signature ______________________ Date ______________

Copyright 1991-2005 David G. Rhoads Associates, Inc.

PRELIMINARY

PRELIMINARY

Sensitivity-Limit of Quantitation

Experimental Results

Sample	Result	Sample	Result	Sample	Result
1) 26 May 2016		***2) 26 May 2016***		***3) 26 May 2016***	
Neat	3.19	Neat	2.96	Neat	3.04
1 in 2	1.39	1 in 2	1.36	1 in 2	1.33
1 in 4	0.80	1 in 4	0.75	1 in 4	0.76
1 in 16	0.34	1 in 16	0.29	1 in 16	0.17
1 in 32	0.19	1 in 32	0.10	1 in 32	0.11
blank	0.18	blank	0.11		

"X" = Excluded Results

Copyright 1991-2005 David G. Rhoads Associates, Inc.

Sensitivity-Limit of Quantitation

Report Interpretation Guide

The Limit of Quantitation (LOQ) is the lowest concentration at which an acceptable CV (Target CV) can be obtained over a long period (weeks or months). This is sometimes called functional sensitivity.

LOQ is important for analytes like TSH (thyrotropin), where the low end of the analytical range is clinically significant.

Experiment Procedure

- Define the Target CV (usually 20%).
- Prepare several (5-10) specimens that cover the range of concentrations where you expect the Target CV to occur.

  Concentrations should increase logarithmically (a series like 1, 2, 5, 10, 20) rather than linearly (2, 4, 6, 8, 10, 12). If you use a logarithmic series, you do not have to assay as many specimens. If you prepare the specimens using a series of serial dilutions, make sure the analyte is stable in your dilutent.
- Make 20 to 50 aliquots of each specimen, and freeze them. Assay each specimen every day, for at least 10 days. Continuing the experiment for more than 10 days substantially improves the quality of the result. We recommend continuing for at least 20 days.
- From these results, EP Evaluator calculates the CV at each concentration. The LOQ is the lowest concentration for which the CV is less than the Target CV.

Definitions

Target CV (or Cutoff): The Largest acceptable CV. The Limit of Quantitation is the lowest concentration that has a CV less than the Target CV.

Target Concentrations: The assigned concentrations of the specimens. Target concentrations are printed on the report, but are not used to calculate LOQ.

N: Number of unexcluded results (i.e., days) for each specimen.

Mean: Average value for each specimen over the duration of the experiment.

Standard Deviation (SD): Measures the variation of a specimen's results about the mean, in concentration units. Note that this is an ordinary SD. It is NOT an EP5 total SD determined from ANOVA analysis of multiple runs per day.

Measured Coefficient of Variation (CV): SD expressed as a percent of the mean.

Precision Profile: A graph that shows the relationship between measured Mean and CV.

Fitted CV: *Predicted* CV, determined by fitting a curve of the form CV = A + B * (1/Mean) to the Precision Profile. Fitted values appear on the report only if there are at least five unexcluded specimens.

95% Confidence Interval (CI): If a curve was fitted the column is labelled "95% CI for Fitted", and the confidence interval reflects the quality of the fitted model. Otherwise the column is labelled "95% CI for Meas", and the confidence interval reflects error in the point estimate.

Report Interpretation

With five or more specimens ... The program fits a curve of the form CV = A + B * (1/Mean) to the Precision Profile. The estimated LOQ is the point where the upper 95% confidence limit for the fitted curve is equal to the Target CV.

The fitted curve is never extrapolated outside the range of the measured means. In other words, the LOQ is estimated only if there is at least one measured CV above the target, and one below it.

Confidence limits are determined from the curve fit. These limits are based on error in the fitted model, not error of the individual point measurements.

With 3-4 specimens ... The program tabulates means, CVs, and confidence intervals for the individual specimens, but makes no attempt to interpolate between these points to estimate an LOQ.

Confidence limits measure the error in estimating an individual (point-estimate) CV. These limits are typically much wider are limits obtained from fitting a curve. We recommend using at least five specimens, with the largest specimen chosen so its CV is well below the target CV.

Preliminary Report

The word PRELIMINARY printed diagonally across the report indicates that the data is incomplete, and the report is not acceptable as a final report. Some or all of the statistics may be missing. Causes:

- Less than 10 days of data.
- Less than 3 specimens with both a non-zero mean and a non-zero SD. The SD for a specimen is zero if it has less than two non-excluded results (days), or if all of its results are exactly the same on every day.

Copyright 1991-2005 David G. Rhoads Associates, Inc.

Alternate Method Comparison Summary

Y Method		X Method	Analyte	N	Slope	Intercept	Corr (R)
e411	✔	i2000	Cyclosporine	100/100	0.917	-15.19	0.9849
	✔	i2000	Tacrolimus	100/100	0.938	0.20	0.9738
	✔	LC/MSMS	Cyclosporine	20/100	1.331	-67.96	0.9687
	✔	LC/MSMS	Tacrolimus	20/100	0.924	-0.19	0.9844
e411(lot2)	✔	e411	Cyclosporine	20/100	0.998	8.48	0.9980
	✔	e411	Tacrolimus	20/100	0.972	-0.40	0.9972
i2000	✔	LC/MSMS	Cyclosporine	20/100	1.454	-42.84	0.9855
	✔	LC/MSMS	Tacrolimus	20/100	0.982	-0.20	0.9656

- Required parameters are missing.
- ✔ Experiment 'passes' (or adequate number of results).
- ✘ Experiment 'fails' (or not enough results).
- Experiment has outliers or subrange analysis.

Copyright 1991-2005 David G. Rhoads Associates, Inc.

Alternate (Quantitative) Method Comparison

X Method: i2000 **Y Method: e411**

Scatter Plot

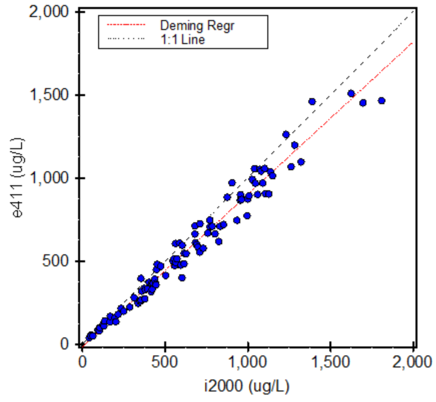

Bias

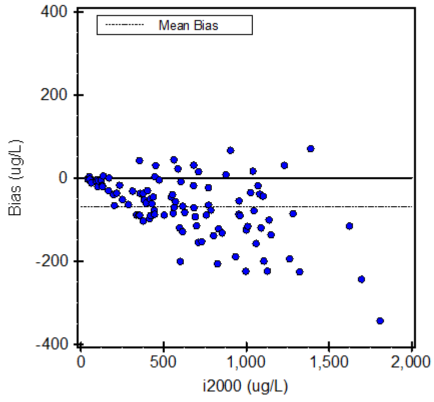

Percent Bias

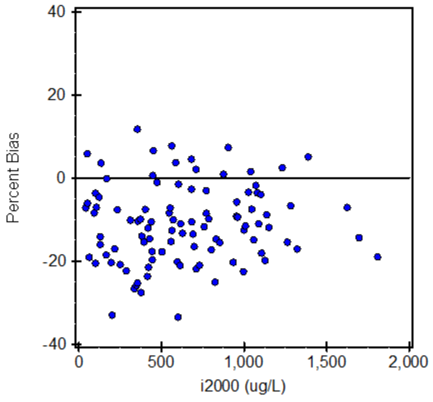

Regression Analysis

	Deming	Regular
**Slope:**	0.917 (0.885 to 0.949)	0.904 (0.872 to 0.936)
**Intercept:**	-15.19 (-39.38 to 8.99)	-7.01 (-31.12 to 17.09)
**Std Err Est:**	63.54	63.34

95% Confidence Intervals are shown in parentheses

Supporting Statistics

Corr Coef (R):	0.9849	SubRange Bounds:	None
Bias:	-68.98	Points (Plotted/Total):	100/100
X Mean ± SD:	645.42 ± 396.35	Outliers:	None
Y Mean ± SD:	576.45 ± 363.80	Scatter Plot Bounds:	None
Std Dev Diffs:	73.62		

Experiment Description

	X Method	Y Method
Expt Date:	27 May 2015	27 May 2015
Rep SD:	1	1
Result Ranges:	41.0 to 1808.0	38.0 to 1508.0
Units:	ug/L	ug/L
Analyst:	MK	MK
Comment:		

Accepted by: ______________________________ ____________________

Signature Date

Copyright 1991-2005 David G. Rhoads Associates, Inc.

Alternate (Quantitative) Method Comparison

X Method: i2000 **Y Method: e411**

Experimental Results

	Spec ID	Results X	Results Y	Bias	Calc'd Y	SEE Factor
103	YVQ30502	41	38.0	-3.0	22.39	0.2
104	UAH61177	52	48.8	-3.2	32.47	0.3
105	UAH60296	52	55.0	3.0	32.47	0.4
106	UAH60289	63	51.0	-12.0	42.56	0.1
107	UAH61006	93.1	85.2	-7.9	70.15	0.2
108	YXG54802	101	97.3	-3.7	77.39	0.3
109	UAH61012	101.4	80.6	-20.8	77.76	0.0
110	UAH60216	106	98.5	-7.5	81.97	0.3
111	UAH60292	123	117.3	-5.7	97.56	0.3
112	UAH60993	129.5	111.2	-18.3	103.52	0.1
113	UAH61010	129.7	108.9	-20.8	103.70	0.1
114	UAH60291	135	139.8	4.8	108.56	0.5
115	UAH60268	166	135.2	-30.8	136.97	0.0
116	UAH61174	168.4	168.1	-0.3	139.17	0.5
117	UAH61005	196.5	156.5	-40.0	164.93	-0.1
118	UAH60984	201.9	135.3	-66.6	169.88	-0.5
119	YVO25503	217	179.9	-37.1	183.72	-0.1
120	120029281	233.4	215.5	-17.9	198.76	0.3
121	UAH60992	250.3	198.1	-52.2	214.25	-0.3
122	UAH61007	286.7	222.6	-64.1	247.62	-0.4
123	UAH61127	312	280.4	-31.6	270.81	0.2
124	UAH60986	335.4	246	-89.4	292.26	-0.7
125	UAH61011	348.3	258.3	-90.0	304.08	-0.7
126	UAH61169	354	395.5	41.5	309.31	1.4
127	UAH61009	355.1	265.3	-89.8	310.32	-0.7
128	UAH61172	357.1	319.9	-37.2	312.15	0.1
129	31136348	374	336.8	-37.2	327.64	0.1
130	UAH61003	376.7	272.8	-103.9	330.12	-0.9
131	YYP28903	381	327.6	-53.4	334.06	-0.1
132	UAH60991	393.6	332.7	-60.9	345.61	-0.2
133	YVK66802	403	372.4	-30.6	354.22	0.3
134	31137166	415	316.6	-98.4	365.22	-0.8
135	UAH60275	419	368.6	-50.4	368.89	0.0
136	UAH61008	421.1	330.5	-90.6	370.82	-0.6
137	UAH61109	428.7	366	-62.7	377.78	-0.2
138	UAH61094	437.7	391.5	-46.2	386.03	0.1
139	UAH61121	443.1	364.5	-78.6	390.98	-0.4
140	UAH61156	444.6	357	-87.6	392.36	-0.6
141	YXB17502	447	449.7	2.7	394.56	0.9
142	UAH61166	451.5	481.2	29.7	398.68	1.3
143	ABX89603	473.9	468.9	-5.0	419.22	0.8
144	UAH61100	503.4	414	-89.4	446.26	-0.5
145	UAH61151	547.1	500.9	-46.2	486.32	0.2
146	YWE11002	554	513.8	-40.2	492.64	0.3
147	UAH61123	557.6	472.1	-85.5	495.94	-0.4
148	UAH61147	562.1	605.3	43.2	500.07	1.7
149	UAH60234	563	491.9	-71.1	500.89	-0.1
150	UAH60242	571	513.4	-57.6	508.22	0.1
151	ACK91202	586.2	607.8	21.6	522.16	1.3
152	UAH61137	595.9	475.7	-120.2	531.05	-0.9
153	UAH60985	601.1	399.9	-201.2	535.82	-2.1
154	YWY92203	604	595	-9	538.47	0.9
155	ZYQ85102	613.2	483.8	-129.4	546.91	-1.0
156	ZYP15903	615.5	547.7	-67.8	549.02	0.0
157	ZYA77703	627.8	544.4	-83.4	560.29	-0.3
158	ACE32303	680.9	711.4	30.5	608.97	1.6
159	UAH60264	681	662.6	-18.4	609.06	0.8
160	UAH61165	683	610.8	-72.2	610.89	0.0
161	ZYQ55004	691.4	598	-93.4	618.59	-0.3
162	YWS77103	699	583.6	-115.4	625.56	-0.7
163	YTH39103	710	554.5	-155.5	635.64	-1.3
164	ZYO78603	710	724.7	14.7	635.64	1.4
165	YXB86803	730	576.6	-153.4	653.98	-1.2
166	UAH61148	757.8	668.8	-89.0	679.46	-0.2
167	ZYN23003	770.3	746.7	-23.6	690.92	0.9
168	ACU43302	771.3	705.7	-65.6	691.83	0.2
169	YVH98502	786	708.7	-77.3	705.31	0.1
170	YXI77903	802	663.3	-138.7	719.98	-0.9
171	UAH61096	825.1	618.4	-206.7	741.15	-1.9
172	ZYO00403	831.8	709.2	-122.6	747.29	-0.6
173	UAH61017	853.2	720.5	-132.7	766.91	-0.7
174	YXH13902	876	883.6	7.6	787.81	1.5
175	UAH61034	903.9	970.1	66.2	813.38	2.5
176	31272306	934.6	744.9	-189.7	841.53	-1.5
177	ZYK07703	953.8	866.2	-87.6	859.13	0.1
178	ACK08903	955.7	900.1	-55.6	860.87	0.6
179	UAH61162	961.4	870.5	-90.9	866.09	0.1
180	UAH60996	996.6	771.9	-224.7	898.36	-2.0
181	YVN56803	999	873.3	-125.7	900.56	-0.4
182	31298365	1008.2	891.5	-116.7	908.99	-0.3
183	AAO89903	1026.4	991.2	-35.2	925.68	1.0
184	UAH60290	1040	1056	16	938.14	1.9
185	UAH61212	1046.6	967.7	-78.9	944.19	0.4
186	UAH60948	1058.3	900.6	-157.7	954.92	-0.9
187	AEZ70303	1071.1	1052	-19.1	966.65	1.3
188	TNJ27502	1080	1041	-39	974.81	1.0
189	AEJ98903	1089.7	969.5	-120.2	983.70	-0.2
190	YRK12903	1101	1057	-44	994.06	1.0
191	YXI44303	1106	906.1	-199.9	998.64	-1.5
192	UAH61176	1127.7	903.3	-224.4	1018.53	-1.8
193	TPJ51503	1138	1037	-101	1027.98	0.1
194	TNM00102	1150	1013	-137	1038.98	-0.4
195	TRG37204	1231	1261	30	1113.23	2.3
196	3037895	1262	1067	-195	1141.64	-1.2
197	ZFH97703	1283	1197	-86	1160.89	0.6
198	UAH61256	1322	1096	-226	1196.64	-1.6
199	TNM23403	1389	1459	70	1258.06	3.2
200	UAH60698	1624	1508	-116	1473.48	0.5
201	UAH59327	1696	1452	-244	1539.48	-1.4
202	ZYN41003	1808	1464	-344	1642.14	-2.8

Values marked with an "X" were excluded from the calculations. Outliers "O" were also excluded.

Copyright 1991-2005 David G. Rhoads Associates, Inc.

Alternate (Quantitative) Method Comparison

X Method: i2000 **Y Method: e411**

Scatter Plot

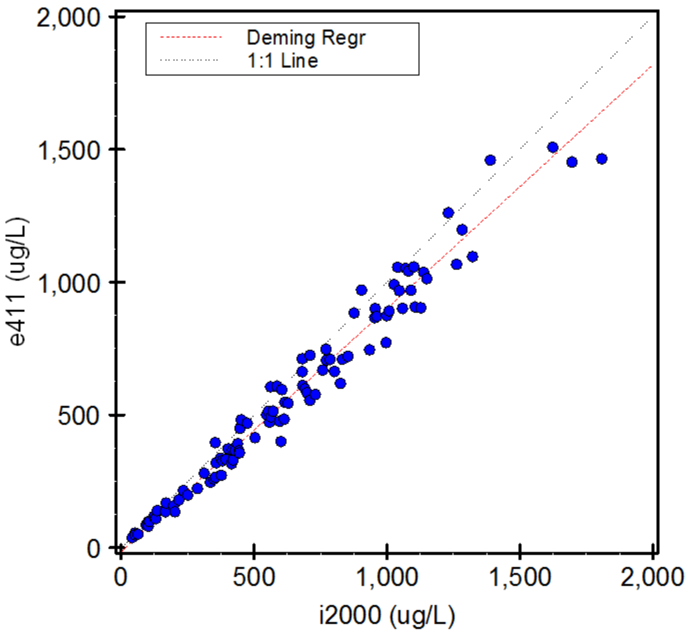

Bias

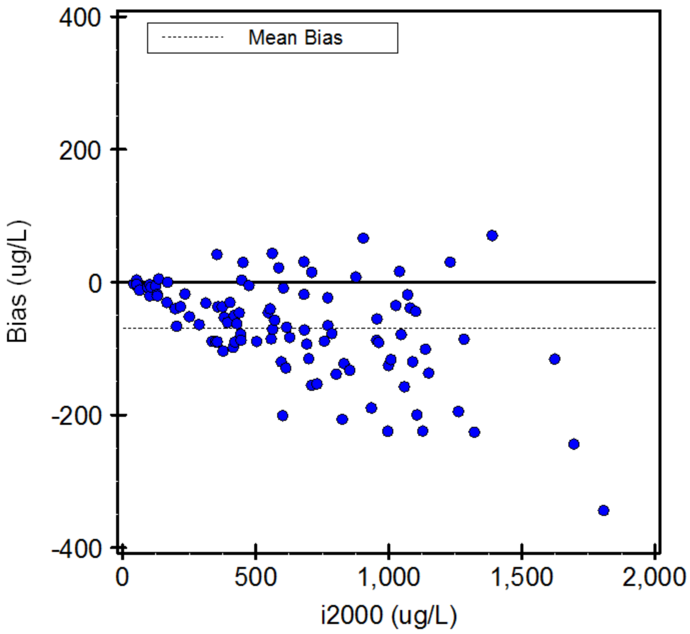

Percent Bias

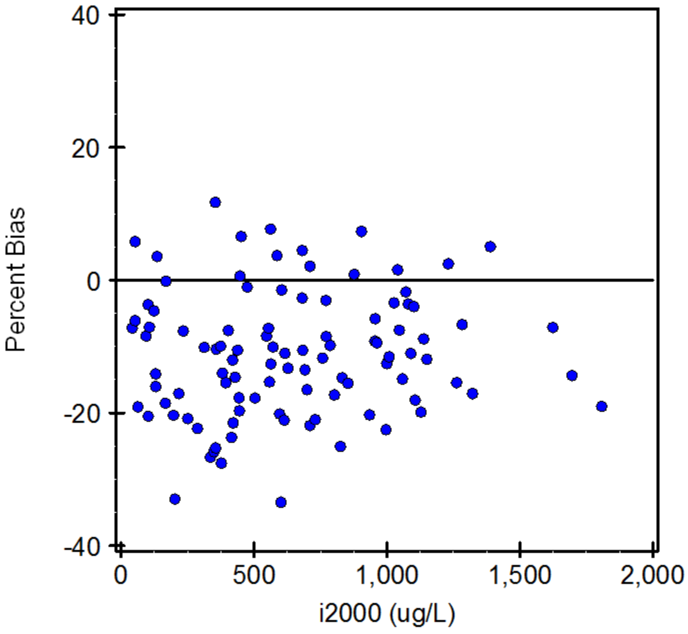

This page contains a larger, working copy of the same graphs that appear on page 1.

Copyright 1991-2005 David G. Rhoads Associates, Inc.

Alternate (Quantitative) Method Comparison

X Method: i2000 **Y Method: e411**

Scatter Plot

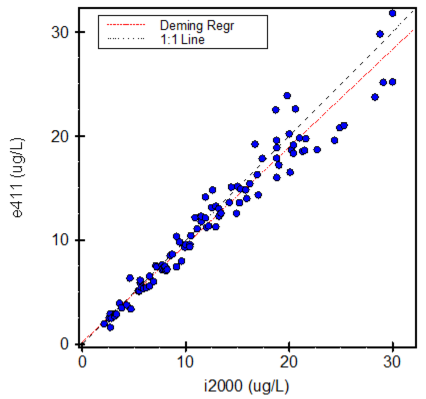

Bias

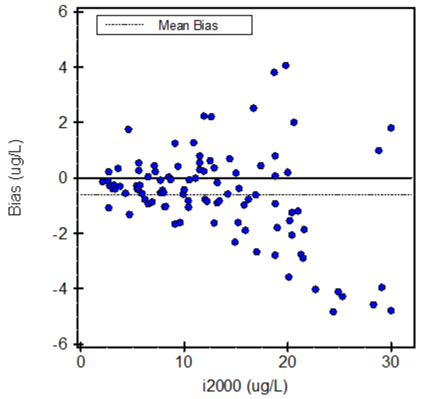

Percent Bias

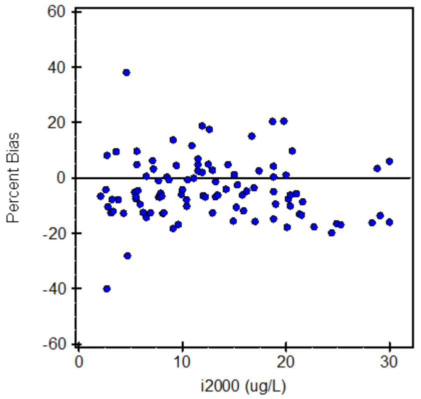

Regression Analysis

	Deming	Regular
**Slope:**	0.938 (0.895 to 0.981)	0.915 (0.872 to 0.957)
**Intercept:**	0.20 (-0.43 to 0.83)	0.50 (-0.13 to 1.13)
**Std Err Est:**	1.54	1.53

95% Confidence Intervals are shown in parentheses

Supporting Statistics

Corr Coef (R):	0.9738	SubRange Bounds:	None
Bias:	-0.60	Points (Plotted/Total):	100/100
X Mean ± SD:	12.87 ± 7.11	Outliers:	Not Tested
Y Mean ± SD:	12.26 ± 6.68	Scatter Plot Bounds:	None
Std Dev Diffs:	1.64		

Experiment Description

	X Method	Y Method
Expt Date:	27 May 2015	27 May 2015
Rep SD:	1	1
Result Ranges:	2.1 to 30.0	1.6 to 31.8
Units:	ug/L	ug/L
Analyst:	MK	MK
Comment:		

Accepted by: ______________________________ ______________

Signature Date

Copyright 1991-2005 David G. Rhoads Associates, Inc.

Alternate (Quantitative) Method Comparison

X Method: i2000 **Y Method: e411**

Experimental Results

	Spec ID	Results X	Results Y	Bias	Calc'd Y	SEE Factor
102	ZIO88003	2.1	2.0	-0.1	2.17	-0.1
103	UAJ6458	2.6	2.5	-0.1	2.64	-0.1
104	UAJ6452	2.7	1.6	-1.1	2.73	-0.7
105	YVM26903	2.7	2.9	0.2	2.73	0.1
106	UAJ6444	2.8	2.5	-0.3	2.83	-0.2
107	UAJ6472	3.1	2.7	-0.4	3.11	-0.3
108	31197733	3.2	3.0	-0.3	3.20	-0.2
109	ZJE32203	3.3	2.9	-0.4	3.29	-0.3
110	UAJ5763	3.6	3.9	0.3	3.58	0.2
111	ZIY67902	3.8	3.5	-0.3	3.76	-0.2
112	UAJ6468	4.3	3.8	-0.5	4.23	-0.3
113	YVH85302	4.6	6.3	1.8	4.51	1.2
114	UAJ6449	4.7	3.4	-1.3	4.61	-0.8
115	UAJ6488	5.4	5.1	-0.3	5.26	-0.1
116	UAJ6424	5.5	5.1	-0.4	5.36	-0.2
117	UAJ6409	5.5	5.1	-0.4	5.36	-0.2
118	UAJ5753	5.6	5.9	0.3	5.45	0.3
119	UAJ6441	5.6	6.1	0.5	5.45	0.4
120	UAJ6448	5.7	5.4	-0.3	5.55	-0.1
121	UAJ6443	5.9	5.3	-0.6	5.73	-0.3
122	UAJ6456	6.2	5.4	-0.8	6.01	-0.4
123	UAJ6401	6.5	6.5	0.0	6.30	0.2
124	UAJ6428	6.5	5.6	-0.9	6.30	-0.5
125	UAJ6447	6.9	6.0	-0.9	6.67	-0.4
126	UAJ6410	7.1	7.5	0.4	6.86	0.4
127	YQX43302	7.2	7.4	0.2	6.95	0.3
128	UAJ6461	7.7	7.6	-0.1	7.42	0.1
129	UAJ6446	7.7	7.2	-0.5	7.42	-0.2
130	UAJ6439	7.8	7.3	-0.5	7.51	-0.2
131	UAJ6429	7.9	7.5	-0.4	7.61	-0.1
132	UAJ6442	8	7.5	-0.5	7.70	-0.2
133	UAJ6421	8.1	7.1	-1.0	7.80	-0.5
134	UAJ6412	8.2	7.2	-1.0	7.89	-0.5
135	UAJ5620	8.5	8.5	0.0	8.17	0.2
136	UAJ6451	8.7	8.6	-0.1	8.36	0.2
137	YVP40704	9.1	10.3	1.3	8.73	1.1
138	UAJ6422	9.1	7.4	-1.7	8.73	-0.8
139	UAJ6404	9.4	9.8	0.4	9.01	0.5
140	UAJ6450	9.6	8.0	-1.6	9.20	-0.8
141	UAJ6402	9.9	9.3	-0.6	9.48	-0.1
142	YVH98503	10	9.6	-0.4	9.58	0.0
143	ZJM20004	10.4	9.3	-1.1	9.95	-0.4
144	UAJ6423	10.4	9.6	-0.8	9.95	-0.2
145	UAJ6445	10.5	10.4	-0.1	10.05	0.2
146	UAJ5794	10.9	12.2	1.3	10.42	1.1
147	UAJ6467	11.1	11.1	0.0	10.61	0.3
148	ZKB4603	11.5	12.3	0.8	10.98	0.9
149	UAJ6460	11.5	12.1	0.6	10.98	0.7
150	ZIM40003	11.5	11.8	0.3	10.98	0.5
151	UAJ5755	11.9	14.1	2.2	11.36	1.8
152	UAJ6454	11.9	12.1	0.2	11.36	0.5
153	UAJ6434	12	11.2	-0.8	11.45	-0.1
154	UAJ6453	12.2	11.4	-0.8	11.64	-0.2
155	ZIQ08702	12.5	13.1	0.6	11.92	0.8
156	YUZ03304	12.6	14.8	2.2	12.02	1.8
157	UAJ6438	12.9	13.3	0.4	12.30	0.6
158	UAJ6419	12.9	11.3	-1.6	12.30	-0.7
159	UAJ6469	13.2	13.0	-0.2	12.58	0.3
160	ZJA38902	13.2	12.3	-0.9	12.58	-0.2
161	UAJ6440	13.4	12.6	-0.8	12.77	-0.1
162	UAJ6436	14.2	13.6	-0.6	13.52	0.1
163	YRA78903	14.4	15.1	0.7	13.70	0.9
164	UAJ6475	14.9	12.6	-2.3	14.17	-1.0
165	UAJ6903	15	15.2	0.2	14.27	0.6
166	UAJ6853	15.2	13.6	-1.6	14.45	-0.6
167	31113015	15.3	14.9	-0.4	14.55	0.2
168	UAJ6427	15.8	14.8	-1.0	15.02	-0.1
169	ZQX95203	15.9	14.0	-1.9	15.11	-0.7
170	UAJ6745	16.2	15.4	-0.8	15.39	0.0
171	UAJ5808	16.7	19.2	2.5	15.86	2.2
172	ZRG65302	16.9	16.3	-0.6	16.05	0.2
173	UAJ6149	17	14.3	-2.7	16.14	-1.2
174	UAJ6916	17.4	17.8	0.4	16.52	0.9
175	UAJ5736	18.7	22.5	3.8	17.74	3.1
176	UAJ6430	18.8	17.9	-0.9	17.83	0.0
177	UAJ7049	18.8	19.6	0.8	17.83	1.1
178	UAJ7025	18.8	16.0	-2.8	17.83	-1.2
179	ZSS39603	18.8	18.9	0.1	17.83	0.7
180	UAJ6902	19	17.2	-1.8	18.02	-0.5
181	UAJ6794	19	17.2	-1.8	18.02	-0.5
182	YVA63003	19.8	23.9	4.1	18.77	3.3
183	UAJ7055	20	20.2	0.2	18.95	0.8
184	UAJ6185	20.1	16.5	-3.6	19.05	-1.6
185	UAJ6426	20.2	18.7	-1.5	19.14	-0.3
186	UAJ5679	20.4	19.1	-1.3	19.33	-0.1
187	UAJ5977	20.4	18.3	-2.1	19.33	-0.6
188	ZUH12003	20.6	22.6	2.0	19.52	2.0
189	UAJ5792	21	19.8	-1.2	19.89	-0.1
190	YZY99904	21.3	18.5	-2.8	20.17	-1.1
191	UAJ6151	21.5	18.6	-2.9	20.36	-1.1
192	UAJ6464	21.6	19.7	-1.9	20.45	-0.5
193	UAJ6014	22.7	18.7	-4.0	21.49	-1.8
194	UAJ6009	24.4	19.6	-4.8	23.08	-2.3
195	YYS62303	24.9	20.8	-4.1	23.55	-1.8
196	UAJ6030	25.3	21.0	-4.3	23.92	-1.9
197	UAJ6755	28.3	23.7	-4.6	26.74	-2.0
198	UAJ5728	28.8	29.8	1.0	27.21	1.7
199	UAJ6807	29.1	25.1	-4.0	27.49	-1.5
200	UAJ5607	30	25.2	-4.8	28.33	-2.0
201	UAJ6802	30	31.8	1.8	28.33	2.3

Values marked with an "X" were excluded from the calculations.

Copyright 1991-2005 David G. Rhoads Associates, Inc.

Alternate (Quantitative) Method Comparison

X Method: i2000 **Y Method: e411**

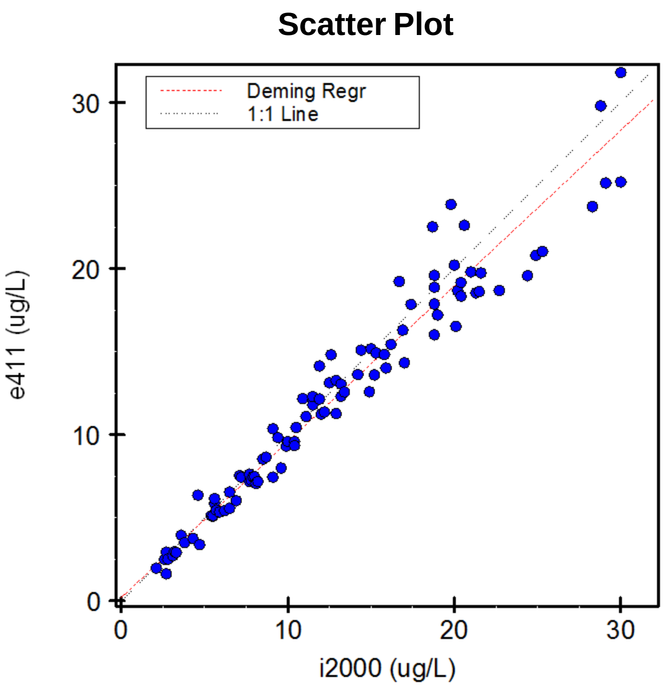

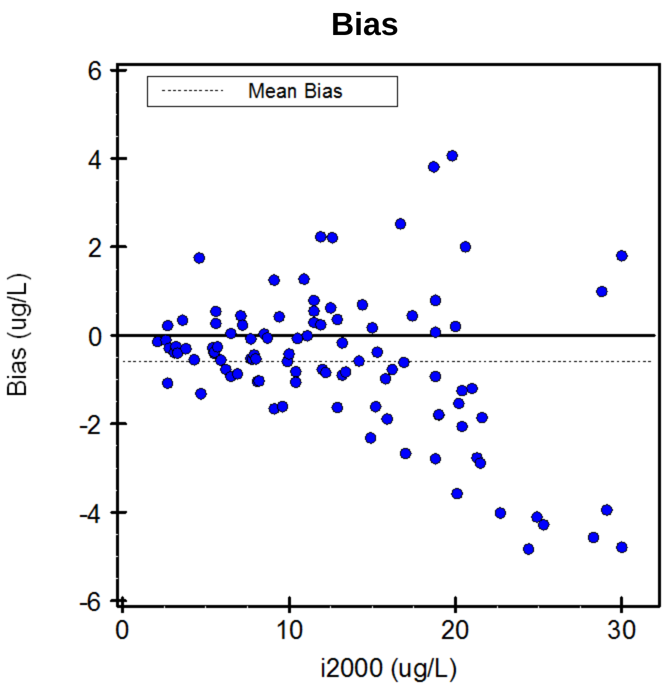

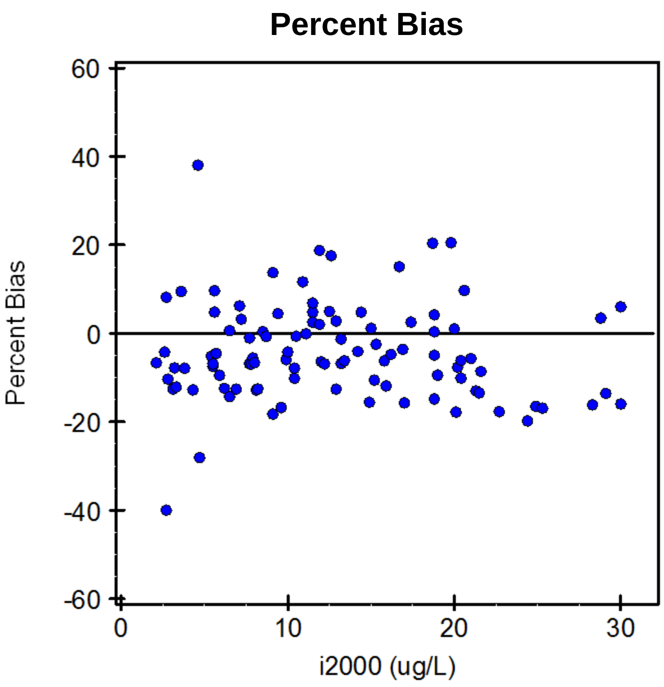

This page contains a larger, working copy of the same graphs that appear on page 1.

Copyright 1991-2005 David G. Rhoads Associates, Inc.

Alternate (Quantitative) Method Comparison

X Method: LC/MSMS **Y Method: e411**

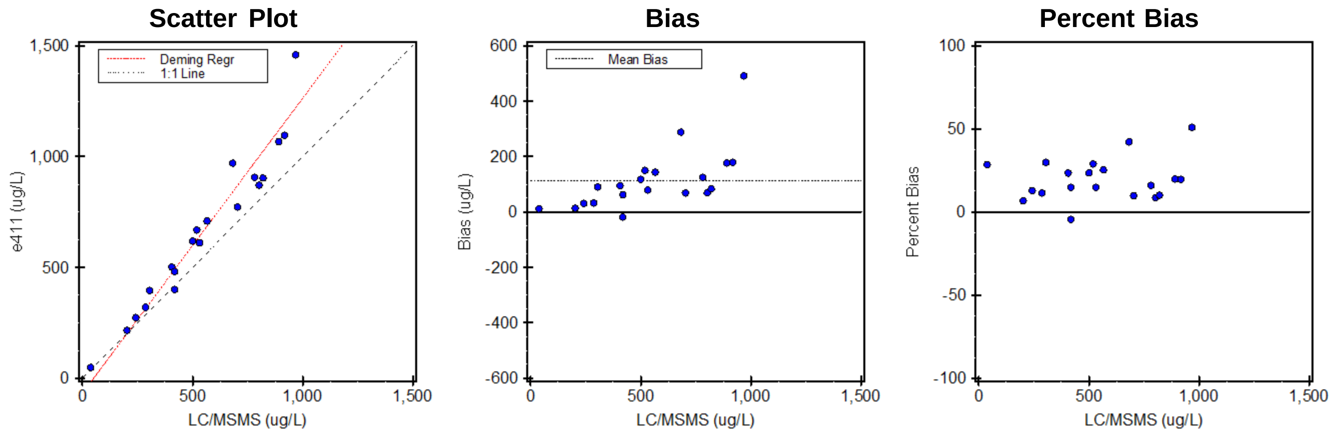

Regression Analysis

	Deming	Regular
**Slope:**	1.331 (1.167 to 1.496)	1.279 (1.116 to 1.441)
**Intercept:**	-67.96 (-167.73 to 31.82)	-38.87 (-137.37 to 59.63)
**Std Err Est:**	89.98	88.83

95% Confidence Intervals are shown in parentheses

Supporting Statistics

Corr Coef (R):	0.9687	SubRange Bounds:	None
Bias:	114.31	Points (Plotted/Total):	20/100
X Mean ± SD:	549.97 ± 263.89	Outliers:	None
Y Mean ± SD:	664.28 ± 348.29	Scatter Plot Bounds:	None
Std Dev Diffs:	113.48		

Experiment Description

	X Method	Y Method
Expt Date:	27 May 2015	27 May 2015
Rep SD:	1	1
Result Ranges:	38.0 to 967.7	48.8 to 1459.0
Units:	ug/L	ug/L
Analyst:	MK	MK
Comment:		

Accepted by:

Signature _______________ Date _______________

Copyright 1991-2005 David G. Rhoads Associates, Inc.

Alternate (Quantitative) Method Comparison

X Method: LC/MSMS **Y Method: e411**

Experimental Results

	Spec ID		Results X	Results Y	Bias	Calc'd Y	SEE Factor
63	ZFH97703	X		1197			
65	ZYO78603	X		724.7			
66	ZYN41003	X		1464			
67	ZYN23003	X		746.7			
68	ZYQ85102	X		483.8			
69	ZYQ55004	X		598			
70	ZYP15903	X		547.7			
74	ZYK07703	X		866.2			
76	ZYA77703	X		544.4			
81	YVK66802	X		372.4			
82	YVH98502	X		708.7			
86	YTH39103	X		554.5			
87	YRK12903	X		1057			
89	YVN56803	X		873.3			
90	YXG54802	X		97.3			
91	YXB86803	X		576.6			
92	YXB17502	X		449.7			
93	YYP28903	X		327.6			
94	YXI77903	X		663.3			
95	YXH13902	X		883.6			
96	YVQ30502	X		38.0			
98	YVO25503	X		179.9			
99	YWY92203	X		595			
100	YWS77103	X		583.6			
101	YWE11002	X		513.8			
102	UAH60948	X		900.6			
103	UAH60698	X		1508			
104	UAH60984	X		135.3			
105	UAH60991	X		332.7			
106	UAH60986	X		246			
107	UAH60296	X		55.0			
108	UAH60289	X		51.0			
109	UAH60275	X		368.6			
110	UAH60290	X		1056			
111	UAH60292	X		117.3			
112	UAH60291	X		139.8			
113	UAH60992	X		198.1			
114	UAH61011	X		258.3			
115	UAH61010	X		108.9			
116	UAH61012	X		80.6			
117	UAH61094	X		391.5			
118	UAH61017	X		720.5			
119	UAH61009	X		265.3			
120	UAH61005	X		156.5			
121	UAH60993	X		111.2			
122	UAH61006	X		85.2			
123	UAH61008	X		330.5			
124	UAH61007	X		222.6			
125	UAH60268	X		135.2			
126	ABX89603	X		468.9			
127	AAO89903	X		991.2			
128	ACE32303	X		711.4			
129	ACK91202	X		607.8			
130	ACK08903	X		900.1			
131	31298365	X		891.5			
132	31136348	X		336.8			
134	31137166	X		316.6			
135	31272306	X		744.9			
137	ACU43302	X		705.7			
138	UAH60216	X		98.5			
139	UAH59327	X		1452			
140	UAH60234	X		491.9			
141	UAH60264	X		662.6			
142	UAH60242	X		513.4			
143	TRG37204	X		1261			
144	AEZ70303	X		1052			
145	AEJ98903	X		969.5			
146	TNJ27502	X		1041			
147	TPJ51503	X		1037			
148	TNM00102	X		1013			
174	UAH61137	X		475.7			
175	UAH61127	X		280.4			
176	UAH61156	X		357			
177	UAH61147	X		605.3			
178	UAH61109	X		366			
179	UAH61100	X		414			
180	UAH61123	X		472.1			
181	UAH61121	X		364.5			
182	UAH61174	X		168.1			
183	UAH61212	X		967.7			
185	UAH61177		38	48.8	10.8	-17.36	0.7
186	120029281		202	215.5	13.5	200.99	0.2
187	UAH61003		241.8	272.8	31.0	253.98	0.2
188	UAH61172		287	319.9	32.9	314.16	0.1
189	UAH61169		304.9	395.5	90.6	337.99	0.6
190	UAH61151		406	500.9	94.9	472.60	0.3
191	UAH60985		418.5	399.9	-18.6	489.24	-1.0
192	UAH61166		419	481.2	62.2	489.91	-0.1
193	UAH61096		500.5	618.4	117.9	598.42	0.2
194	UAH61148		519	668.8	149.8	623.05	0.5
195	UAH61165		532	610.8	78.8	640.36	-0.3
196	ZYO00403		566	709.2	143.2	685.62	0.3
197	UAH61034		682.1	970.1	288.0	840.20	1.4
198	UAH60996		703.2	771.9	68.7	868.29	-1.1
199	YXI44303		781.8	906.1	124.3	972.94	-0.7
200	UAH61162		802	870.5	68.5	999.84	-1.4
201	UAH61176		820	903.3	83.3	1023.80	-1.3
202	3037895		890.8	1067	176.2	1118.07	-0.6
203	UAH61256		917.1	1096	178.9	1153.08	-0.6
204	TNM23403		967.7	1459	491.3	1220.45	2.7

Values marked with an "X" were excluded from the calculations. Outliers "O" were also excluded.

Copyright 1991-2005 David G. Rhoads Associates, Inc.

Alternate (Quantitative) Method Comparison

X Method: LC/MSMS **Y Method: e411**

Scatter Plot

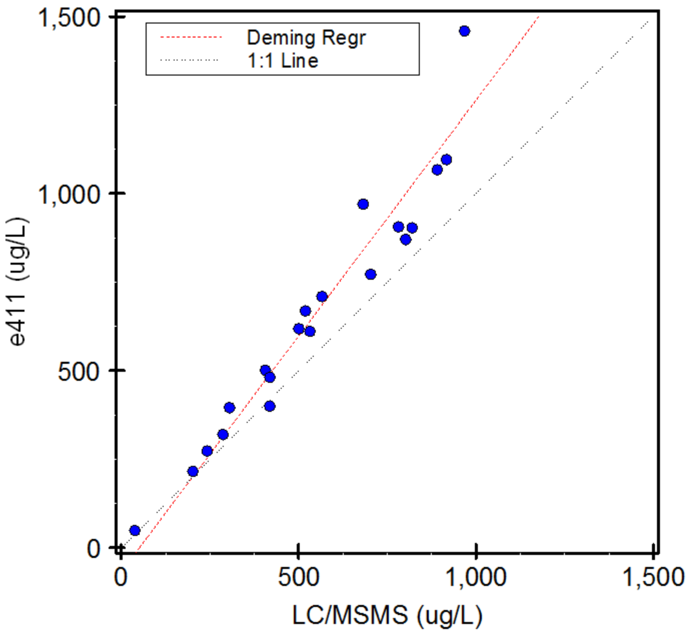

Bias

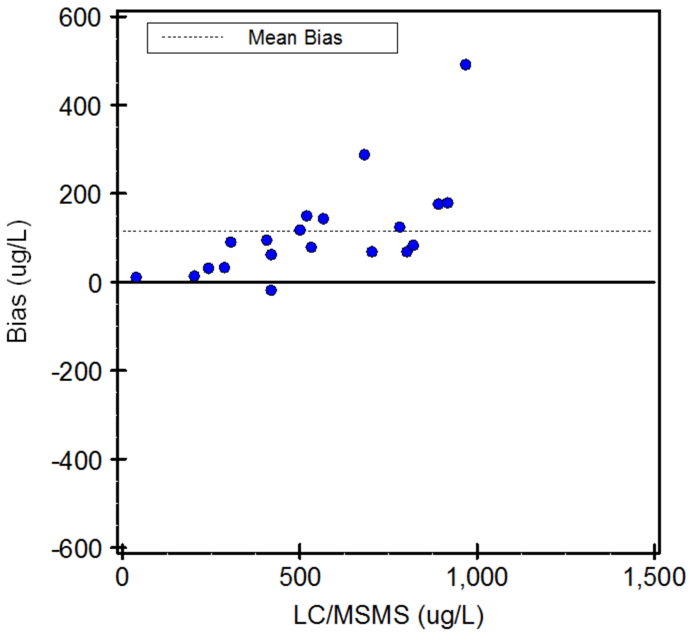

Percent Bias

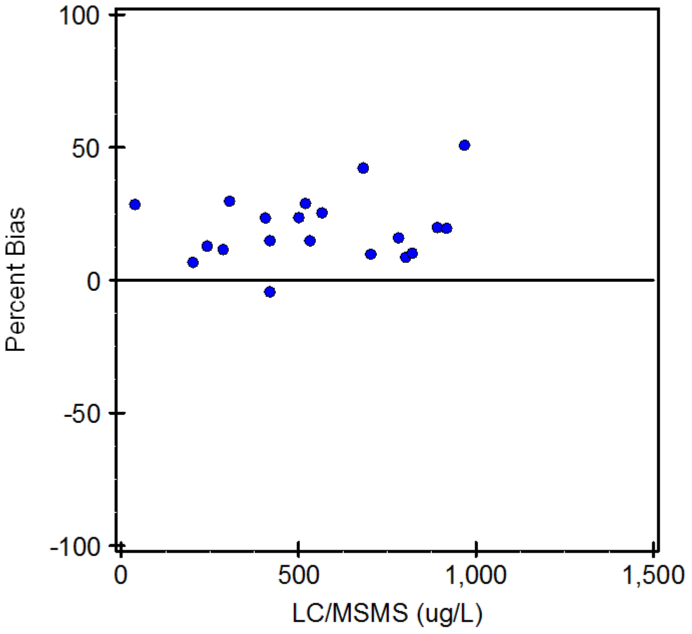

This page contains a larger, working copy of the same graphs that appear on page 1.

Copyright 1991-2005 David G. Rhoads Associates, Inc.

Alternate (Quantitative) Method Comparison

X Method: LC/MSMS **Y Method: e411**

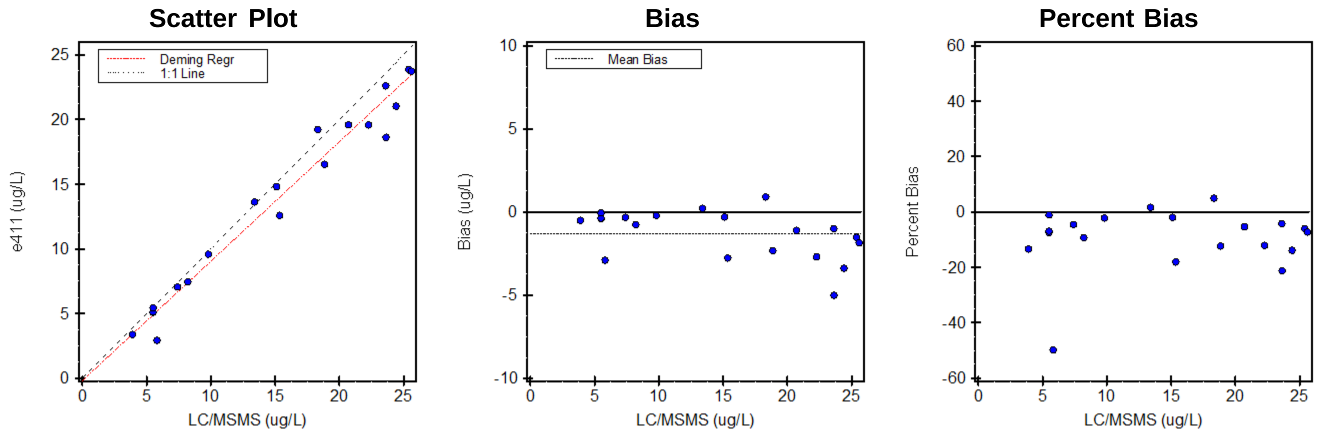

Regression Analysis

	Deming	Regular
**Slope:**	0.924 (0.843 to 1.005)	0.910 (0.830 to 0.991)
**Intercept:**	-0.19 (-1.54 to 1.17)	0.01 (-1.34 to 1.36)
**Std Err Est:**	1.32	1.32

95% Confidence Intervals are shown in parentheses

Supporting Statistics

Corr Coef (R):	0.9844	SubRange Bounds:	None
Bias:	-1.32	Points (Plotted/Total):	20/100
X Mean ± SD:	14.91 ± 7.88	Outliers:	Not Tested
Y Mean ± SD:	13.59 ± 7.29	Scatter Plot Bounds:	None
Std Dev Diffs:	1.46		

Experiment Description

	X Method	Y Method
Expt Date:	27 May 2015	27 May 2015
Rep SD:	1	1
Result Ranges:	3.9 to 25.6	2.9 to 23.9
Units:	ug/L	ug/L
Analyst:	MK	MK
Comment:		

Accepted by: ______________________________ ______________

Signature Date

Copyright 1991-2005 David G. Rhoads Associates, Inc.

Alternate (Quantitative) Method Comparison

X Method: LC/MSMS **Y Method: e411**

Experimental Results

	Spec ID	Results X	Results Y	Bias	Calc'd Y	SEE Factor
1	UAJ6452	X	1.6			
2	UAJ6451	X	8.6			
3	UAJ6453	X	11.4			
4	UAJ6456	X	5.4			
5	UAJ6454	X	12.1			
6	UAJ6450	X	8.0			
7	UAJ6444	X	2.5			
8	UAJ6443	X	5.3			
9	UAJ6445	X	10.4			
10	UAJ6447	X	6.0			
11	UAJ6446	X	7.2			
12	UAJ6458	X	2.5			
14	UAJ6472	X	2.7			
15	UAJ6488	X	5.1			
16	ZSS39603	X	18.9			
17	UAJ6745	X	15.4			
18	UAJ6469	X	13.0			
19	UAJ6461	X	7.6			
20	UAJ6460	X	12.1			
21	UAJ6464	X	19.7			
22	UAJ6468	X	3.8			
23	UAJ6467	X	11.1			
24	UAJ6419	X	11.3			
25	UAJ6412	X	7.2			
29	UAJ6410	X	7.5			
32	UAJ6401	X	6.5			
33	UAJ6404	X	9.8			
34	UAJ6402	X	9.3			
35	UAJ6426	X	18.7			
36	UAJ6439	X	7.3			
37	UAJ6438	X	13.3			
38	UAJ6440	X	12.6			
39	UAJ6442	X	7.5			
40	UAJ6441	X	6.1			
42	UAJ6428	X	5.6			
43	UAJ6427	X	14.8			
44	UAJ6429	X	7.5			
45	UAJ6434	X	11.2			
46	UAJ6430	X	17.9			
54	YVP40704	X	10.3			
59	ZIY67902	X	3.5			
60	ZIQ08702	X	13.1			
61	ZJA38902	X	12.3			
62	ZJM20004	X	9.3			
63	ZJE32203	X	2.9			
64	ZIO88003	X	2.0			
65	YZY99904	X	18.5			
66	YYS62303	X	20.8			
68	ZIM40003	X	11.8			
70	UAJ7025	X	16.0			
71	UAJ6916	X	17.8			
72	ZRG65302	X	16.3			
73	YQX43302	X	7.4			
74	UAJ7055	X	20.2			
75	UAJ6903	X	15.2			
76	UAJ6802	X	31.8			
77	UAJ6794	X	17.2			
78	UAJ6807	X	25.1			
79	UAJ6902	X	17.2			
80	UAJ6853	X	13.6			
81	YRA78903	X	15.1			
82	YVH98503	X	9.6			
87	YVH85302	X	6.3			
91	ZKB4603	X	12.3			
92	ZQX95203	X	14.0			
124	31113015	X	14.9			
127	31197733	X	3.0			
142	UAJ5620	X	8.5			
143	UAJ5607	X	25.2			
144	UAJ5679	X	19.1			
145	UAJ5736	X	22.5			
146	UAJ5728	X	29.8			
153	UAJ5753	X	5.9			
154	UAJ6014	X	18.7			
157	UAJ6149	X	14.3			
159	UAJ5977	X	18.3			
160	UAJ5763	X	3.9			
161	UAJ5755	X	14.1			
162	UAJ5792	X	19.8			
164	UAJ5794	X	12.2			
186	UAJ6449	3.9	3.4	-0.5	3.42	0.0
187	UAJ6424	5.5	5.1	-0.4	4.89	0.2
188	UAJ6448	5.5	5.4	-0.1	4.89	0.4
189	UAJ6409	5.5	5.1	-0.4	4.89	0.1
190	YVM26903	5.8	2.9	-2.9	5.19	-1.7
191	UAJ6421	7.4	7.1	-0.3	6.65	0.3
192	UAJ6422	8.2	7.4	-0.8	7.39	0.0
193	UAJ6423	9.8	9.6	-0.2	8.87	0.5
194	UAJ6436	13.4	13.6	0.2	12.19	1.1
195	YUZ03304	15.1	14.8	-0.3	13.77	0.8
196	UAJ6475	15.3	12.6	-2.8	13.99	-1.1
197	UAJ5808	18.3	19.2	0.9	16.74	1.9
198	UAJ6185	18.9	16.5	-2.3	17.23	-0.5
199	UAJ7049	20.7	19.6	-1.1	18.93	0.5
200	UAJ6009	22.3	19.6	-2.7	20.38	-0.6
201	ZUH12003	23.6	22.6	-1.0	21.61	0.7
202	UAJ6151	23.6	18.6	-5.0	21.64	-2.3
203	UAJ6030	24.4	21.0	-3.4	22.36	-1.0
204	YVA63003	25.4	23.9	-1.5	23.27	0.4
205	UAJ6755	25.6	23.7	-1.9	23.45	0.2

Values marked with an "X" were excluded from the calculations.

Copyright 1991-2005 David G. Rhoads Associates, Inc.

Alternate (Quantitative) Method Comparison

X Method: LC/MSMS **Y Method: e411**

Scatter Plot

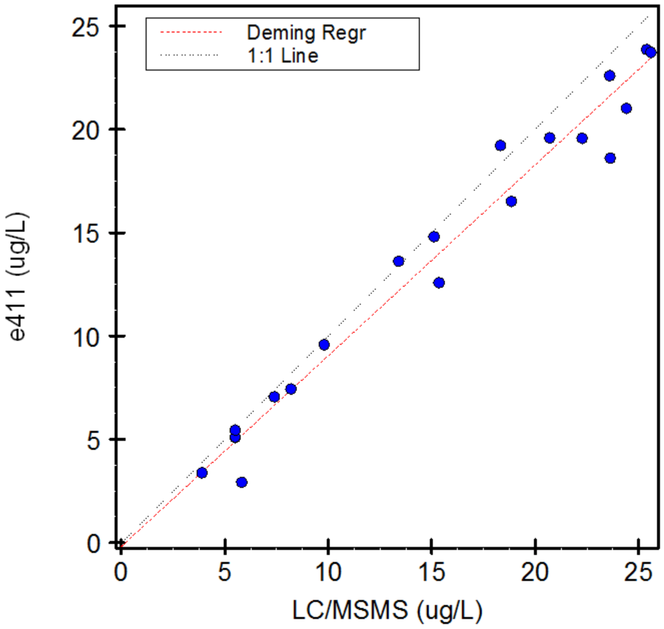

Bias

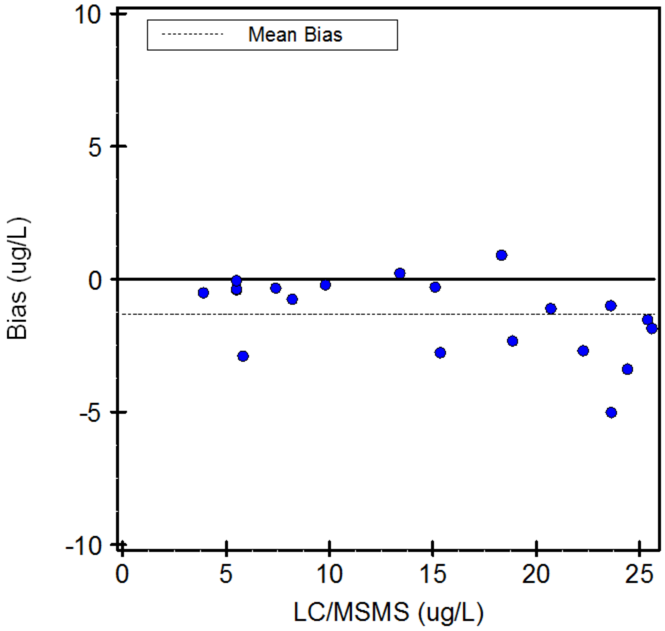

Percent Bias

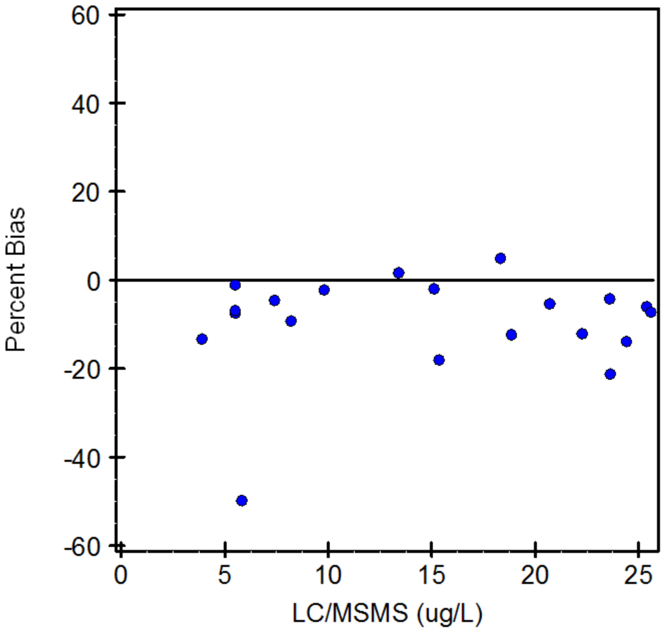

This page contains a larger, working copy of the same graphs that appear on page 1.

Copyright 1991-2005 David G. Rhoads Associates, Inc.

Alternate (Quantitative) Method Comparison

X Method: e411 **Y Method: e411(lot2)**

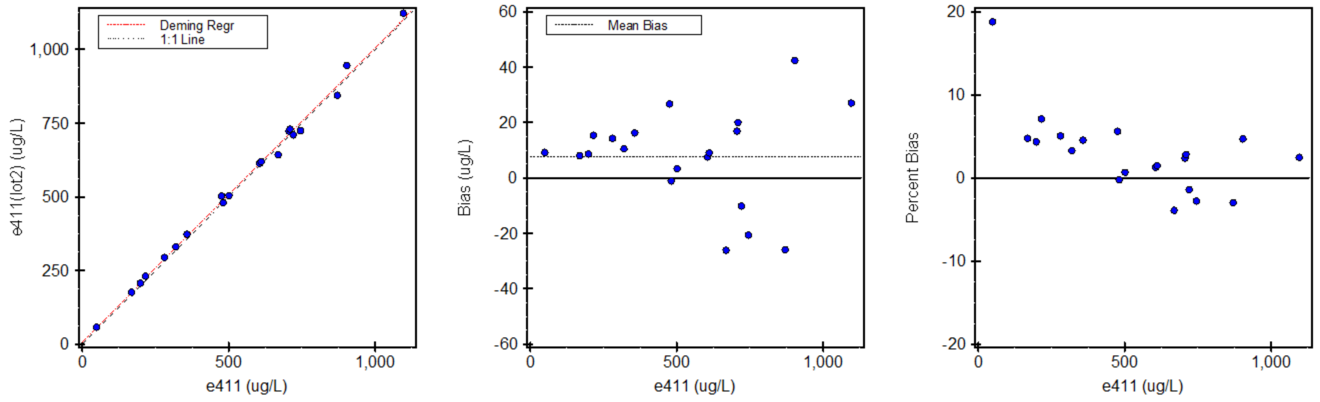

Regression Analysis

	Deming	Regular
**Slope:**	0.998 (0.967 to 1.029)	0.996 (0.965 to 1.027)
**Intercept:**	8.48 (-10.22 to 27.17)	9.54 (-9.15 to 28.22)
**Std Err Est:**	18.00	17.99

95% Confidence Intervals are shown in parentheses

Supporting Statistics

Corr Coef (R):	0.9980	SubRange Bounds:	None
Bias:	7.55	Points (Plotted/Total):	20/100
X Mean ± SD:	534.03 ± 277.81	Outliers:	None
Y Mean ± SD:	541.58 ± 277.33	Scatter Plot Bounds:	None
Std Dev Diffs:	17.54		

Experiment Description

	X Method	Y Method
Expt Date:	27 May 2015	27 May 2015
Rep SD:	1	1
Result Ranges:	48.8 to 1096.0	58.0 to 1123.0
Units:	ug/L	ug/L
Analyst:	MK	MK
Comment:		

Accepted by: ______________________ ____________

Signature Date

Copyright 1991-2005 David G. Rhoads Associates, Inc.

Alternate (Quantitative) Method Comparison

X Method: e411 **Y Method: e411(lot2)**

Experimental Results

	Spec ID		Results X	Results Y	Bias	Calc'd Y	SEE Factor
105	YVQ30502	X	38.0				
106	UAH61177		48.8	58.0	9.2	57.21	0.0
107	UAH60289	X	51.0				
108	UAH60296	X	55.0				
109	UAH61012	X	80.6				
110	UAH61006	X	85.2				
111	YXG54802	X	97.3				
112	UAH60216	X	98.5				
113	UAH61010	X	108.9				
114	UAH60993	X	111.2				
115	UAH60292	X	117.3				
116	UAH60268	X	135.2				
117	UAH60984	X	135.3				
118	UAH60291	X	139.8				
119	UAH61005	X	156.5				
120	UAH61174		168.1	176.1	8.0	176.29	0.0
121	YVO25503	X	179.9				
122	UAH60992		198.1	206.7	8.6	206.23	0.0
123	120029281		215.5	230.8	15.3	223.60	0.4
124	UAH61007	X	222.6				
125	UAH60986	X	246				
126	UAH61011	X	258.3				
127	UAH61009	X	265.3				
128	UAH61003	X	272.8				
129	UAH61127		280.4	294.6	14.2	288.39	0.3
130	31137166	X	316.6				
131	UAH61172		319.9	330.4	10.5	327.82	0.1
132	YYP28903	X	327.6				
133	UAH61008	X	330.5				
134	UAH60991	X	332.7				
135	31136348	X	336.8				
136	UAH61156		357	373.2	16.2	364.86	0.5
137	UAH61121	X	364.5				
138	UAH61109	X	366				
139	UAH60275	X	368.6				
140	YVK66802	X	372.4				
141	UAH61094	X	391.5				
142	UAH61169	X	395.5				
143	UAH60985	X	399.9				
144	UAH61100	X	414				
145	YXB17502	X	449.7				
146	ABX89603	X	468.9				
147	UAH61123	X	472.1				
148	UAH61137		475.7	502.4	26.7	483.35	1.1
149	UAH61166		481.2	480.1	-1.1	488.84	-0.5
150	ZYQ85102	X	483.8				
151	UAH60234	X	491.9				
152	UAH61151		500.9	504.2	3.3	508.51	-0.2
153	UAH60242	X	513.4				
154	YWE11002	X	513.8				

	Spec ID		Results X	Results Y	Bias	Calc'd Y	SEE Factor
155	ZYA77703	X	544.4				
156	ZYP15903	X	547.7				
157	YTH39103	X	554.5				
158	YXB86803	X	576.6				
159	YWS77103	X	583.6				
160	YWY92203	X	595				
161	ZYQ55004	X	598				
162	UAH61147		605.3	612.9	7.6	612.73	0.0
163	ACK91202	X	607.8				
164	UAH61165		610.8	619.8	9.0	618.22	0.1
165	UAH61096	X	618.4				
166	UAH60264	X	662.6				
167	YXI77903	X	663.3				
168	UAH61148		668.8	642.7	-26.1	676.12	-1.9
169	ACU43302		705.7	722.6	16.9	712.96	0.5
170	YVH98502	X	708.7				
171	ZYO00403		709.2	729.2	20.0	716.45	0.7
172	ACE32303	X	711.4				
173	UAH61017		720.5	710.4	-10.1	727.73	-1.0
174	ZYO78603	X	724.7				
175	31272306		744.9	724.3	-20.6	752.09	-1.5
176	ZYN23003	X	746.7				
177	UAH60996	X	771.9				
178	ZYK07703	X	866.2				
179	UAH61162		870.5	844.6	-25.9	877.47	-1.8
180	YVN56803	X	873.3				
181	YXH13902	X	883.6				
182	31298365	X	891.5				
183	ACK08903	X	900.1				
184	UAH60948	X	900.6				
185	UAH61176		903.3	945.7	42.4	910.21	2.0
186	YXI44303	X	906.1				
187	UAH61212	X	967.7				
188	AEJ98903	X	969.5				
189	UAH61034	X	970.1				
190	AAO89903	X	991.2				
191	TNM00102	X	1013				
192	TPJ51503	X	1037				
193	TNJ27502	X	1041				
194	AEZ70303	X	1052				
195	UAH60290	X	1056				
196	YRK12903	X	1057				
197	3037895	X	1067				
198	UAH61256		1096	1123	27	1102.58	1.1
199	ZFH97703	X	1197				
200	TRG37204	X	1261				
201	UAH59327	X	1452				
202	TNM23403	X	1459				
203	ZYN41003	X	1464				
204	UAH60698	X	1508				

Values marked with an "X" were excluded from the calculations. Outliers "O" were also excluded.

Copyright 1991-2005 David G. Rhoads Associates, Inc.

Alternate (Quantitative) Method Comparison

X Method: e411 **Y Method: e411(lot2)**

Scatter Plot

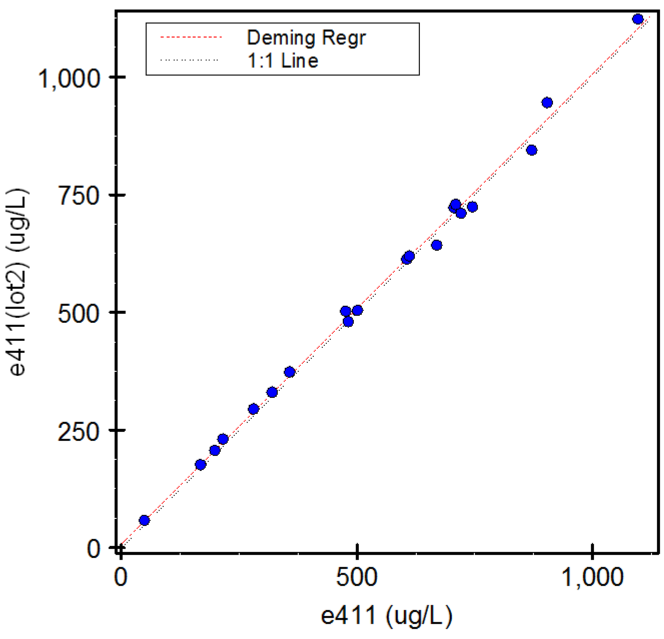

Bias

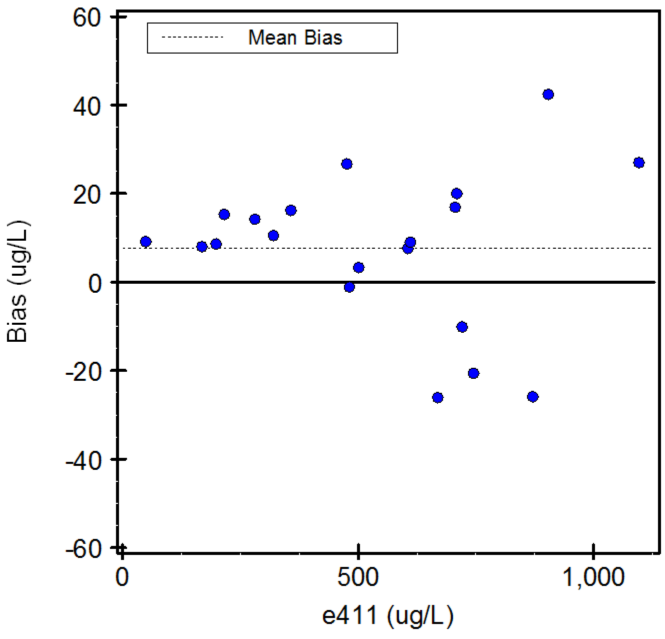

Percent Bias

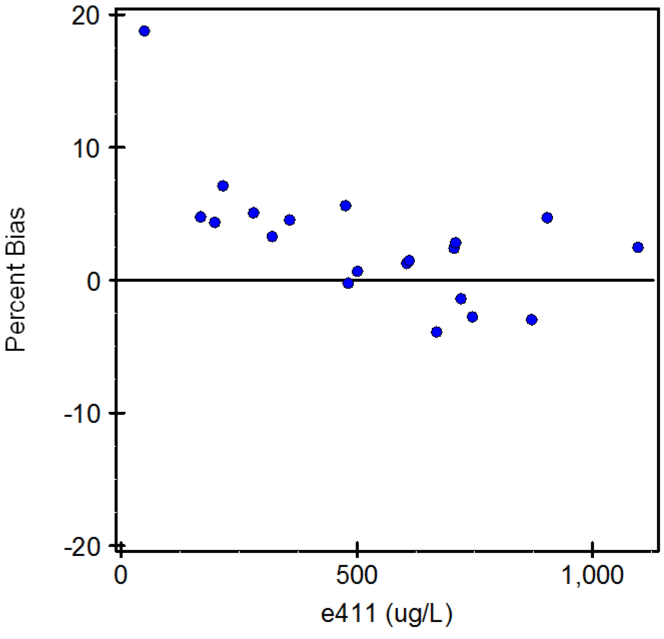

This page contains a larger, working copy of the same graphs that appear on page 1.

Copyright 1991-2005 David G. Rhoads Associates, Inc.

Alternate (Quantitative) Method Comparison

X Method: e411 **Y Method: e411(lot2)**

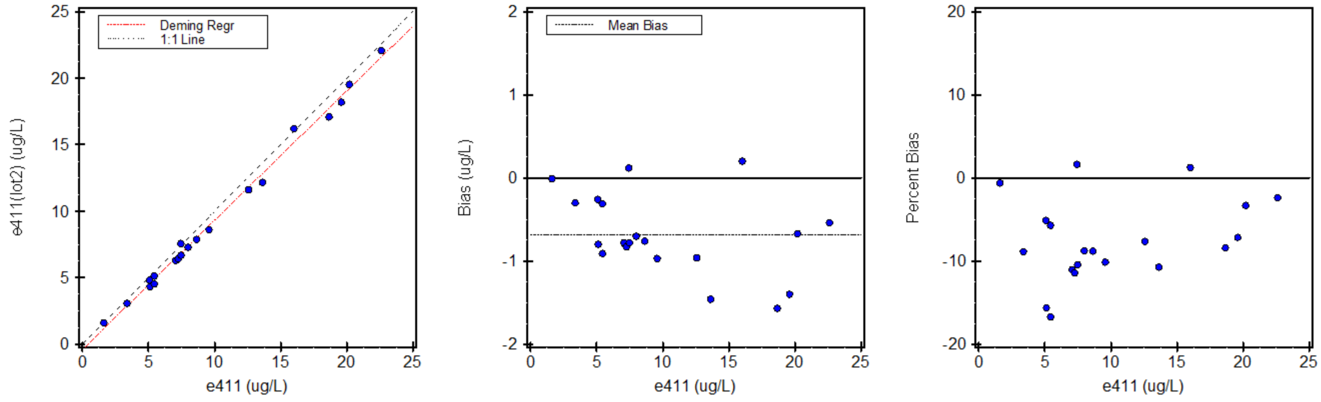

Regression Analysis

	Deming	Regular
**Slope:**	0.972 (0.936 to 1.008)	0.970 (0.933 to 1.006)
**Intercept:**	-0.40 (-0.83 to 0.03)	-0.37 (-0.80 to 0.06)
**Std Err Est:**	0.46	0.46

95% Confidence Intervals are shown in parentheses

Supporting Statistics

Corr Coef (R):	0.9972	SubRange Bounds:	None
Bias:	-0.68	Points (Plotted/Total):	20/100
X Mean ± SD:	10.24 ± 6.17	Outliers:	None
Y Mean ± SD:	9.55 ± 6.00	Scatter Plot Bounds:	None
Std Dev Diffs:	0.49		

Experiment Description

	X Method	Y Method
Expt Date:	27 May 2015	27 May 2015
Rep SD:	1	1
Result Ranges:	1.6 to 22.6	1.6 to 22.1
Units:	ug/L	ug/L
Analyst:	MK	MK
Comment:		

Accepted by: ______________________ ______________

Signature Date

Copyright 1991-2005 David G. Rhoads Associates, Inc.

Alternate (Quantitative) Method Comparison

X Method: e411 **Y Method: e411(lot2)**

Experimental Results

	Spec ID		Results X	Results Y	Bias	Calc'd Y	SEE Factor
106	UAJ6452		1.6	1.6	0.0	1.18	0.9
107	ZIO88003	X	2.0				
108	UAJ6458	X	2.5				
109	UAJ6444	X	2.5				
110	UAJ6472	X	2.7				
111	ZJE32203	X	2.9				
112	YVM26903	X	2.9				
113	31197733	X	3.0				
114	UAJ6449		3.4	3.1	-0.3	2.89	0.4
115	ZIY67902	X	3.5				
116	UAJ6468	X	3.8				
117	UAJ5763	X	3.9				
118	UAJ6409		5.1	4.8	-0.3	4.55	0.6
119	UAJ6488	X	5.1				
120	UAJ6424		5.1	4.3	-0.8	4.58	-0.6
121	UAJ6443	X	5.3				
122	UAJ6456		5.4	5.1	-0.3	4.88	0.5
123	UAJ6448		5.4	4.5	-0.9	4.89	-0.8
124	UAJ6428	X	5.6				
125	UAJ5753	X	5.9				
126	UAJ6447	X	6.0				
127	UAJ6441	X	6.1				
128	YVH85302	X	6.3				
129	UAJ6401	X	6.5				
130	UAJ6421		7.1	6.3	-0.8	6.46	-0.4
131	UAJ6446	X	7.2				
132	UAJ6412	X	7.2				
133	UAJ6439		7.3	6.4	-0.8	6.66	-0.5
134	YQX43302	X	7.4				
135	UAJ6422		7.4	7.6	0.1	6.83	1.6
136	UAJ6429	X	7.5				
137	UAJ6442		7.5	6.7	-0.8	6.86	-0.4
138	UAJ6410	X	7.5				
139	UAJ6461	X	7.6				
140	UAJ6450		8.0	7.3	-0.7	7.37	-0.2
141	UAJ5620	X	8.5				
142	UAJ6451		8.6	7.9	-0.8	8.00	-0.3
143	UAJ6402	X	9.3				
144	ZJM20004	X	9.3				
145	YVH98503	X	9.6				
146	UAJ6423		9.6	8.6	-1.0	8.91	-0.7
147	UAJ6404	X	9.8				
148	YVP40704	X	10.3				
149	UAJ6445	X	10.4				
150	UAJ6467	X	11.1				
151	UAJ6434	X	11.2				
152	UAJ6419	X	11.3				
153	UAJ6453	X	11.4				
154	ZIM40003	X	11.8				
155	UAJ6460	X	12.1				

	Spec ID		Results X	Results Y	Bias	Calc'd Y	SEE Factor
156	UAJ6454	X	12.1				
157	UAJ5794	X	12.2				
158	ZKB4603	X	12.3				
159	ZJA38902	X	12.3				
160	UAJ6440		12.6	11.6	-1.0	11.82	-0.5
161	UAJ6475	X	12.6				
162	UAJ6469	X	13.0				
163	ZIQ08702	X	13.1				
164	UAJ6438	X	13.3				
165	UAJ6853	X	13.6				
166	UAJ6436		13.6	12.2	-1.5	12.84	-1.5
167	ZQX95203	X	14.0				
168	UAJ5755	X	14.1				
169	UAJ6149	X	14.3				
170	YUZ03304	X	14.8				
171	UAJ6427	X	14.8				
172	31113015	X	14.9				
173	YRA78903	X	15.1				
174	UAJ6903	X	15.2				
175	UAJ6745	X	15.4				
176	UAJ7025		16.0	16.2	0.2	15.17	2.3
177	ZRG65302	X	16.3				
178	UAJ6185	X	16.5				
179	UAJ6794	X	17.2				
180	UAJ6902	X	17.2				
181	UAJ6916	X	17.8				
182	UAJ6430	X	17.9				
183	UAJ5977	X	18.3				
184	YZY99904	X	18.5				
185	UAJ6151	X	18.6				
186	UAJ6426		18.7	17.1	-1.6	17.74	-1.4
187	UAJ6014	X	18.7				
188	ZSS39603	X	18.9				
189	UAJ5679	X	19.1				
190	UAJ5808	X	19.2				
191	UAJ6009	X	19.6				
192	UAJ7049		19.6	18.2	-1.4	18.65	-1.0
193	UAJ6464	X	19.7				
194	UAJ5792	X	19.8				
195	UAJ7055		20.2	19.5	-0.7	19.24	0.6
196	YYS62303	X	20.8				
197	UAJ6030	X	21.0				
198	UAJ5736	X	22.5				
199	ZUH12003		22.6	22.1	-0.5	21.57	1.1
200	UAJ6755	X	23.7				
201	YVA63003	X	23.9				
202	UAJ6807	X	25.1				
203	UAJ5607	X	25.2				
204	UAJ5728	X	29.8				
205	UAJ6802	X	31.8				

Values marked with an "X" were excluded from the calculations. Outliers "O" were also excluded.

Copyright 1991-2005 David G. Rhoads Associates, Inc.

Alternate (Quantitative) Method Comparison

X Method: e411 **Y Method: e411(lot2)**

Scatter Plot

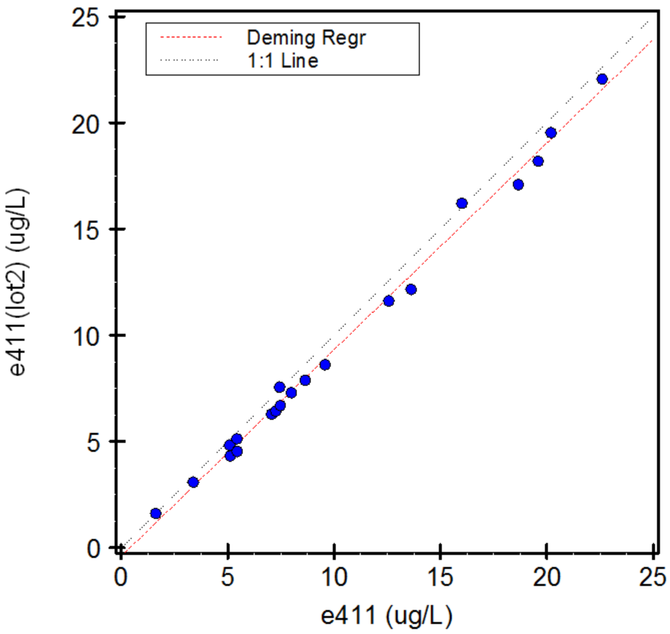

Bias

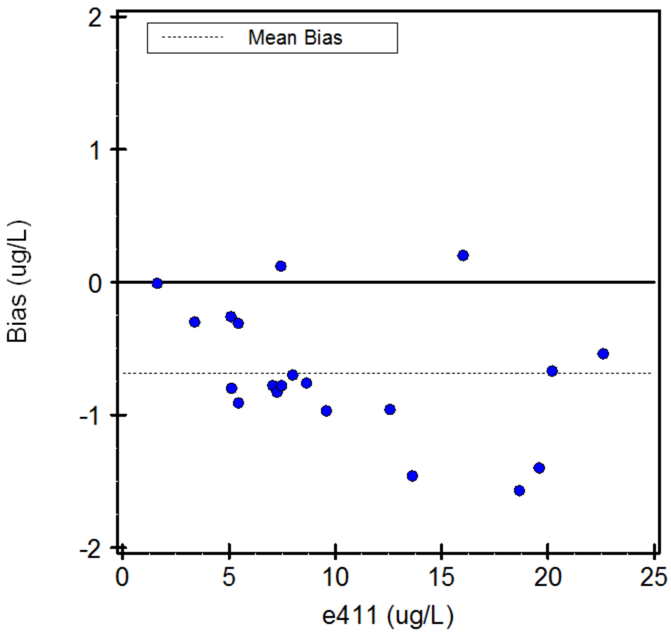

Percent Bias

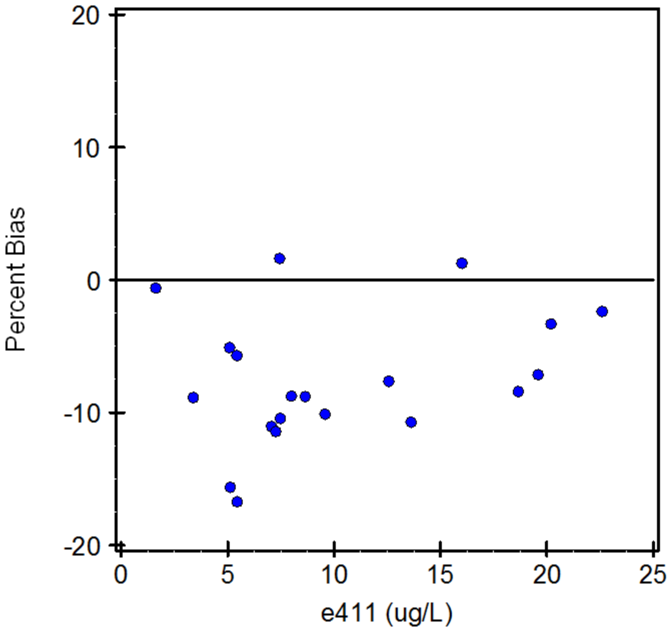

This page contains a larger, working copy of the same graphs that appear on page 1.

Copyright 1991-2005 David G. Rhoads Associates, Inc.

Alternate (Quantitative) Method Comparison

X Method: LC/MSMS **Y Method: i2000**

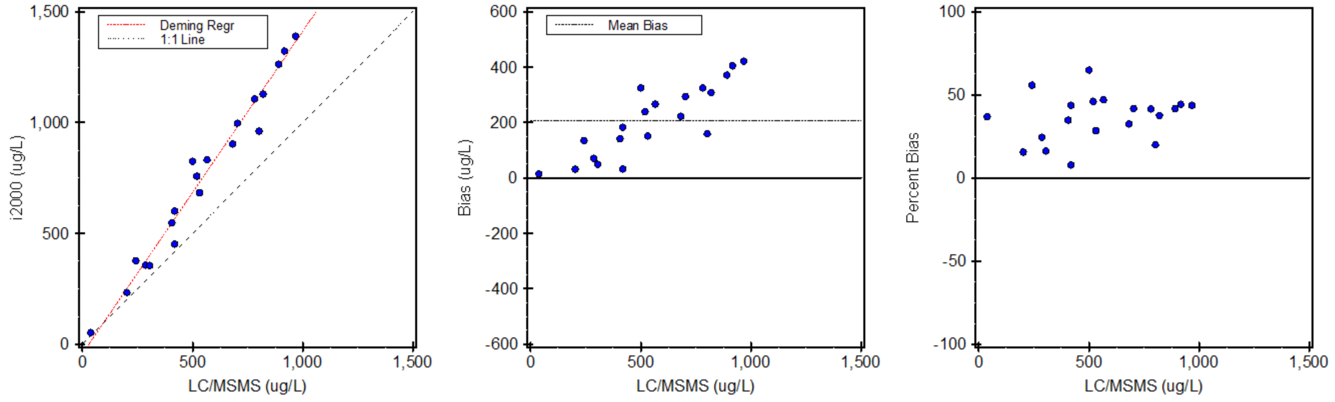

Regression Analysis

	Deming	Regular
**Slope:**	1.454 (1.332 to 1.577)	1.426 (1.304 to 1.547)
**Intercept:**	-42.84 (-117.22 to 31.54)	-27.13 (-101.01 to 46.76)
**Std Err Est:**	67.07	66.62

95% Confidence Intervals are shown in parentheses

Supporting Statistics

Corr Coef (R):	0.9855	SubRange Bounds:	None
Bias:	206.99	Points (Plotted/Total):	20/100
X Mean ± SD:	549.97 ± 263.89	Outliers:	None
Y Mean ± SD:	756.96 ± 381.77	Scatter Plot Bounds:	None
Std Dev Diffs:	129.71		

Experiment Description

	X Method	Y Method
Expt Date:	27 May 2015	27 May 2015
Rep SD:	1	1
Result Ranges:	38.0 to 967.7	52.0 to 1389.0
Units:	ug/L	ug/L
Analyst:	MK	MK
Comment:		

Accepted by: ______________________________ ______________

Signature Date

Copyright 1991-2005 David G. Rhoads Associates, Inc.

Alternate (Quantitative) Method Comparison

X Method: LC/MSMS **Y Method: i2000**

Experimental Results

	Spec ID		Results X	Results Y	Bias	Calc'd Y	SEE Factor
63	ZFH97703	X		1283			
65	ZYO78603	X		710			
66	ZYN41003	X		1808			
67	ZYN23003	X		770.3			
68	ZYQ85102	X		613.2			
69	ZYQ55004	X		691.4			
70	ZYP15903	X		615.5			
74	ZYK07703	X		953.8			
76	ZYA77703	X		627.8			
81	YVK66802	X		403			
82	YVH98502	X		786			
86	YTH39103	X		710			
87	YRK12903	X		1101			
89	YVN56803	X		999			
90	YXG54802	X		101			
91	YXB86803	X		730			
92	YXB17502	X		447			
93	YYP28903	X		381			
94	YXI77903	X		802			
95	YXH13902	X		876			
96	YVQ30502	X		41			
98	YVO25503	X		217			
99	YWY92203	X		604			
100	YWS77103	X		699			
101	YWE11002	X		554			
102	UAH60948	X		1058.3			
103	UAH60698	X		1624			
104	UAH60984	X		201.9			
105	UAH60991	X		393.6			
106	UAH60986	X		335.4			
107	UAH60296	X		52			
108	UAH60289	X		63			
109	UAH60275	X		419			
110	UAH60290	X		1040			
111	UAH60292	X		123			
112	UAH60291	X		135			
113	UAH60992	X		250.3			
114	UAH61011	X		348.3			
115	UAH61010	X		129.7			
116	UAH61012	X		101.4			
117	UAH61094	X		437.7			
118	UAH61017	X		853.2			
119	UAH61009	X		355.1			
120	UAH61005	X		196.5			
121	UAH60993	X		129.5			
122	UAH61006	X		93.1			
123	UAH61008	X		421.1			
124	UAH61007	X		286.7			
125	UAH60268	X		166			
126	ABX89603	X		473.9			
127	AAO89903	X		1026.4			
128	ACE32303	X		680.9			
129	ACK91202	X		586.2			
130	ACK08903	X		955.7			
131	31298365	X		1008.2			
132	31136348	X		374			
134	31137166	X		415			
135	31272306	X		934.6			
137	ACU43302	X		771.3			
138	UAH60216	X		106			
139	UAH59327	X		1696			
140	UAH60234	X		563			
141	UAH60264	X		681			
142	UAH60242	X		571			
143	TRG37204	X		1231			
144	AEZ70303	X		1071.1			
145	AEJ98903	X		1089.7			
146	TNJ27502	X		1080			
147	TPJ51503	X		1138			
148	TNM00102	X		1150			
174	UAH61137	X		595.9			
175	UAH61127	X		312			
176	UAH61156	X		444.6			
177	UAH61147	X		562.1			
178	UAH61109	X		428.7			
179	UAH61100	X		503.4			
180	UAH61123	X		557.6			
181	UAH61121	X		443.1			
182	UAH61174	X		168.4			
183	UAH61212	X		1046.6			
185	UAH61177		38	52	14	12.42	0.6
186	120029281		202	233.4	31.4	250.92	-0.3
187	UAH61003		241.8	376.7	134.9	308.80	1.0
188	UAH61172		287	357.1	70.1	374.53	-0.3
189	UAH61169		304.9	354	49.1	400.56	-0.7
190	UAH61151		406	547.1	141.1	547.59	0.0
191	UAH60985		418.5	601.1	182.6	565.77	0.5
192	UAH61166		419	451.5	32.5	566.50	-1.7
193	UAH61096		500.5	825.1	324.6	685.02	2.1
194	UAH61148		519	757.8	238.8	711.92	0.7
195	UAH61165		532	683	151	730.83	-0.7
196	ZYO00403		566	831.8	265.8	780.27	0.8
197	UAH61034		682.1	903.9	221.8	949.11	-0.7
198	UAH60996		703.2	996.6	293.4	979.80	0.3
199	YXI44303		781.8	1106	324.2	1094.10	0.2
200	UAH61162		802	961.4	159.4	1123.48	-2.4
201	UAH61176		820	1127.7	307.7	1149.65	-0.3
202	3037895		890.8	1262	371.2	1252.62	0.1
203	UAH61256		917.1	1322	404.9	1290.86	0.5
204	TNM23403		967.7	1389	421.3	1364.45	0.4

Values marked with an "X" were excluded from the calculations. Outliers "O" were also excluded.

Copyright 1991-2005 David G. Rhoads Associates, Inc.

Alternate (Quantitative) Method Comparison

X Method: LC/MSMS **Y Method: i2000**

Scatter Plot

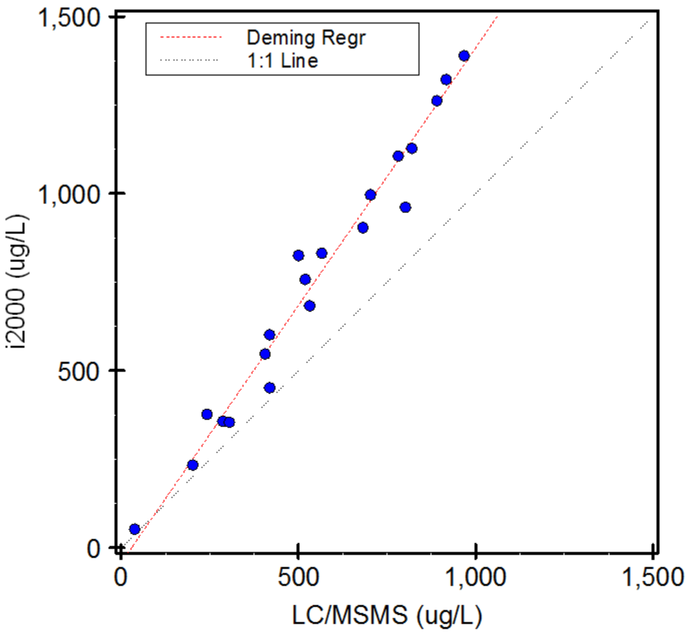

Bias

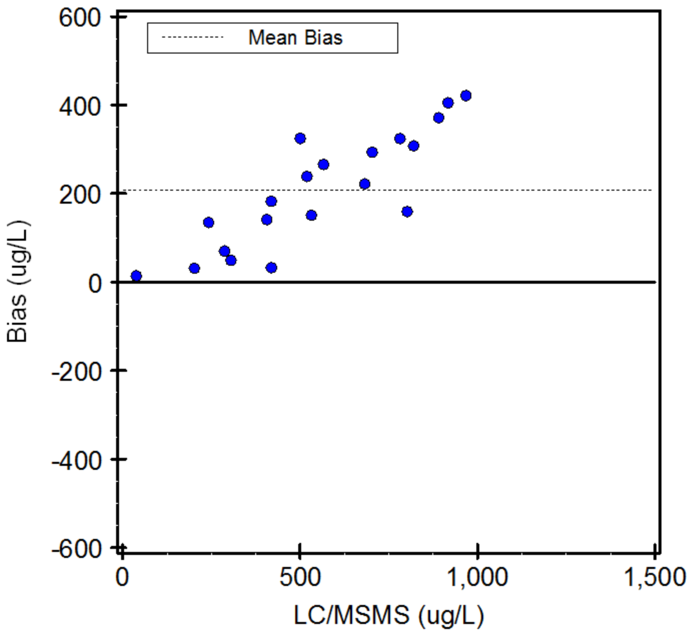

Percent Bias

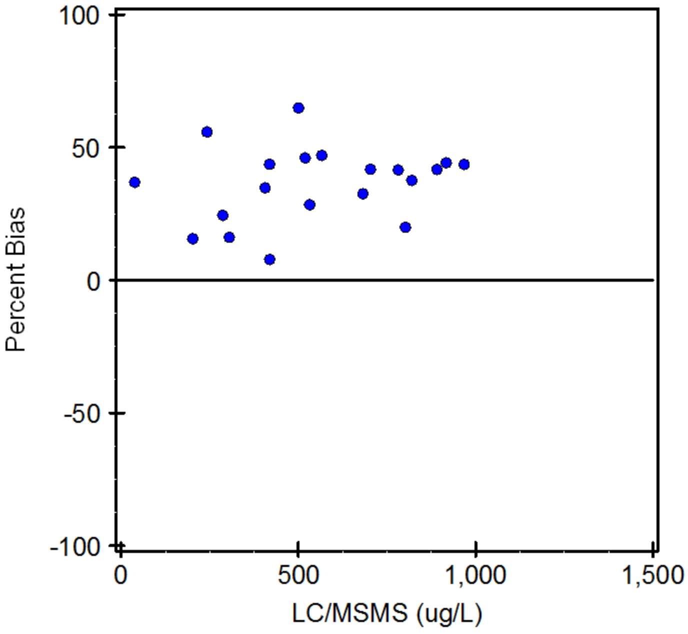

This page contains a larger, working copy of the same graphs that appear on page 1.

Copyright 1991-2005 David G. Rhoads Associates, Inc.

Alternate (Quantitative) Method Comparison

X Method: LC/MSMS **Y Method: i2000**

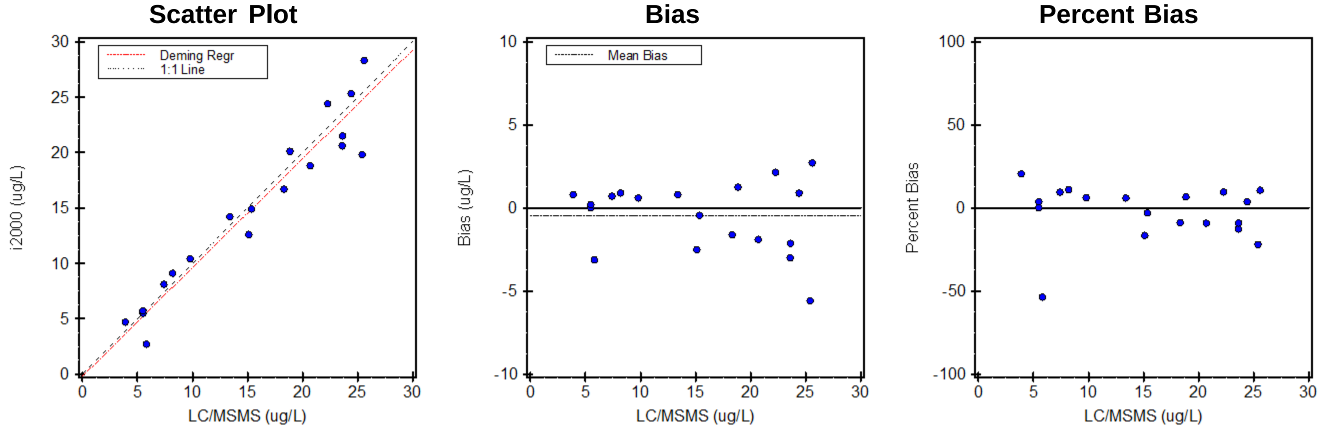

Regression Analysis

	Deming	Regular
**Slope:**	0.982 (0.854 to 1.110)	0.949 (0.822 to 1.075)
**Intercept:**	-0.20 (-2.34 to 1.94)	0.30 (-1.83 to 2.42)
**Std Err Est:**	2.09	2.07

95% Confidence Intervals are shown in parentheses

Supporting Statistics

Corr Coef (R):	0.9656	SubRange Bounds:	None
Bias:	-0.47	Points (Plotted/Total):	20/100
X Mean ± SD:	14.91 ± 7.88	Outliers:	None
Y Mean ± SD:	14.44 ± 7.74	Scatter Plot Bounds:	None
Std Dev Diffs:	2.05		

Experiment Description

	X Method	Y Method
Expt Date:	27 May 2015	27 May 2015
Rep SD:	1	1
Result Ranges:	3.9 to 25.6	2.7 to 28.3
Units:	ug/L	ug/L
Analyst:	MK	MK
Comment:		

Accepted by: ______________________ ______________

Signature Date

Copyright 1991-2005 David G. Rhoads Associates, Inc.

Alternate (Quantitative) Method Comparison

X Method: LC/MSMS **Y Method: i2000**

Experimental Results

	Spec ID		Results X	Results Y	Bias	Calc'd Y	SEE Factor
1	UAJ6452	X		2.7			
2	UAJ6451	X		8.7			
3	UAJ6453	X		12.2			
4	UAJ6456	X		6.2			
5	UAJ6454	X		11.9			
6	UAJ6450	X		9.6			
7	UAJ6444	X		2.8			
8	UAJ6443	X		5.9			
9	UAJ6445	X		10.5			
10	UAJ6447	X		6.9			
11	UAJ6446	X		7.7			
12	UAJ6458	X		2.6			
14	UAJ6472	X		3.1			
15	UAJ6488	X		5.4			
16	ZSS39603	X		18.8			
17	UAJ6745	X		16.2			
18	UAJ6469	X		13.2			
19	UAJ6461	X		7.7			
20	UAJ6460	X		11.5			
21	UAJ6464	X		21.6			
22	UAJ6468	X		4.3			
23	UAJ6467	X		11.1			
24	UAJ6419	X		12.9			
25	UAJ6412	X		8.2			
29	UAJ6410	X		7.1			
32	UAJ6401	X		6.5			
33	UAJ6404	X		9.4			
34	UAJ6402	X		9.9			
35	UAJ6426	X		20.2			
36	UAJ6439	X		7.8			
37	UAJ6438	X		12.9			
38	UAJ6440	X		13.4			
39	UAJ6442	X		8			
40	UAJ6441	X		5.6			
42	UAJ6428	X		6.5			
43	UAJ6427	X		15.8			
44	UAJ6429	X		7.9			
45	UAJ6434	X		12			
46	UAJ6430	X		18.8			
54	YVP40704	X		9.1			
59	ZIY67902	X		3.8			
60	ZIQ08702	X		12.5			
61	ZJA38902	X		13.2			
62	ZJM20004	X		10.4			
63	ZJE32203	X		3.3			
64	ZIO88003	X		2.1			
65	YZY99904	X		21.3			
66	YYS62303	X		24.9			
68	ZIM40003	X		11.5			
70	UAJ7025	X		18.8			
71	UAJ6916	X		17.4			
72	ZRG65302	X		16.9			
73	YQX43302	X		7.2			
74	UAJ7055	X		20			
75	UAJ6903	X		15			
76	UAJ6802	X		30			
77	UAJ6794	X		19			
78	UAJ6807	X		29.1			
79	UAJ6902	X		19			
80	UAJ6853	X		15.2			
81	YRA78903	X		14.4			
82	YVH98503	X		10			
87	YVH85302	X		4.6			
91	ZKB4603	X		11.5			
92	ZQX95203	X		15.9			
124	31113015	X		15.3			
127	31197733	X		3.2			
142	UAJ5620	X		8.5			
143	UAJ5607	X		30			
144	UAJ5679	X		20.4			
145	UAJ5736	X		18.7			
146	UAJ5728	X		28.8			
153	UAJ5753	X		5.6			
154	UAJ6014	X		22.7			
157	UAJ6149	X		17			
159	UAJ5977	X		20.4			
160	UAJ5763	X		3.6			
161	UAJ5755	X		11.9			
162	UAJ5792	X		21			
164	UAJ5794	X		10.9			
186	UAJ6449		3.9	4.7	0.8	3.63	0.5
187	UAJ6424		5.5	5.5	0.0	5.20	0.1
188	UAJ6448		5.5	5.7	0.2	5.20	0.2
189	UAJ6409		5.5	5.5	0.0	5.20	0.1
190	YVM26903		5.8	2.7	-3.1	5.52	-1.4
191	UAJ6421		7.4	8.1	0.7	7.07	0.5
192	UAJ6422		8.2	9.1	0.9	7.85	0.6
193	UAJ6423		9.8	10.4	0.6	9.42	0.5
194	UAJ6436		13.4	14.2	0.8	12.96	0.6
195	YUZ03304		15.1	12.6	-2.5	14.64	-1.0
196	UAJ6475		15.3	14.9	-0.4	14.88	0.0
197	UAJ5808		18.3	16.7	-1.6	17.79	-0.5
198	UAJ6185		18.9	20.1	1.3	18.31	0.9
199	UAJ7049		20.7	18.8	-1.9	20.13	-0.6
200	UAJ6009		22.3	24.4	2.1	21.67	1.3
201	ZUH12003		23.6	20.6	-3.0	22.98	-1.1
202	UAJ6151		23.6	21.5	-2.1	23.01	-0.7
203	UAJ6030		24.4	25.3	0.9	23.77	0.7
204	YVA63003		25.4	19.8	-5.6	24.73	-2.4
205	UAJ6755		25.6	28.3	2.7	24.93	1.6

Values marked with an "X" were excluded from the calculations. Outliers "O" were also excluded.

Copyright 1991-2005 David G. Rhoads Associates, Inc.

Alternate (Quantitative) Method Comparison

X Method: LC/MSMS **Y Method: i2000**

Scatter Plot

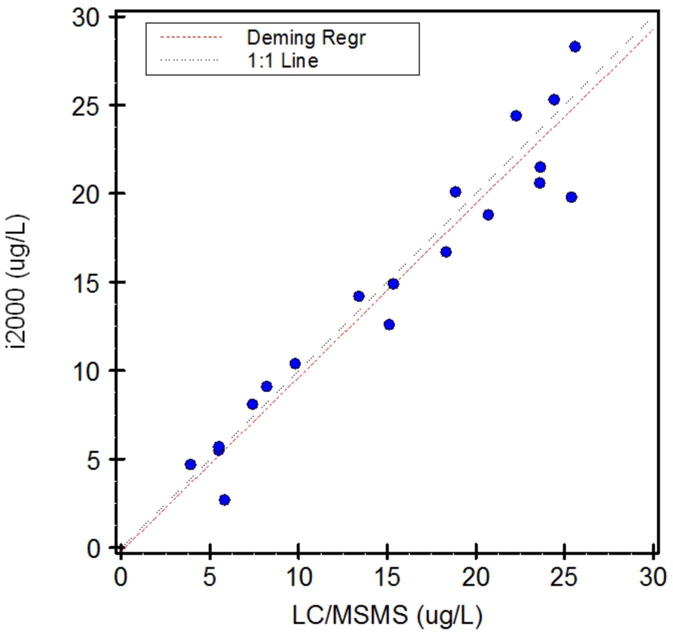

Bias

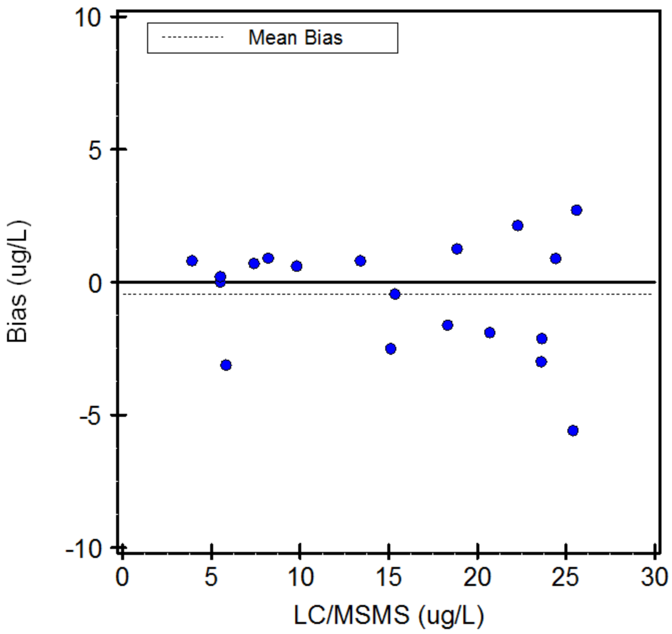

Percent Bias

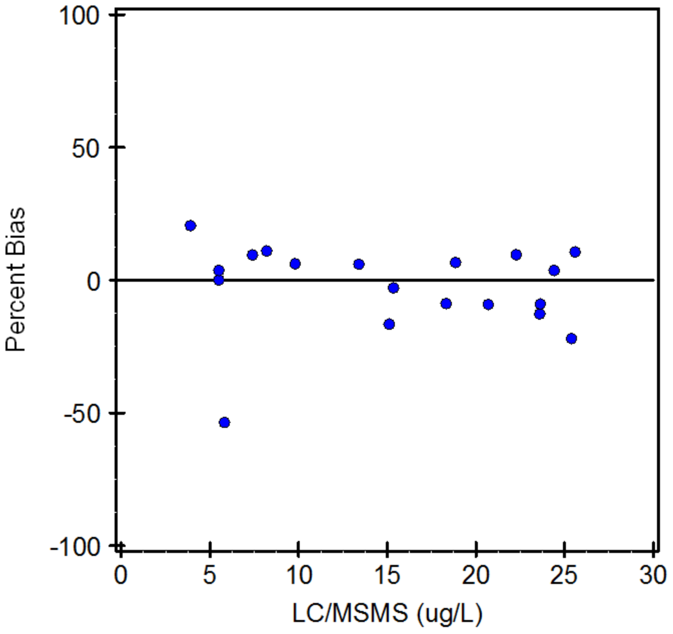

This page contains a larger, working copy of the same graphs that appear on page 1.

Copyright 1991-2005 David G. Rhoads Associates, Inc.

Alternate Method Comparison Summary

Y Method		X Method	Analyte	N	Slope	Intercept	Corr (R)
Abbott i2000	✔	LC-MS/MS	Sirolimus	20/20	1.119	1.437	0.9925
e411	✔	LC-MS/MS	Sirolimus	20/20	0.971	2.420	0.9926
e411 (Lot 2)	✔	e411 (Lot 1)	Sirolimus	20/20	0.913	0.140	0.9875
Roche e411	✔	Architect i2000	Sirolimus	100/100	0.842	0.896	0.9818

Required parameters are missing.

✔ Experiment 'passes' (or adequate number of results).

✗ Experiment 'fails' (or not enough results).

Experiment has outliers or subrange analysis.

Copyright 1991-2005 David G. Rhoads Associates, Inc.

Alternate (Quantitative) Method Comparison

X Method: LC-MS/MS **Y Method: Abbott i2000**

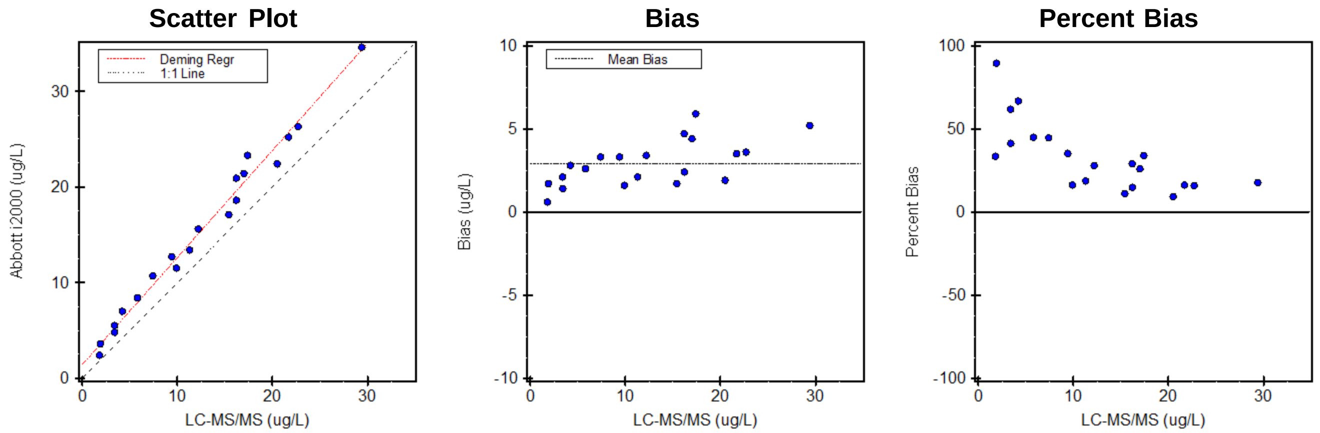

Regression Analysis

	Deming	Regular
**Slope:**	1.119 (1.051 to 1.187)	1.110 (1.042 to 1.178)
**Intercept:**	1.437 (0.450 to 2.423)	1.552 (0.568 to 2.536)
**Std Err Est:**	1.104	1.101

95% Confidence Intervals are shown in parentheses

Supporting Statistics

Corr Coef (R):	0.9925	SubRange Bounds:	None
Bias:	2.910	Points (Plotted/Total):	20/20
X Mean ± SD:	12.360 ± 7.841	Outliers:	Not Tested
Y Mean ± SD:	15.270 ± 8.768	Scatter Plot Bounds:	None
Std Dev Diffs:	1.375		

Experiment Description

	X Method	Y Method
Expt Date:	07 Jul 2016	07 Jul 2016
Rep SD:	1	1
Result Ranges:	1.80 to 29.40	2.40 to 34.60
Units:	ug/L	ug/L
Analyst:	AWF	AWF
Comment:		

Accepted by: ______________________ ______________

Signature Date

Copyright 1991-2005 David G. Rhoads Associates, Inc.

Alternate (Quantitative) Method Comparison

X Method: LC-MS/MS **Y Method: Abbott i2000**

Experimental Results

		Results			Calc'd	SEE			Results			Calc'd	SEE
	Spec ID	X	Y	Bias	Y	Factor		Spec ID	X	Y	Bias	Y	Factor
1	S00001	1.8	2.40	0.60	3.451	-1.0	11	S00011	12.2	15.60	3.40	15.091	0.5
2	S00002	1.9	3.60	1.70	3.563	0.0	12	S00012	15.4	17.10	1.70	18.672	-1.4
3	S00004	3.4	5.50	2.10	5.242	0.2	13	S00014	16.2	20.90	4.70	19.568	1.2
4	S00003	3.4	4.80	1.40	5.242	-0.4	14	S00013	16.2	18.60	2.40	19.568	-0.9
5	S00005	4.2	7.00	2.80	6.137	0.8	15	S00015	17.0	21.40	4.40	20.463	0.8
6	S00006	5.8	8.40	2.60	7.928	0.4	16	S00017	17.4	23.30	5.90	20.911	2.2
7	S00007	7.4	10.70	3.30	9.719	0.9	17	S00016	20.5	22.40	1.90	24.380	-1.8
8	S00009	9.4	12.70	3.30	11.957	0.7	18	S00018	21.7	25.20	3.50	25.723	-0.5
9	S00008	9.9	11.50	1.60	12.517	-0.9	19	S00019	22.7	26.30	3.60	26.843	-0.5
10	S00010	11.3	13.40	2.10	14.084	-0.6	20	S00020	29.4	34.60	5.20	34.341	0.2

Values marked with an "X" were excluded from the calculations.

Copyright 1991-2005 David G. Rhoads Associates, Inc.

Alternate (Quantitative) Method Comparison

X Method: LC-MS/MS **Y Method: Abbott i2000**

Scatter Plot

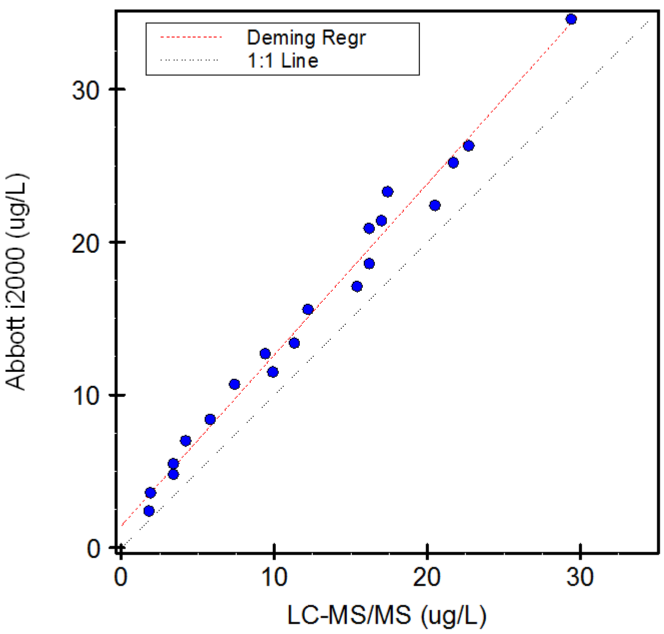

Bias

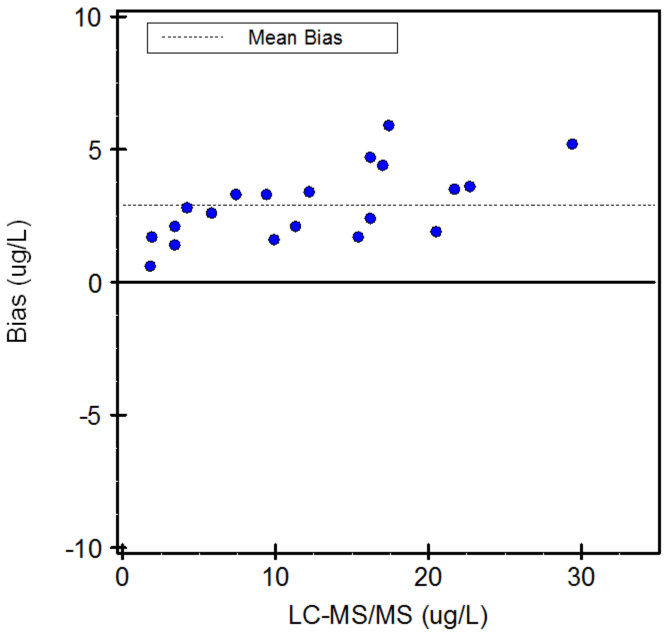

Percent Bias

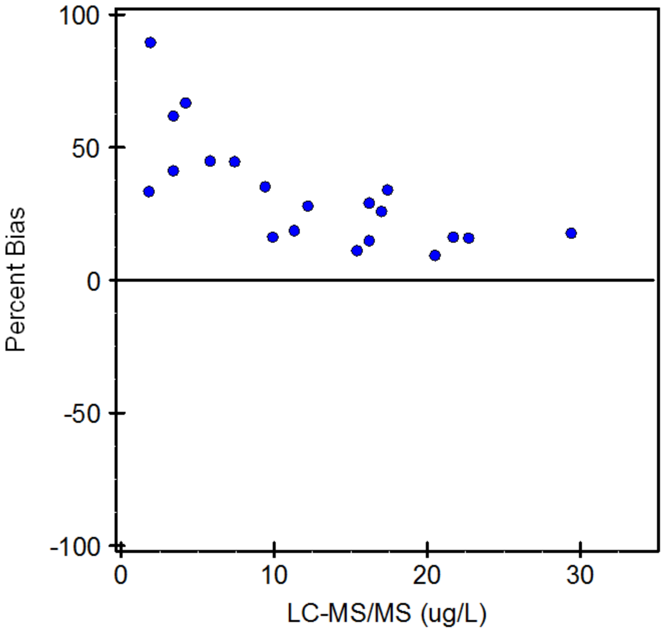

This page contains a larger, working copy of the same graphs that appear on page 1.

Copyright 1991-2005 David G. Rhoads Associates, Inc.

Alternate (Quantitative) Method Comparison

X Method: LC-MS/MS **Y Method: e411**

Scatter Plot

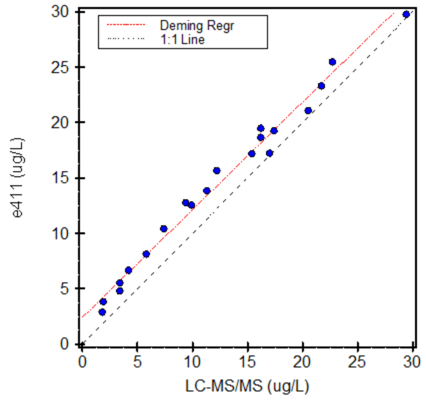

Bias

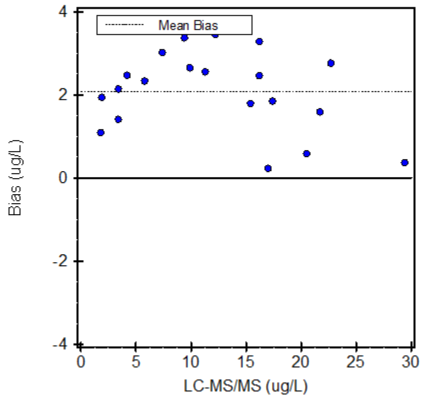

Percent Bias

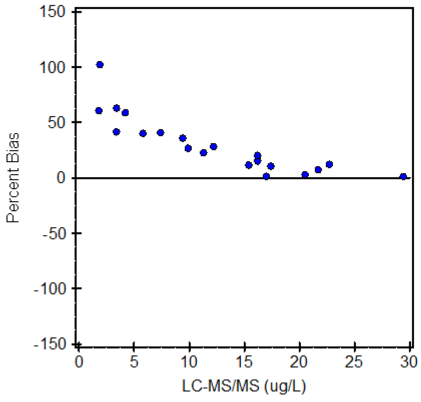

Regression Analysis

	Deming	Regular
**Slope:**	0.971 (0.913 to 1.030)	0.964 (0.906 to 1.023)
**Intercept:**	2.420 (1.571 to 3.268)	2.506 (1.659 to 3.352)
**Std Err Est:**	0.950	0.948

95% Confidence Intervals are shown in parentheses

Supporting Statistics

Corr Coef (R):	0.9926	SubRange Bounds:	None
Bias:	2.067	Points (Plotted/Total):	20/20
X Mean ± SD:	12.360 ± 7.841	Outliers:	Not Tested
Y Mean ± SD:	14.426 ± 7.618	Scatter Plot Bounds:	None
Std Dev Diffs:	0.964		

Experiment Description

	X Method	Y Method
Expt Date:	07 Jul 2016	07 Jul 2016
Rep SD:	1	1
Result Ranges:	1.80 to 29.40	2.89 to 29.77
Units:	ug/L	ug/L
Analyst:	AWF	AWF
Comment:		

Accepted by: ______________________ ______________

Signature Date

Copyright 1991-2005 David G. Rhoads Associates, Inc.

Alternate (Quantitative) Method Comparison

X Method: LC-MS/MS **Y Method: e411**

Experimental Results

	Spec ID	Results X	Results Y	Bias	Calc'd Y	SEE Factor
1	S00001	1.8	2.89	1.09	4.168	-1.3
2	S00002	1.9	3.84	1.94	4.265	-0.4
3	S00004	3.4	5.54	2.14	5.723	-0.2
4	S00003	3.4	4.81	1.41	5.723	-1.0
5	S00005	4.2	6.67	2.47	6.500	0.2
6	S00006	5.8	8.13	2.33	8.054	0.1
7	S00007	7.4	10.42	3.02	9.608	0.9
8	S00009	9.4	12.77	3.37	11.551	1.3
9	S00008	9.9	12.55	2.65	12.037	0.5
10	S00010	11.3	13.85	2.55	13.397	0.5
11	S00011	12.2	15.65	3.45	14.271	1.5
12	S00012	15.4	17.19	1.79	17.380	-0.2
13	S00014	16.2	19.48	3.28	18.157	1.4
14	S00013	16.2	18.66	2.46	18.157	0.5
15	S00015	17.0	17.23	0.23	18.934	-1.8
16	S00017	17.4	19.25	1.85	19.322	-0.1
17	S00016	20.5	21.08	0.58	22.334	-1.3
18	S00018	21.7	23.29	1.59	23.500	-0.2
19	S00019	22.7	25.46	2.76	24.471	1.0
20	S00020	29.4	29.77	0.37	30.980	-1.3

Values marked with an "X" were excluded from the calculations.

Copyright 1991-2005 David G. Rhoads Associates, Inc.

Alternate (Quantitative) Method Comparison

X Method: LC-MS/MS **Y Method: e411**

Scatter Plot

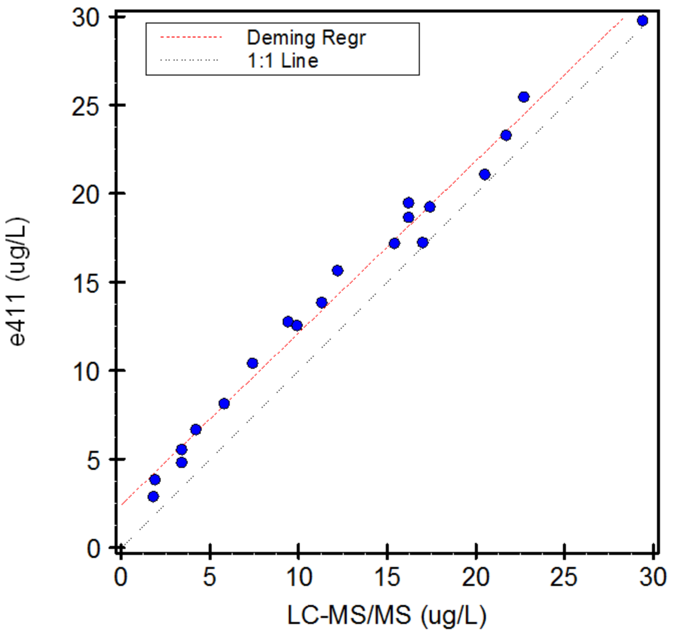

Bias

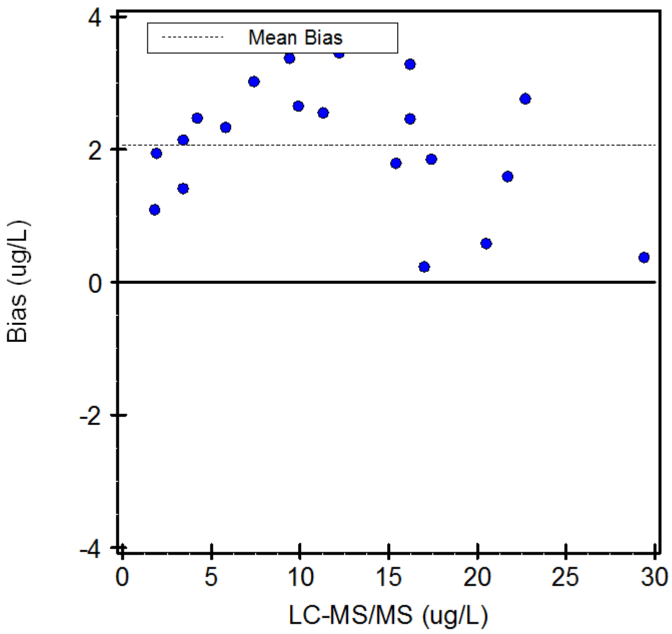

Percent Bias

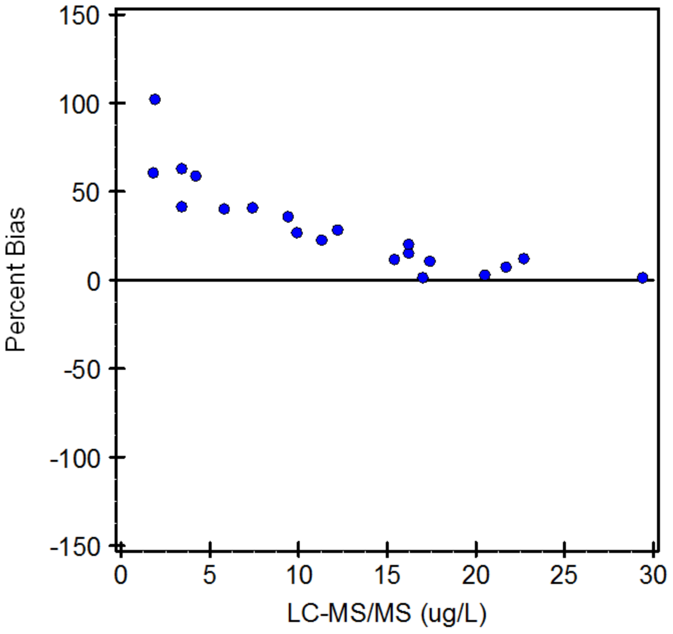

This page contains a larger, working copy of the same graphs that appear on page 1.

Copyright 1991-2005 David G. Rhoads Associates, Inc.

Alternate (Quantitative) Method Comparison

X Method: e411 (Lot 1) **Y Method: e411 (Lot 2)**

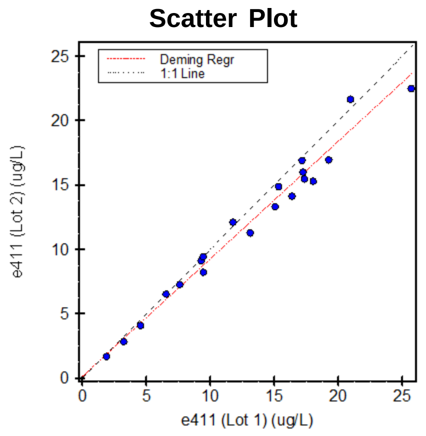

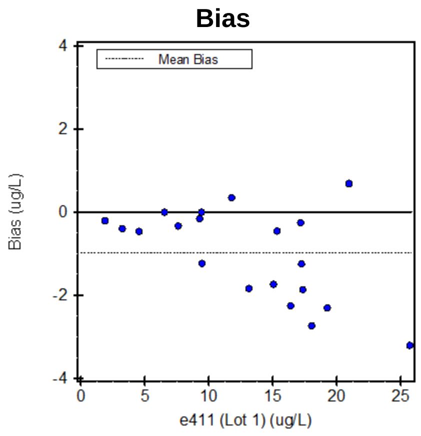

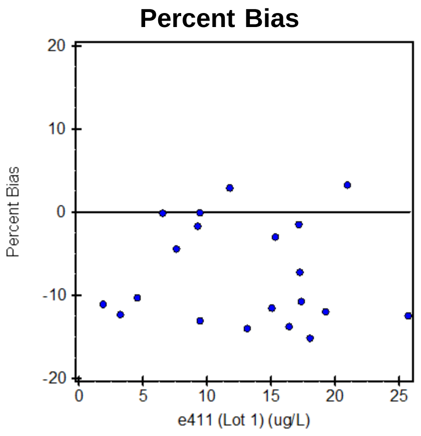

Regression Analysis

	Deming	Regular
**Slope:**	0.913 (0.841 to 0.985)	0.903 (0.831 to 0.974)
**Intercept:**	0.140 (-0.889 to 1.168)	0.275 (-0.751 to 1.300)
**Std Err Est:**	0.943	0.940

95% Confidence Intervals are shown in parentheses

Supporting Statistics

Corr Coef (R):	0.9875	SubRange Bounds:	None
Bias:	-0.988	Points (Plotted/Total):	20/20
X Mean ± SD:	12.966 ± 6.346	Outliers:	Not Tested
Y Mean ± SD:	11.978 ± 5.801	Scatter Plot Bounds:	None
Std Dev Diffs:	1.104		

Experiment Description

	X Method	Y Method
Expt Date:	05 Jul 2016	05 Jul 2016
Rep SD:	1	1
Result Ranges:	1.89 to 25.69	1.68 to 22.48
Units:	ug/L	ug/L
Analyst:	AWF	AWF
Comment:		

Accepted by: ____________________ ____________

Signature Date

Copyright 1991-2005 David G. Rhoads Associates, Inc.

Alternate (Quantitative) Method Comparison

X Method: e411 (Lot 1) **Y Method: e411 (Lot 2)**

Experimental Results

	Spec ID	Results X	Results Y	Bias	Calc'd Y	SEE Factor
1	S00001	1.89	1.68	-0.21	1.865	-0.2
2	S00002	3.24	2.84	-0.40	3.098	-0.3
3	S00003	4.55	4.08	-0.47	4.294	-0.2
4	S00005	6.54	6.53	-0.01	6.111	0.4
5	S00004	7.61	7.27	-0.34	7.088	0.2
6	S00008	9.28	9.12	-0.16	8.613	0.5
7	S00006	9.44	9.43	-0.01	8.759	0.7
8	S00007	9.46	8.22	-1.24	8.777	-0.6
9	S00010	11.77	12.11	0.34	10.886	1.3
10	S00009	13.12	11.28	-1.84	12.119	-0.9
11	S00011	15.05	13.31	-1.74	13.881	-0.6
12	S00012	15.33	14.87	-0.46	14.136	0.8
13	S00014	16.39	14.13	-2.26	15.104	-1.0
14	S00017	17.16	16.90	-0.26	15.807	1.2
15	S00016	17.23	15.98	-1.25	15.871	0.1
16	S00013	17.34	15.47	-1.87	15.972	-0.5
17	S00018	18.03	15.29	-2.74	16.602	-1.4
18	S00015	19.25	16.94	-2.31	17.715	-0.8
19	S00019	20.95	21.63	0.68	19.268	2.5
20	S00020	25.69	22.48	-3.21	23.595	-1.2

Values marked with an "X" were excluded from the calculations.

Copyright 1991-2005 David G. Rhoads Associates, Inc.

Alternate (Quantitative) Method Comparison

X Method: e411 (Lot 1) **Y Method: e411 (Lot 2)**

Scatter Plot

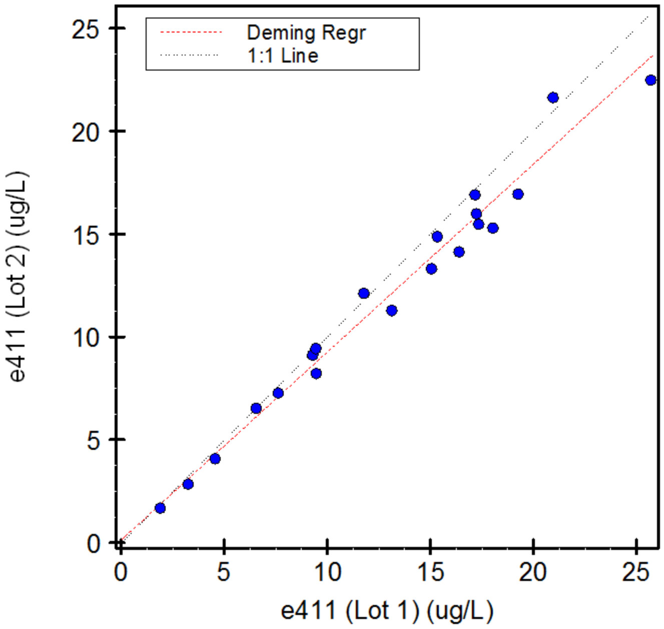

Bias

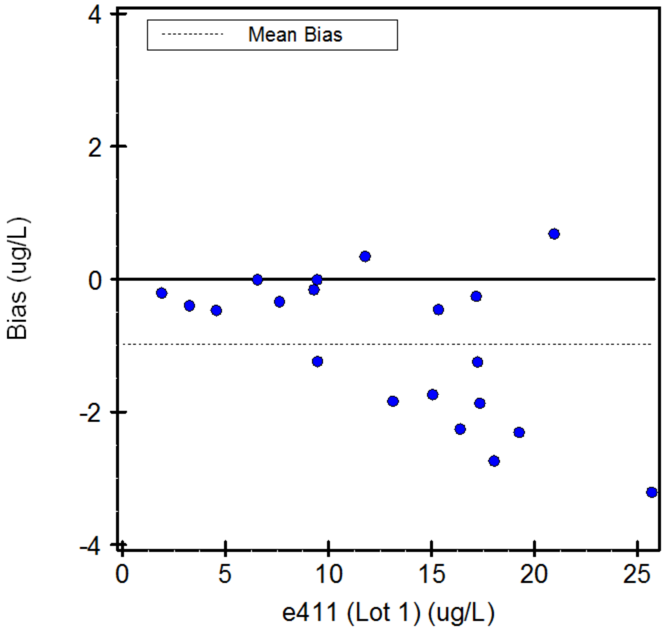

Percent Bias

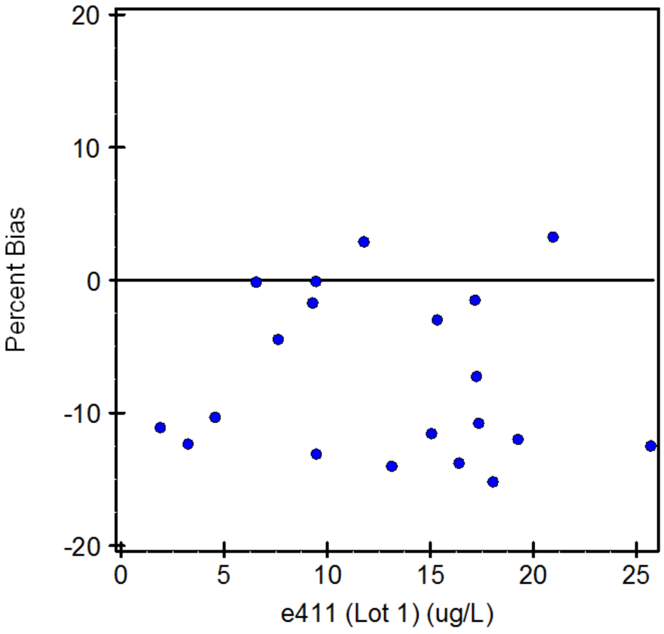

This page contains a larger, working copy of the same graphs that appear on page 1.

Copyright 1991-2005 David G. Rhoads Associates, Inc.

Alternate (Quantitative) Method Comparison

X Method: Architect i2000 **Y Method: Roche e411**

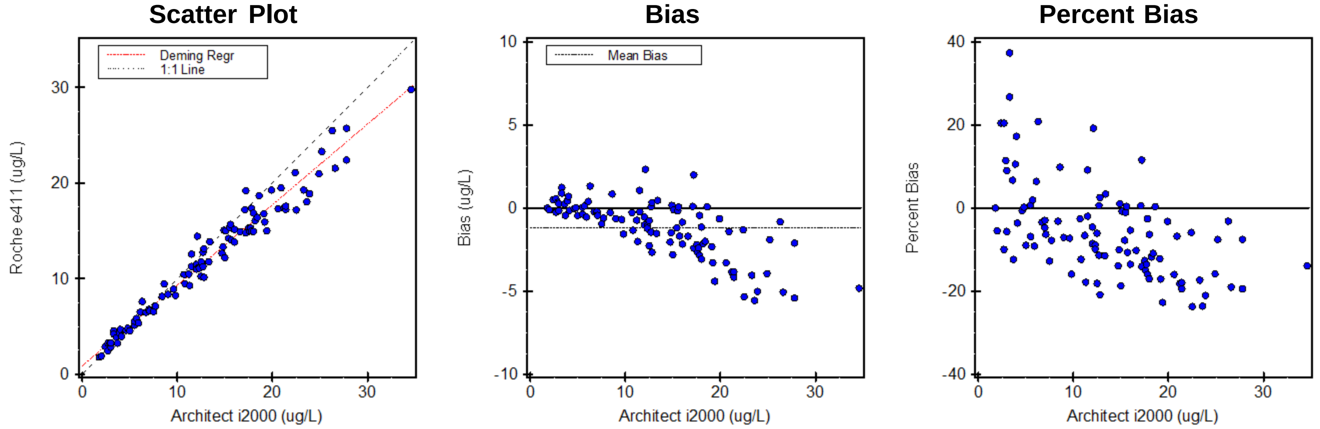

Regression Analysis

	Deming	Regular
**Slope:**	0.842 (0.810 to 0.874)	0.829 (0.797 to 0.861)
**Intercept:**	0.896 (0.410 to 1.383)	1.065 (0.580 to 1.550)
**Std Err Est:**	1.176	1.173

95% Confidence Intervals are shown in parentheses

Supporting Statistics

Corr Coef (R):	0.9818	SubRange Bounds:	None
Bias:	-1.197	Points (Plotted/Total):	100/100
X Mean ± SD:	13.230 ± 7.270	Outliers:	Not Tested
Y Mean ± SD:	12.033 ± 6.139	Scatter Plot Bounds:	None
Std Dev Diffs:	1.705		

Experiment Description

	X Method	Y Method
Expt Date:	07 Jul 2016	07 Jul 2016
Rep SD:	1	1
Result Ranges:	1.80 to 34.60	1.80 to 29.77
Units:	ug/L	ug/L
Analyst:	AWF	AWF
Comment:		

Accepted by: ______________________________ ______________

Signature Date

Copyright 1991-2005 David G. Rhoads Associates, Inc.

Alternate (Quantitative) Method Comparison

X Method: Architect i2000 **Y Method: Roche e411**

Experimental Results

		Results			Calc'd	SEE
	Spec ID	X	Y	Bias	Y	Factor
1	S00001	1.80	1.80	0.00	2.412	-0.5
2	S00002	2.00	1.89	-0.11	2.580	-0.6
3	S00003	2.40	2.89	0.49	2.917	0.0
4	S00005	2.70	3.25	0.55	3.169	0.1
5	S00004	2.70	2.43	-0.27	3.169	-0.6
6	S00006	2.90	3.23	0.33	3.338	-0.1
7	S00008	3.00	3.27	0.27	3.422	-0.1
8	S00007	3.00	2.83	-0.17	3.422	-0.5
9	S00010	3.30	4.18	0.88	3.674	0.4
10	S00009	3.30	4.53	1.23	3.674	0.7
11	S00011	3.60	3.84	0.24	3.927	-0.1
12	S00012	3.70	3.24	-0.46	4.011	-0.7
13	S00013	3.90	4.31	0.41	4.179	0.1
14	S00014	4.00	4.69	0.69	4.263	0.4
15	S00015	4.10	3.95	-0.15	4.348	-0.3
16	S00016	4.60	4.57	-0.03	4.769	-0.2
17	S00017	4.80	4.81	0.01	4.937	-0.1
18	S00018	5.00	4.55	-0.45	5.105	-0.5
19	S00020	5.50	5.54	0.04	5.526	0.0
20	S00019	5.50	5.12	-0.38	5.526	-0.3
21	S00021	5.70	5.81	0.11	5.694	0.1
22	S00022	5.90	5.36	-0.54	5.863	-0.4
23	S00023	6.10	6.49	0.39	6.031	0.4
24	S00024	6.30	7.61	1.31	6.200	1.2
25	S00025	6.70	6.47	-0.23	6.536	-0.1
26	S00027	7.00	6.79	-0.21	6.789	0.0
27	S00026	7.00	6.67	-0.33	6.789	-0.1
28	S00028	7.10	6.65	-0.45	6.873	-0.2
29	S00029	7.50	6.54	-0.96	7.210	-0.6
30	S00030	7.70	7.10	-0.60	7.378	-0.2
31	S00031	8.40	8.13	-0.27	7.967	0.1
32	S00032	8.60	9.44	0.84	8.136	1.1
33	S00033	9.00	8.36	-0.64	8.472	-0.1
34	S00034	9.60	8.90	-0.70	8.977	-0.1
35	S00035	9.80	8.24	-1.56	9.146	-0.8
36	S00036	10.70	10.42	-0.28	9.903	0.4
37	S00037	10.80	9.46	-1.34	9.988	-0.4
38	S00038	11.20	10.46	-0.74	10.324	0.1
39	S00039	11.30	9.28	-2.02	10.408	-1.0
40	S00041	11.50	11.27	-0.23	10.577	0.6
41	S00040	11.50	12.55	1.05	10.577	1.7
42	S00043	12.00	10.97	-1.03	10.998	0.0
43	S00042	12.00	11.45	-0.55	10.998	0.4
44	S00044	12.10	14.42	2.32	11.082	2.8
45	S00046	12.30	11.08	-1.22	11.250	-0.1
46	S00045	12.30	11.20	-1.10	11.250	0.0
47	S00048	12.50	11.74	-0.76	11.419	0.3
48	S00047	12.50	10.23	-2.27	11.419	-1.0
49	S00050	12.70	12.77	0.07	11.587	1.0
50	S00049	12.70	11.25	-1.45	11.587	-0.3
51	S00052	12.80	13.12	0.32	11.671	1.2
52	S00051	12.80	10.13	-2.67	11.671	-1.3
53	S00053	13.30	11.77	-1.53	12.092	-0.3
54	S00054	13.40	13.85	0.45	12.176	1.4
55	S00055	14.70	12.65	-2.05	13.271	-0.5
56	S00056	14.80	13.31	-1.49	13.355	0.0
57	S00057	14.90	15.05	0.15	13.439	1.4
58	S00059	15.00	12.19	-2.81	13.523	-1.1
59	S00060	15.10	14.98	-0.12	13.607	1.2
60	S00062	15.40	14.20	-1.20	13.860	0.3
61	S00063	15.50	15.33	-0.17	13.944	1.2
62	S00064	15.60	15.65	0.05	14.028	1.4
63	S00065	15.70	14.02	-1.68	14.112	-0.1
64	S00067	16.00	13.83	-2.17	14.365	-0.5
65	S00066	16.00	15.14	-0.86	14.365	0.7
66	S00068	16.60	14.90	-1.70	14.870	0.0
67	S00069	17.10	17.19	0.09	15.291	1.6
68	S00071	17.20	14.78	-2.42	15.375	-0.5
69	S00070	17.20	19.18	1.98	15.375	3.2
70	S00072	17.50	15.29	-2.21	15.628	-0.3
71	S00073	17.60	14.95	-2.65	15.712	-0.6
72	S00074	17.70	15.30	-2.40	15.796	-0.4
73	S00076	17.80	17.34	-0.46	15.880	1.2
74	S00075	17.80	14.95	-2.85	15.880	-0.8
75	S00078	18.00	16.85	-1.15	16.048	0.7
76	S00077	18.00	14.93	-3.07	16.048	-1.0
77	S00079	18.20	16.05	-2.15	16.217	-0.1
78	S00080	18.40	16.39	-2.01	16.385	0.0
79	S00081	18.60	18.66	0.06	16.553	1.8
80	S00082	19.10	16.77	-2.33	16.974	-0.2
81	S00083	19.20	15.91	-3.29	17.059	-1.0
82	S00084	19.40	14.99	-4.41	17.227	-1.9
83	S00085	19.90	19.25	-0.65	17.648	1.4
84	S00086	20.60	17.30	-3.30	18.237	-0.8
85	S00087	20.90	19.48	-1.42	18.490	0.8
86	S00088	21.20	17.34	-3.86	18.742	-1.2
87	S00090	21.40	17.56	-3.84	18.910	-1.1
88	S00089	21.40	17.23	-4.17	18.910	-1.4
89	S00091	22.40	21.08	-1.32	19.752	1.1
90	S00092	22.50	17.16	-5.34	19.836	-2.3
91	S00093	23.30	19.25	-4.05	20.510	-1.1
92	S00094	23.60	18.03	-5.57	20.762	-2.3
93	S00095	23.90	18.87	-5.03	21.015	-1.8
94	S00096	24.90	20.95	-3.95	21.857	-0.8
95	S00097	25.20	23.29	-1.91	22.109	1.0
96	S00098	26.30	25.46	-0.84	23.035	2.1
97	S00099	26.60	21.53	-5.07	23.288	-1.5
98	S00101	27.80	25.69	-2.11	24.298	1.2
99	S00100	27.80	22.39	-5.41	24.298	-1.6
100	S00102	34.60	29.77	-4.83	30.022	-0.2

Values marked with an "X" were excluded from the calculations.

Copyright 1991-2005 David G. Rhoads Associates, Inc.

Alternate (Quantitative) Method Comparison

X Method: Architect i2000 **Y Method: Roche e411**

Scatter Plot

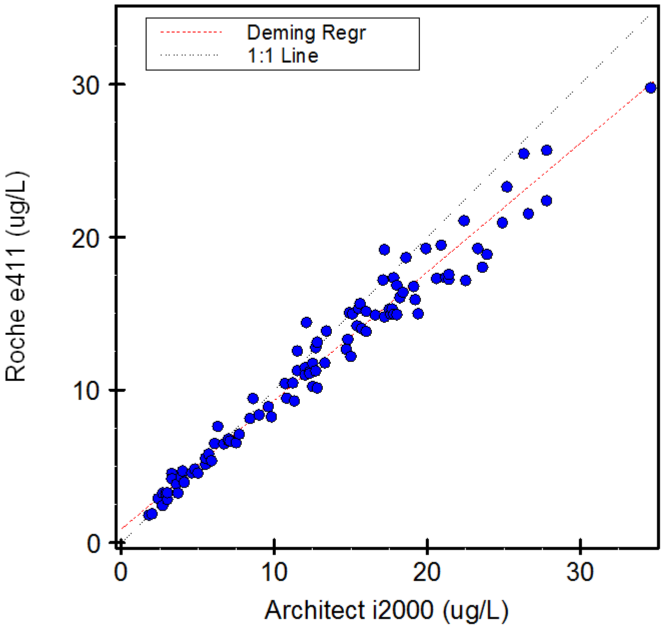

Bias

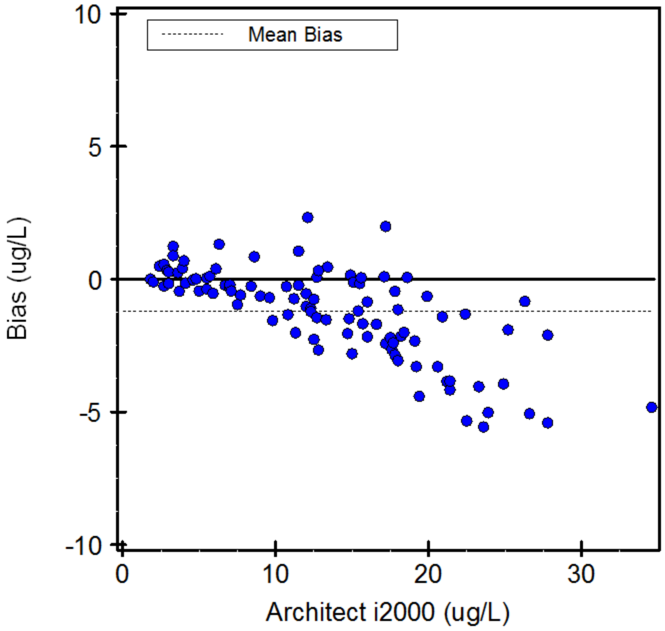

Percent Bias

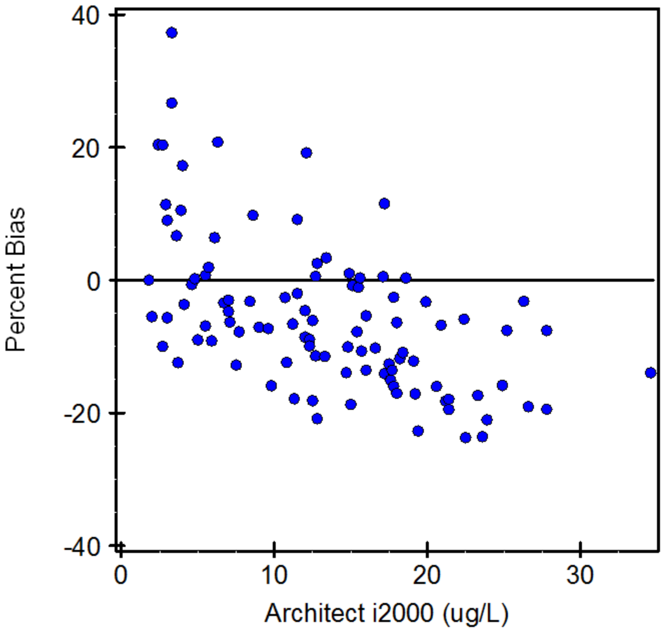

This page contains a larger, working copy of the same graphs that appear on page 1.

Copyright 1991-2005 David G. Rhoads Associates, Inc.
