# Supplementary material for: Evaluation of electrochemiluminescence immunoassays for immunosuppressive drugs on the Roche cobas e411 analyzer
Source: F1000Res. 2017 Dec 12;6:1832. Originally published 2017 Oct 13. [Version 2] doi: 10.12688/f1000research.12775.2 (PMC5710386; doi:10.12688/f1000research.12775.2)
Supplement: File containing the raw data and a table of contents for the data file [file f1000research-6-14595-s0000.tgz › 5482c4c4-1da7-4278-a215-5152bd6e3c64_Dataset_1.zip!/Dataset 1/Table of contents.pdf]

Raw Data File

for the "Evaluation of electrochemiluminescence immunoassays for immunosuppressive drugs on the Roche cobas e411 analyzer"

by Angela W.S. Fung[1,2,*], Michael J. Knauer[1,3,*], Ivan M. Blasutig[1,4], David A. Colantonio[1,5], Vathany Kulasingam[1,6,#]

Page in PDF document	Heading	Description
1	Imprecision data summary for cyclosporine and tacrolimus	Three levels of manufacturer multi-analyte QC materials (Roche Diagnostics PreciControl ISD levels 1, 2, and 3) and third-party multi-analyte QC materials (Bio-Rad Lyphochek Whole Blood ISD Controls levels 1, 3, and 4) were analyzed. QC samples were prepared and measured in duplicate, one run per day over 10 days (total of n=20 per analyte). Simple precision was calculated.
2 – 7	Imprecision data for cyclosporine	
8 – 15	Imprecision data for tacrolimus	
16 – 24	Imprecision summary and data for sirolimus	
25	Linearity data summary for cyclosporine and tacrolimus	CSA and TAC linearity were assessed using CAP EQA linearity materials (6 concentrations measured in duplicate). SRL linearity was assessed using a patient sample above the upper measuring range diluted with blank whole blood to 6 concentrations and measured in duplicate.<br>Linearity and residual plot.
26 – 27	Linearity data for cyclosporine	
28 - 31	Linearity data for tacrolimus	
32 – 34	Linearity data for sirolimus	
35	Functional sensitivity data summary	Residual patient sample with levels 2-3 times the claimed limit of quantification (LoQ) was used to generate a series of dilutions with blank whole blood. The neat sample and dilutions were measured in triplicates within one day. The precision profile curve was used to calculate the LoQ

		concentration corresponding to a CV of 20% with the upper 95% confidence limit.
36 – 37	Functional sensitivity data for cyclosporine	
38 – 39	Functional sensitivity data for sirolimus	
40 – 42	Functional sensitivity data for tacrolimus	
43	Method Comparison data summary for cyclosporine and tacrolimus	Method comparison experiments were assessed where anonymized residual patient samples spanning the analytical measuring range for each analyte were measured one replicate per method. 100 samples were compared between Roche ECLIA and Abbott CMIA ISD immunoassays.<br>A subset of 20 samples were also measured by LC-MS/MS ISD method.<br>Another subset of 20 samples were used to evaluate lot-to-lot assessment between two lots of reagents for each ISD.<br>Deming regression and Bland-Altman plot analysis.
44 – 46	Method comparison for cyclosporine	Roche ECLIA vs Abbott CMIA CSA (n=100)
47 – 49	Method comparison for tacrolimus	Roche ECLIA vs Abbott CMIA TAC (n=100)
50 - 52	Method comparison for cyclosporine	Roche ECLIA vs LC-MS/MS CSA (n=20)
53 - 55	Method comparison for tacrolimus	Roche ECLIA vs LC-MS/MS TAC (n=20)
56 – 58	Lot-to-lot comparison for cyclosporine	Lot-to-lot comparison for Roche ECLIA CSA (n=20)

59 – 61	Lot-to-lot comparison for tacrolimus	Lot-to-lot comparison for Roche ECLIA TAC (n=20)
62 - 64	Method comparison for cyclosporine	Abbott CMIA vs LC-MS/MS CSA (n=20)
65 - 67	Method comparison for tacrolimus	Abbott CMIA vs LC-MS/MS TAC (n=20)
68	Method Comparison data summary for sirolimus	Method comparison experiments were assessed where anonymized residual patient samples spanning the analytical measuring range for each analyte were measured one replicate per method. 100 samples were compared between Roche ECLIA and Abbott CMIA ISD immunoassays.<br>A subset of 20 samples were also measured by LC-MS/MS ISD method.<br>Another subset of 20 samples were used to evaluate lot-to-lot assessment between two lots of reagents for each ISD.<br>Deming regression and Bland-Altman plot analysis.
69 - 71	Method comparison for sirolimus	Abbott CMIA vs LC-MS/MS SRL (n=20)
72 - 74	Method comparison for sirolimus	Roche ECLIA vs LC-MS/MS SRL (n=20)
75 - 77	Lot-to-lot comparison for sirolimus	Lot-to-lot comparison for Roche ECLIA SRL (n=20)
78 - 80	Method comparison for sirolimus	Roche ECLIA vs Abbott CMIA SRL (n=100)
