# Supplementary material for: Evaluation of electrochemiluminescence immunoassays for immunosuppressive drugs on the Roche cobas e411 analyzer
Source: F1000Res. 2017 Dec 12;6:1832. Originally published 2017 Oct 13. [Version 2] doi: 10.12688/f1000research.12775.2 (PMC5710386; doi:10.12688/f1000research.12775.2)
Supplement: Peer review history

# Peer review: Referee response for version 2

**DOI:** 10.5256/f1000research.14595.r29428

**Published:** 2018-01-03

**Authors:** Stephen A. Hill

**Affiliations:** 1 Department of Pathology and Molecular Medicine, McMaster University, Hamilton, ON, Canada

1. “The ECLIA methods offer a wider analytical measurement range for CSA and TAC than CMAI methods.” It would be helpful to include the upper limits for the CMIA assays for comparison.
2. “The claimed functional sensitivity of the ECLAI ISD methods are improved for TAC and SRL compared to CMIA ISD methods.” It would be helpful to include the CMIA data for comparison.
3. “Overall, all three ECLID ISDs met our acceptance criteria, with SRL slightly exceeding the limit for slope.” The two parts of this sentence are incongruent. SRL did not meet your pre-determined criteria. It is inappropriate to set pre-determined acceptance criteria and then not use them.
  “In conclusion, the overall analytical evaluation of ELCIA method for CSA, TAC and SRL met acceptable performance” The same comment applies here.
  One of the strengths of this paper is that pre-determined criteria were established.
  I think it’s fair to state the in spite of not meeting all pre-determined performance criteria, the ECLIA methods are of sufficient quality to accept them for clinical service.  
  Some re-wording should correct this.

I have read this submission. I believe that I have an appropriate level of expertise to confirm that it is of an acceptable scientific standard, however I have significant reservations, as outlined above.

# Peer review: Referee response for version 1

**DOI:** 10.5256/f1000research.13840.r26927

**Published:** 2017-11-30

**Authors:** Julie Shaw

**Affiliations:** 1 Department of Pathology and Laboratory Medicine, The Ottawa Hospital, Eastern Ontario Regional Laboratories Association, University of Ottawa, Ottawa, ON, Canada

Here the authors describe evaluation of three immunosuppressant drug immunoassays on the Roche Cobas e411 analzyer. The authors evaluated the performance of assays for cyclosporine, tacrolimus and sirolimus on the system, which included imprecision, linearity and functional sensitivity studies as well as method comparisons and lot-to-lot comparisons. They compared the assay performance to the performance of the same assays on the Abbott immunoassay system and to LC MS/MS assays for the same analytes.

 I have a few comments that, if the authors could address, I feel would strengthen the manuscript. 

1. In the methods section, it’s not clear to me whether the measurements were performed using the Roche, Abbott and LC-MS/MS methods on the same day. It is mentioned that some specimens were stored prior to analysis. Could the authors clarify this?
2. What are the immunoassays and LC-MS/MS methods traceable to? Is there a standard? This information would be helpful.
3. The authors mention that the Roche assays were calibrated five times over the course of the study. Is this in fitting with the manufacturer’s recommendations? How does this calibration frequency compare to the Abbott system?
4. The authors should discuss the limitation of performing imprecision studies using only one lot number of reagent. 
5. The authors should include a description of the LC MS/MS method that was used. There is currently no mention of this methodology until the results section. 
6. There are several grammatical errors throughout the manuscript and I would recommend that the authors read it through carefully to correct these.

I have read this submission. I believe that I have an appropriate level of expertise to confirm that it is of an acceptable scientific standard.

# Peer review: Referee response for version 1

**DOI:** 10.5256/f1000research.13840.r26931

**Published:** 2017-10-20

**Authors:** Jennifer Shea

**Affiliations:** 1 Laboratory Medicine, Saint John Regional Hospital, Horizon Health Network, Saint John, NB, Canada

Fung et al. present data evaluating three new ECLIA assays for the immunosuppressant drugs cyclosporine, tacrolimus, and sirolimus on a Roche Cobas e411 analyzer. Overall the study design is sound generally following CLSI guidelines for method validation.  The introduction provides a thorough background on the drugs/assays being evaluated and the methods section clearly describes each portion of the method validation that would easily allow another researcher to replicate the study if desired.  The results/discussion section succinctly presents their data in a cohesive and systematic manner and the authors do a  great job at highlighting the novel aspects of their study. Their conclusion that these three new assays are fit for use for therapeutic drug monitoring is supported by the data presented.

I have read this submission. I believe that I have an appropriate level of expertise to confirm that it is of an acceptable scientific standard.
